# Proteomic Profiling of Human Keratinocytes Undergoing UVB-Induced Alternative Differentiation Reveals TRIpartite Motif Protein 29 as a Survival Factor

**DOI:** 10.1371/journal.pone.0010462

**Published:** 2010-05-03

**Authors:** Véronique Bertrand-Vallery, Nathalie Belot, Marc Dieu, Edouard Delaive, Noëlle Ninane, Catherine Demazy, Martine Raes, Michel Salmon, Yves Poumay, Florence Debacq-Chainiaux, Olivier Toussaint

**Affiliations:** 1 Research Unit of Cellular Biology, University of Namur (FUNDP), Namur, Belgium; 2 StratiCELL SA, Isnes, Belgium; 3 Cell and Tissue Laboratory, URPHYM, University of Namur (FUNDP), Namur, Belgium; Tufts University, United States of America

## Abstract

**Background:**

Repeated exposures to UVB of human keratinocytes lacking functional p16^INK-4a^ and able to differentiate induce an alternative state of differentiation rather than stress-induced premature senescence.

**Methodology/Principal Findings:**

A 2D-DIGE proteomic profiling of this alternative state of differentiation was performed herein at various times after the exposures to UVB. Sixty-nine differentially abundant protein species were identified by mass spectrometry, many of which are involved in keratinocyte differentiation and survival. Among these protein species was TRIpartite Motif Protein 29 (TRIM29). Increased abundance of TRIM29 following UVB exposures was validated by Western blot using specific antibody and was also further analysed by immunochemistry and by RT-PCR. TRIM29 was found very abundant in keratinocytes and reconstructed epidermis. Knocking down the expression of TRIM29 by short-hairpin RNA interference decreased the viability of keratinocytes after UVB exposure. The abundance of involucrin mRNA, a marker of late differentiation, increased concomitantly. In TRIM29-knocked down reconstructed epidermis, the presence of picnotic cells revealed cell injury. Increased abundance of TRIM29 was also observed upon exposure to DNA damaging agents and PKC activation. The UVB-induced increase of TRIM29 abundance was dependent on a PKC signaling pathway, likely PKCδ.

**Conclusions/Significance:**

These findings suggest that TRIM29 allows keratinocytes to enter a protective alternative differentiation process rather than die massively after stress.

## Introduction

Keratinocytes proliferate in the basal layer of the epidermis before moving upwards in the suprabasal layers through a differentiation program that culminates in fully differentiated dead cells of the cornified layer representing a protective barrier [1]. UVB (290-320 nm) is the most deleterious component of sunlight on earth surface [2]. DNA is the major chromophore for UVB, explaining their high mutagenicity [2], [3]. UVB also interact with cellular chromophores and photosensitizers, resulting in the generation of reactive oxygen species that cause oxidative damage and activate cellular signaling pathways related to growth, differentiation, senescence, connective tissue degradation and inflammation [3], [4]. Keratinocytes are more resistant to UV than other cell types [5] due to specialized responses [6]. However, repeated exposures to UV can lead to epidermal malignancies [2].

Repeated exposures to sublethal doses of UVB induce an alternative differentiation state rather than premature senescence in cultivated human keratinocytes lacking functional p16^INK-4a^, immortalized with telomerase and retaining their differentiation capacities (called N-hTERT cells herein) [7], [8]. While expression of telomerase does not abolish UVB-induced premature senescence in human diploid fibroblasts [9], nor in human keratinocytes [10], absence of functional p16^INK-4a^ does. In such sublethal conditions where cell death and senescence cannot take place, only alternative differentiation is observed [7]. These are unique conditions to study alternative differentiation independently of cell death and senescence. A further non negligible advantage of this unique keratinocyte cell line is also that it allows functional studies in keratinocytes still able to differentiate. This UVB-induced alternative differentiation state is characterized namely by an increased abundance of involucrin, a late marker of differentiation, and cytokeratins (K) K6, K16 and K17 [7], as also observed in primary keratinocytes and *in vivo* in the epidermis [6], [11], [12]. UV-induced alternative differentiation of keratinocytes is not yet well known and should be characterized.

In this report, proteomic profiling with fluorescent two-dimensional difference in-gel electrophoresis (2D-DIGE) until 64 h after repeated exposures to UVB allowed to identify sixty-nine differentially abundant protein species. Among the protein species with increased abundance were Capping-protein Gelsolin-like protein (CapG), TRIparite Motif Protein 29 (TRIM29) and several phosphorylated cytokeratins. Functional studies using shRNA allowed testing the involvement of TRIM29 in cell survival after exposure to UVB. We also tested whether TRIM29 expression upon exposure to UVB was PKC-dependent.

## Results

In the model used herein, N-hTERT keratinocytes expressing telomerase and lacking functional p16^INK-4a^ were exposed eight times to UVB at a dose of 300 mJ/cm^2^ per exposure as described in the materials and methods. These conditions were previously shown to be sublethal [7], which was checked again herein. Briefly, eight exposures to 300 mJ/cm^2^ UVB doses inhibited cell proliferation without any sign of lethality when compared to keratinocytes analysed before any exposure to UVB. While the repeated exposures, representing a cumulated dose of 8×300 mJ/cm^2^ = 2,400 mJ/cm^2^, were not lethal, there was significant cell death above 1,800 mJ/cm^2^ after a single exposure. Similar dose-dependent profiles of cell death were observed in N-hTERT and primary keratinocytes (not shown).

### Proteomic analysis of N-hTERT keratinocytes repeatedly exposed to UVB

Eight exposures to 300 mJ/cm^2^ UVB of N-hTERT keratinocytes expressing telomerase and lacking functional p16^INK-4a^ trigger an alternative state of differentiation uncoupled from premature senescence. This UVB-induced alternative differentiation state is characterized by an increased abundance of involucrin, a late marker of differentiation, and cytokeratins (K) K6, K16 and K17 [7]. In addition, we verified herein whether the most important markers of the response of keratinocyte to UV were observed when this particular cell line was repeatedly exposed to UVB. Western blots showed that the p38 Mitogen-Activated Protein Kinase (p38^MAPK^) and the small Heat Shock Protein 27 (HSP27) were indeed phosphorylated. Enzyme-linked immunosorbent assay and zymography showed an 11-fold increase of the secretion of active metalloprotein-9 in the culture medium (not shown). This corresponded to the data published from studies on primary cells [13], [14].

**Figure 1:**
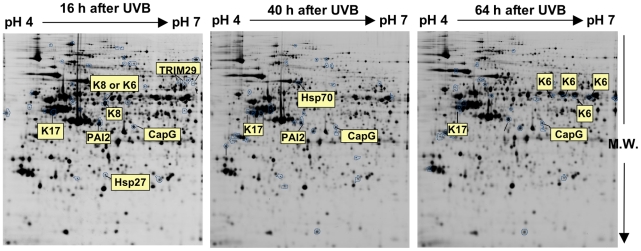
Representative 2D-DIGE profiling of N-hTERT keratinocytes repeatedly exposed to UVB and remarkable differential spots intensities. N-hTERT keratinocytes were exposed to UVB at 300 mJ/cm^2^ with 4 exposures per day for 2 days. Control samples were submitted to the same culture conditions without UVB. Three independent cultures (N = 3) were lysed at 16, 40 and 64 h after the 8^th^ exposure to UVB. Labeled samples were mixed, set to isoelectrofocusing and second-dimension was performed. The CyDye images presented correspond to a typical 2D-DIGE protein pattern [cyanine fluorophore specific (Cy2) image] (M.W. molecular weight). Spots with significantly different intensity between control and UVB exposed cells (41, 34 and 30 variations at 16, 40 and 64 h respectively) are shown by circles drawn on spot boundaries (p<0.05, Student's *t* test in the three independent experiments). These spots were identified by nanoLC-MS-MS. Only a few proteins of remarkable interest are indicated here.

In order to perform a 2D-DIGE proteomic profiling, 18 independent samples of proteins were prepared from UVB-treated and non-treated cells considered at 16, 40 and 64 h (3 UVB-treated and 3 control samples at each time) after the eight exposures to UVB. Differentially labeled control and UVB-treated samples were mixed and resolved within the same gel in a 4-7 pH range. Our conditions avoided the potentially negative effects of keratins on gel resolution. Representative images of gels are shown for each time after the last exposure to UVB. This analysis yielded respectively 41, 34 and 30 differentially intense spots at 16, 40 and 64 h after the last exposure to UVB. Those spots are circled in blue on representative 2D gels (Figure 1).

### Identification of proteins with differential abundance after repeated exposures to UVB

Among the selected spots, 69 protein species were successfully identified by LC-MS-MS. All the proteins identified are sorted in Table S1 (results section of the supplementary materials) according to their main known functions. Full information about the LC-MS-MS identifications such as peptide sequences identified for each protein is compiled in Table S2 (Table S2A, Table S2B and Table S2C in the results section of the supplementary materials). Most of the identified proteins play roles in cytoskeleton, stress defense, cell cycle progression, differentiation, metabolism, DNA binding/transcriptional activation and repression, and protease inhibition. A brief description of the role of these proteins is presented in the results section of the supplementary materials (Text S1).

Among the proteins involved in keratinocyte differentiation and identified herein are cytokeratins K6, K16, K17, plasminogen activator inhibitor-2 (PAI-2), 14-3-3-σ and phospho-heat shock protein 27 (P-HSP27). Among the most interesting candidates identified in this profiling, TRIM29, CapG and cytokeratin 8 (K8) had never been previously described as differentially abundant after exposure of keratinocytes to UVB. In the main part of this article, we focused on TRIM29. Expression data on CapG and cytokeratin 8 are shown in the results section of the supplementary materials (Figure S1).

### Increased abundance of TRIM29 in keratinocytes after exposure(s) to UVB

TRIM29, standing for TRIpartite Motif family 29, was discovered in cells from Ataxia Telangectasia (AT) patients. TRIM29 is also called ATDC for « Ataxia-Telangiectasia group D-Complementing protein ». AT is an autosomal recessive human genetic disease characterized by an increased risk of cancer [15] and caused by a mutation in the Ataxia Telangiectasia Mutated (ATM) gene, leading to a lack of ATM protein [16]. ATM is the chief activator protein triggering either DNA repair or apoptosis [17]. Cells from AT patients exhibit hyper-sensitivity to ionizing radiations, radioresistance to DNA synthesis, and cell cycle abnormalities [15]. TRIM29 was discovered from AT cells. TRIM29 partially suppresses the sensitivity to ionizing radiation [18].

**Figure 2:**
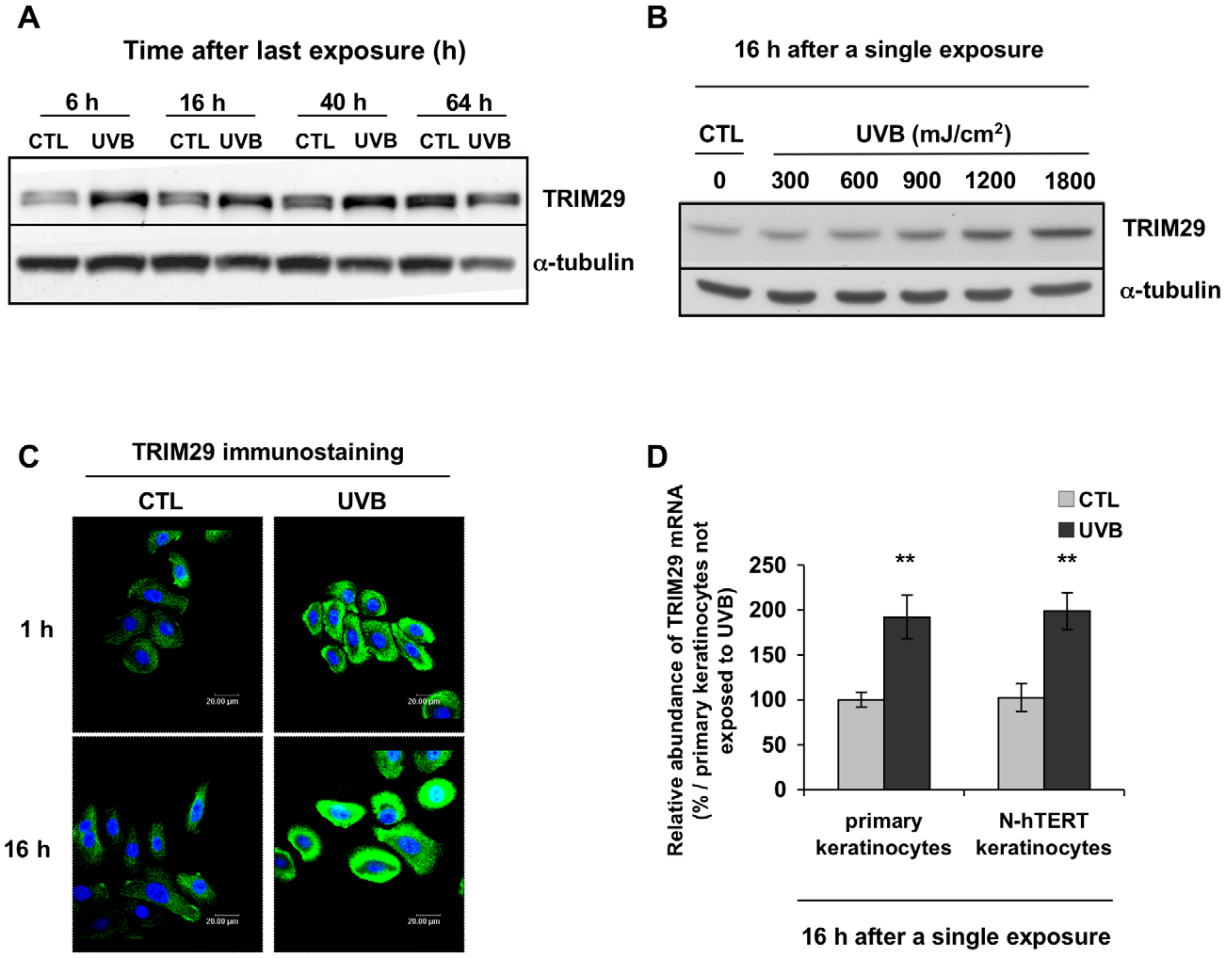
Increased abundance of TRIM29 after single or repeated exposures of N-hTERT keratinocytes to UVB. **A:** N-hTERT keratinocytes were exposed 8 times to UVB at 300 mJ/cm^2^. Control cells (CTL) were submitted to the same culture conditions without UVB. Increased abundance of TRIM29 was confirmed by Western blot using a specific anti-TRIM29 antibody at 6, 16, 40 and 64 h after the 8^th^ exposure to UVB. α-tubulin was used to assess protein loading. The results are representative of three independent experiments. **B:** Dose-dependent increase of abundance of TRIM29 in N-hTERT keratinocytes after a single exposure to UVB. At 16 h after a single exposure to various doses of UVB, Western blot was performed to detect TRIM29. α-tubulin was used as reference level. Control cells (CTL) were submitted to the same conditions without UVB. The results are representative of three independent experiments. **C:** Fluorescence micrographs of TRIM29 immunostaining (green) obtained by semi-quantitative confocal microscopy at 1 and 16 h after a single exposure to UVB at 1,200 mJ/cm^2^. Nuclei were stained with TO-PRO-3 (blue). The results are representative of three independent experiments. **D:** Relative increase in the abundance of TRIM29 mRNA in primary and in N-hTERT keratinocytes after a single exposure to UVB at 1,200 mJ/cm^2^ (UVB). Real-time RT-PCR was performed. GAPDH was chosen as housekeeping gene. The results (means of triplicates ± SD) (N = 3) are expressed as percentage of increase compared with the mRNA abundance of the respective mRNA species in control primary keratinocytes (CTL). Student's *t*-test: ** p<0.01 vs respective control cells.

In our protein profiling, TRIM29 was identified in 3 spots, with increased intensity (1.8-, 1.9- and 1.9-fold) at 16 h after 8 exposures of N-hTERT keratinocytes to UVB (Figure 1 and table S1). Western blot analysis confirmed that the abundance of TRIM29 increased in keratinocytes at 6, 16 and 40 h after the last exposure to UVB, with higher abundance at 6 h after the last exposure (Figure 2A). No increase in TRIM29 abundance was observed in N-hTERT keratinocytes exposed to a single dose of UVB at 300 mJ/cm^2^. A dose-dependent increase of TRIM29 abundance was observed above 1,200 mJ/cm^2^ (Figure 2B). Interestingly, an increase of TRIM29 abundance was already found at 1 h after a single exposure to UVB at 1,200 mJ/cm^2^ (Figure 2C). We did not observe TRIM29 in the nucleus (Figure 2C). We observed respective 2.0- and 1.8-fold increase of TRIM29 mRNA abundance in the N-hTERT cell line and in primary keratinocytes (p<0.05) at 16 h after a single exposure to UVB (Figure 2D). Thus the increased abundance of TRIM29 is not linked to the lack of functional p16^INK-4a^ nor to the expression of telomerase.

**Figure 3:**
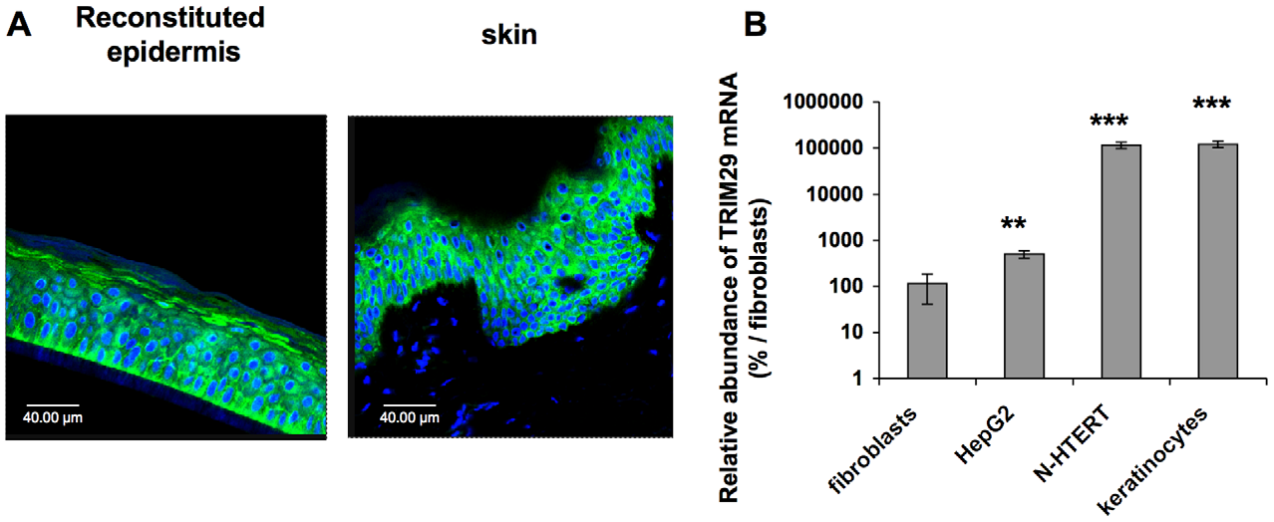
TRIM29 is strongly expressed in human epidermis. **A:** Immunofluorescent staining of TRIM29 (green) of histological sections of reconstructed epidermis and from human skin tissue were visualized by semi-quantitative confocal microscopy. Nuclei were stained with TO-PRO-3 (blue). The results are representative of three independent experiments. **B:** TRIM29 mRNA abundance is more important in keratinocytes than in other cell types. Total RNA was extracted from subconfluent N-hTERT and primary keratinocytes, from dermal fibroblasts AG04431 and from hepatocarcinoma cells (HepG2). Real-time RT-PCR was performed for human TRIM29. TRIM29 mRNA abundance in fibroblasts was considered as the reference. The results (means ± SD from triplicates) (N = 3) are presented on a logarithmic scale (** p<0.01 and *** p<0.001 vs fibroblasts).

Immunodetection of TRIM29 was possible in reconstructed epidermis and in human skin biopsies (Figure 3A). TRIM29 was not detected in the dermis of the human skin biopsies. Compared to human dermal fibroblasts *in vitro* considered as reference level where TRIM29 was barely detected, TRIM29 mRNA was 4.4-fold more abundant in the hepatocarcinoma cell line hepG2. It was respectively 164,000 and 170,000-fold more abundant in N-hTERT and primary keratinocytes (Figure 3B), suggesting an important level of expression of TRIM29 in human keratinocytes.

### Knocking down TRIM29 decreases survival of keratinocytes exposed to UVB

**Figure 4:**
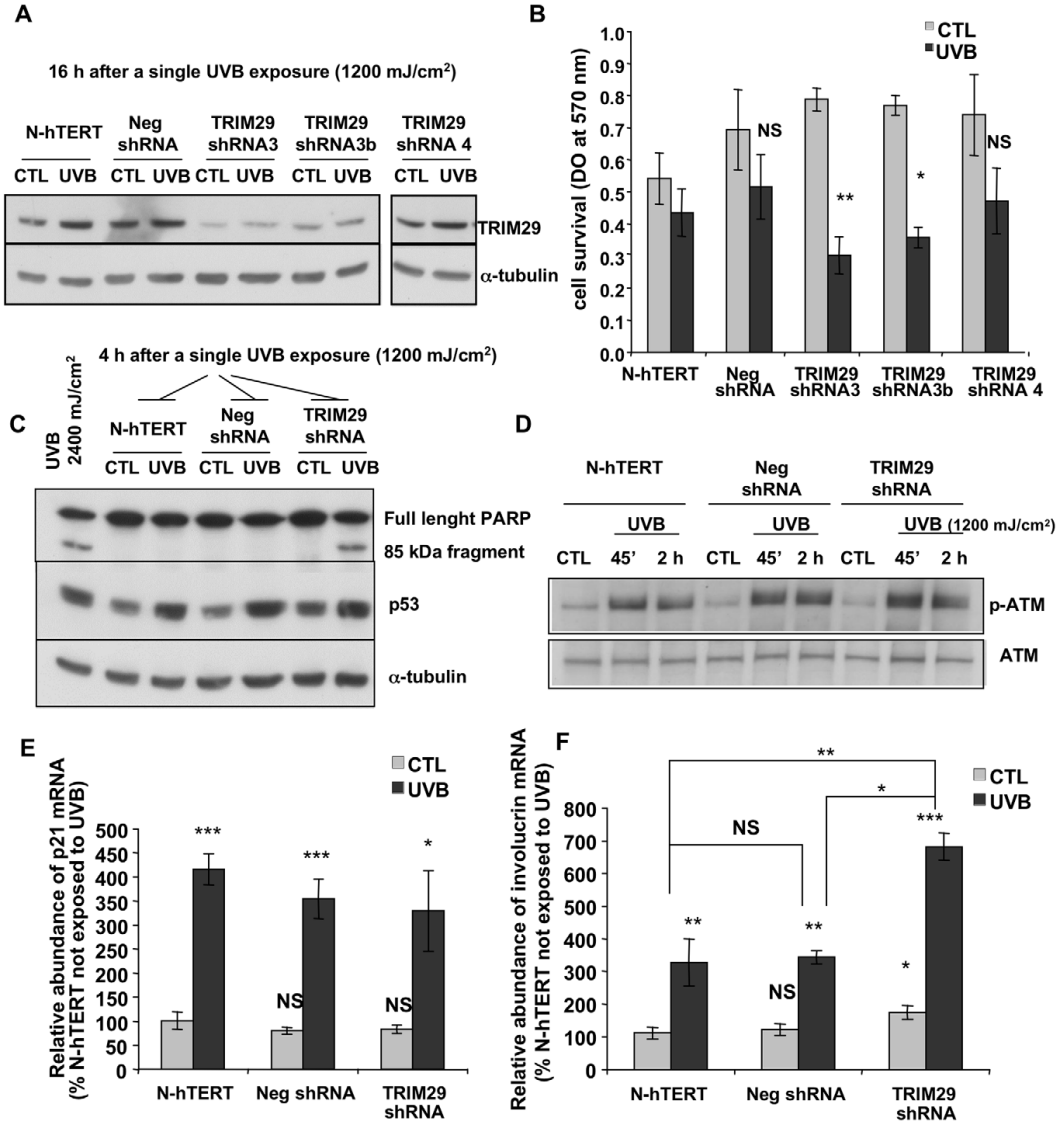
Effect of TRIM29 silencing on the cell survival after a single exposure to UVB. **A:** Western blot determined the abundance of TRIM29 in TRIM29 shRNA cell lines (3, 3b and 4), in N-hTERT parental cells and in the Neg shRNA cell line at 16 h after a single exposure to UVB at 1,200 mJ/cm^2^. α-tubulin was used to assess protein loading. **B:** Cell viability was assessed by the MTT method at 16 h after the exposure to UVB. The abundance of TRIM29 in N-hTERT cells non-exposed to UVB (CTL) was considered as reference level. Results presented are means ± S.D. (N = 4, Student's *t*-test: NS: non significant, * p<0.05, ** p<0.01 vs UVB in N-hTERT keratinocytes). **C:** Cleavage of PARP and protein abundance of p53 in TRIM29 shRNA cell line 3, in N-hTERT parental cells and in the Neg shRNA cell line were analysed by Western blotting. The full length PARP protein (116 kDa) and the fragment resulting from PARP cleavage (85 kDa) are indicated. N-hTERT cells exposed to a lethal dose of 2,400 mJ/cm^2^ of UVB were used as positive control. **D:** Phosphorylation of ATM in TRIM29 shRNA cell line 3, in N-hTERT parental cells and in the Neg shRNA cell line were analysed by Western blotting with nuclear extracts. Total ATM abundance was used to assess protein loading. **E–F:** The relative abundance of p21^WAF-1^ (E) and involucrin mRNA (F) was analysed by RT-PCR at 16 h after the exposure to UVB. The mRNA abundance in N-hTERT keratinocytes not exposed to UVB (CTL) was considered as the 100% reference. The results are expressed as means ± SD (N = 3). NS: non significant, * p<0.05, ** p<0.01 and *** p<0.001 vs N-hTERT CTL cells except that straight lines indicate statistical tests on the ratios obtained between cells exposed to UVB, for each cell condition (N-hTERT, Neg shRNA, TRIM29 shRNA).

The increase of TRIM29 abundance in keratinocytes exposed to UVB is a novel finding. Thus we undertook further experiments to characterize the role of this protein. We established polyclonal N-hTERT cell lines in which expression of TRIM29 was silenced by induced expression of specific short hairpin RNA (shRNA). A negative shRNA cell line (Neg shRNA) was also created as stable transfection control. Near to complete knocked down expression of TRIM29 protein was found in two TRIM29 shRNA cell lines (TRIM29 shRNA 3 and 3b) exposed to UVB or not (Western blot on Figure 4A). The abundance of TRIM29 mRNA was significantly decreased by 70% in the TRIM29 shRNA cell lines (p<0.001) while there was no significant difference in the Neg shRNA cell line. Hypothesizing that TRIM29 is involved in cell survival after UVB exposure, we exposed these lines of keratinocytes to a maximal sublethal single dose (1,200 mJ/cm^2^). As shown previously, significant dose-dependent cytotoxicity was detected above 1,800 mJ/cm^2^ in the N-hTERT keratinocytes after a single exposure to UVB [7]. The abundance of TRIM29 was respectively extremely, moderately and weakly decreased in cell line TRIM29 shRNA 3, 3b and 4 (Figure 4A). Respectively in these cell lines, the cell survival was maximally (p<0.01), moderately (p<0.05) or mildly (not significant) affected by a single exposure to 1,200 mJ/cm^2^ of UVB (Figure 4B) compared to the parental N-hTERT cells. The survival of the negative shRNA cell line (Neg shRNA) was unaffected (Figure 4B). Furthermore, poly (ADP ribose) polymerase (PARP) cleavage was found only in the TRIM29 shRNA cell line after UVB exposure (Figure 4C). N-hTERT keratinocytes exposed to UVB at a lethal dose (2,400 mJ/cm^2^) were used as positive control for PARP cleavage (Figure 4C).

It was proposed that TRIM29 acts in a signaling pathway induced upon ionizing radiation [19], [20]. Generally speaking, cell cycle arrest induced by DNA damage is regulated by ATM or ATR (ATM and Rad-3-related protein) in response to double strand breaks inducing agents such as ionizing radiation or to UV-induced damage, respectively [21]. UVB-induced photoproducts can lead to double-strand breaks [22] and ATM can be phosphorylated by ATR upon UV treatment [19], [20]. Phosphorylation of ATM was still observed in TRIM29 shRNA keratinocytes after exposure to a single dose of UVB at 1,200 mJ/cm^2^ (Figure 4D). Thus the modification of cell survival observed in absence of TRIM29 is not dependent on the phosphorylation state of ATM after exposure to UVB.

Downstream of ATM/ATR, active p53 plays a central role in the response of keratinocytes exposed to UVB by inducing a transient cell cycle arrest namely through the expression of p21^WAF-1^ cyclin-dependent kinase inhibitor [23]. Western blots showed that the abundance of p53 was similarly increased in N-hTERT, Neg shRNA and TRIM29 shRNA (clone 3) keratinocytes after an exposure to a single dose of UVB at 1,200 mJ/cm^2^ (Figure 4C). Expectedly, p21^WAF-1^ mRNA abundance was also similarly increased in all these keratinocyte lines (Figure 4E).

The ratio of abundance of involucrin mRNA in the UVB-treated cells/non-treated cells was significantly higher in TRIM29 shRNA cell line 3 exposed to UVB in comparison to N-hTERT and Neg shRNA cell lines (p<0.01 in TRIM29 shRNA cell lines versus N-hTERT, p<0.05 in TRIM29 shRNA cell lines versus Neg shRNA). The TRIM29 shRNA cell line had a higher baseline abundance of involucrin compared to the control cell lines (Figure 4F).

### Increased HSP27 and p38^MAPK^ phosphorylation after exposure to UVB in keratinocytes lacking TRIM29 and DNA damage

**Figure 5:**
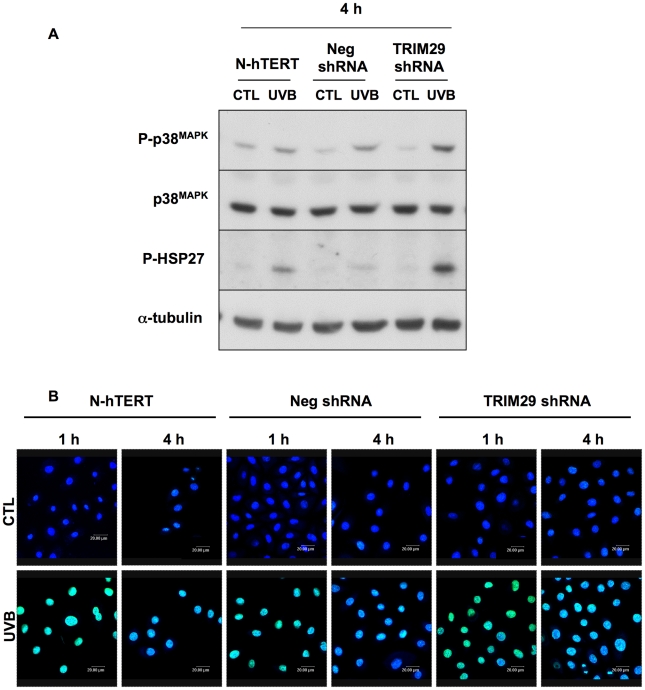
Effect of silencing of TRIM29 on the phosphorylation of HSP27 and p38^MAPK^ and on the level of DNA lesions after a single exposure to UVB. **A:** TRIM29 shRNA cell line (clone 3), N-hTERT parental cells and Neg shRNA cell line were exposed to a single exposure to UVB at 1,200 mJ/cm^2^. Abundance of HSP27 and p38^MAPK^ phosphorylations were analysed by Western blotting using specific anti-phospho-HSP27 or anti-phospho-p38^MAPK^ antibodies in cells exposed to UVB, or not (CTL), at 4h after the exposure. The membranes were stripped and reprobed with anti-HSP27 or p38^MAPK^ and anti-α-tubulin antibodies to assess protein loading. Results presented are representative of 3 independent experiments. **B:** Fluorescence micrographs of cyclobutane pyrimidine dimers (CPDs) immunostaining (green) obtained by semi quantitative confocal microscopy at 1 and 4 h after the exposure to UVB at 1,200 mJ/cm^2^ in TRIM29 shRNA cell line (clone 3), N-hTERT parental cells and Neg shRNA cell line. Control cells (CTL) were submitted to the same culture conditions without UVB. Nuclei were stained with TO-PRO-3 (blue).

In order to investigate whether cells lacking TRIM29 are more stress-sensitive, we checked the abundance of the phosphorylation of small Heat Shock Protein 27 (HSP27) and p38^MAPK^ after a single exposure to UVB. HSP27 is highly expressed in epidermis [24] and is induced upon stressful conditions like elevated temperatures, chemicals, reactive oxidants and by inflammatory cytokines [25]–[27] and plays a role in the molecular response of superficial keratinocytes to injurious events [25]. In primary keratinocytes exposed to UVB, HSP27 is phosphorylated by p38^MAPK^, triggering actin filament polymerization and maintenance of cytoskeleton integrity [13]. The p38^MAPK^ cell signaling is a key player in orchestrating UV-mediated stress responses in keratinocytes and skin (for a review, [28]). Western blots showed that the abundance of the phosphorylation of HSP27 and the p38^MAPK^ were more increased in TRIM29 shRNA (clone 3) keratinocytes than in N-hTERT and in Neg shRNA cells after an exposure to a single dose of UVB at 1,200 mJ/cm^2^ (Figure 5A). This suggests that this stress response is increased in keratinocytes lacking TRIM29.

Exposures to UVB induce DNA damage, predominantly cyclobutane pyrimidine dimers (CPDs) [2]. This DNA damage between adjacent pyrimidine bases can induce mutations in epidermal cells leading to the development of cancer cells [2]. At 1h after UVB exposure, CPDs were clearly observed by immunostaining in the nucleus of all the different cell lines (Figure 5B). But after 4h, more cells positive for CPDs staining were still observed in cells lacking TRIM29, explaining possibly cell death in this case.

### Other DNA damaging agents and PMA induce TRIM29

**Figure 6:**
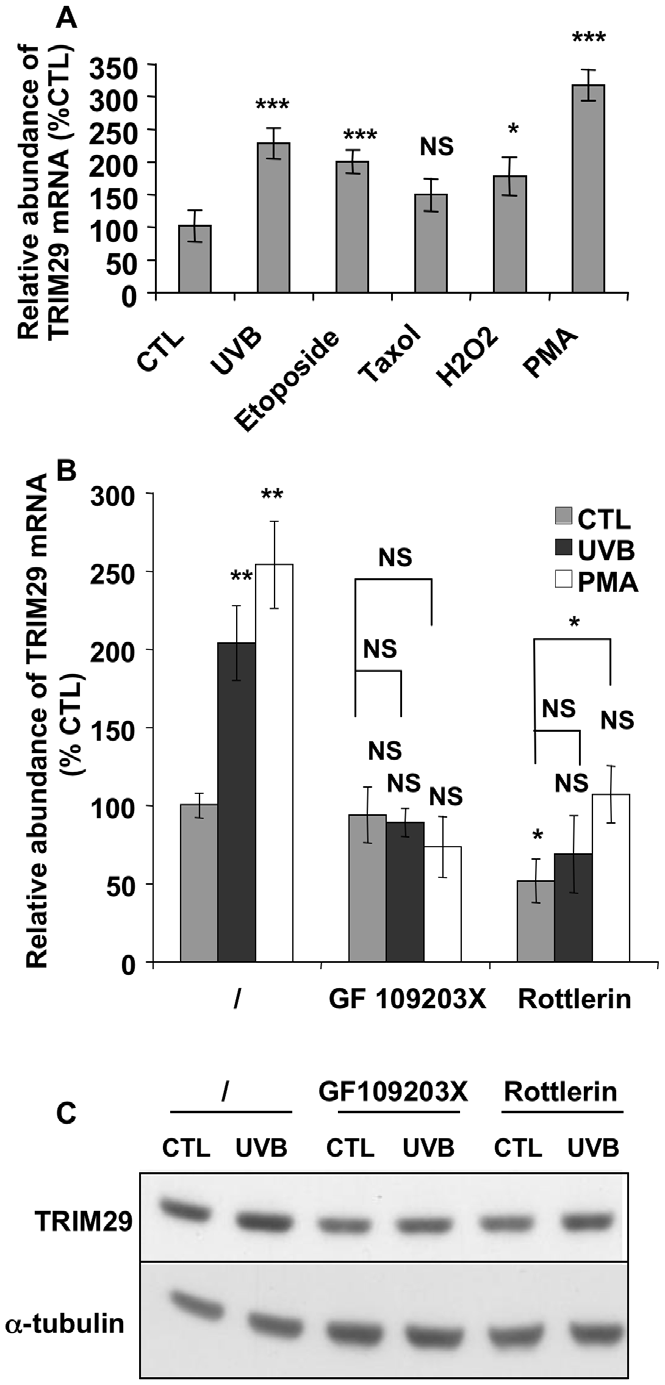
DNA damaging agents increase TRIM29 expression and TRIM29 expression is regulated by a PKC-dependent pathway. **A:** N-hTERT keratinocytes were incubated or not (CTL) with etoposide, with paclitaxel (taxol) for 16 h, with H_2_O_2_ for 25 min or with PMA for 15 min. N-hTERT cells exposed to UVB at 1,800 mJ/cm^2^ were used as positive control (UVB). RT-PCR for TRIM29 was performed 16 h later. The results (mean ± SD) are expressed as percentages compared with the mRNA abundance in control cells (N = 3, Student's *t*-test: NS: non significant, * p<0.05 and *** p<0.001 vs control cells). **B:** N-hTERT keratinocytes were preincubated with the PKC inhibitors GF109203X or rottlerin for 1 h. N-hTERT cells in medium alone were used as negative control (/). Then the cells were exposed or not to UVB at 1,200 mJ/cm^2^ (UVB) or were treated with PMA (PMA). Then cells were incubated for 16 h with the respective inhibitors of PKC. Control cells (CTL) were submitted to the same conditions without UVB or PMA treatment. RT-PCR was performed for TRIM29. The results are expressed as percentages increase compared to the mRNA abundance in cells without treatment (/, CTL). The results are given as mean ± SD (N = 4, NS: non significant, * p<0.05 and ** p<0.01 vs cells not exposed to UVB, PMA or inhibitors; except that straight line indicates vs CTL cells incubated with an inhibitor). **C:** The basal abundance of TRIM29 protein abundance is decreased by the PKC inhibitors rottlerin and GF109203X. N-hTERT keratinocytes were preincubated with the PKC inhibitors GF109203X or rottlerin for 1 h. N-hTERT cells incubated with medium alone were used as negative control (/). Then the cells were exposed or not to UVB (UVB) at 1,200 mJ/cm^2^ and were incubated for 16 h with the respective inhibitor. The protein abundance of TRIM29 was analysed by Western blotting. α-tubulin was used to assess protein loading.

We tested the effect of different DNA damaging agents on the abundance of TRIM29 mRNA. UVB at 1,800 mJ/cm^2^ was used as positive control. Etoposide induces apoptosis through DNA damage [29]. H_2_O_2_ generates oxidative DNA damage [30]. Treatments with UVB, etoposide and H_2_O_2_ induced an increase of the abundance of TRIM29 mRNA (Figure 6A). Taxol (paclitaxel) had no significant effect on the abundance of TRIM29. Taxol is not known to generate DNA damage. Its anti-mitotic and cytotoxic action is related to its ability to promote tubulin assembly into stable aggregated structures that cannot be polymerized [31].

### Protein kinase C controls TRIM29 expression

Phorbol-12-myristate-13-acetate (PMA) is a potent pharmacological activator of PKCs. PMA inhibits proliferation and induces the late differentiation markers involucrin and filaggrin [32]. Treatment with PMA increased the abundance of TRIM29 mRNA by 3.2-fold (Figure 6A). PKC isoforms regulate cell death and the balance between proliferation and differentiation of keratinocytes. There are three subgroups of PKCs: classical PKCs (α, βI, βII, γ), novel PKCs (δ, ε, η, θ), and atypical PKCs (ζ, ι/λ) [33]. The novel PKC isoforms expressed in keratinocytes (δ, ε, and η) [34] regulate differentiation by activating a p38δ Mitogen-Activated Protein Kinase (MAPK)-dependent cascade [35]. The PKC δ and η also modulate the apoptotic response of keratinocytes exposed to UVB [36], [37].

In order to evaluate whether TRIM29 gene regulation depended on a PKC, we tested the effect of chemical inhibitors of PKC on the abundance of TRIM29 mRNA after exposure to UVB or stimulation with PMA. GF109203X inhibits the conventional and novel isoforms of PKCs as competitor in their ATP binding site [38]. It blocked the induction of involucrin in our model (not shown). GF109203X prevented the UVB- and PMA-induced increase of abundance of TRIM29 mRNA (Figure 6B). PKCδ-specific inhibitor rottlerin decreased the basal abundance of TRIM29 mRNA by half and prevented the UVB-induced increase of TRIM29. The fold change compared to basal abundance in control cells remained significant after stimulation with PMA (Figure 6B). At the protein level, quantification of the Western blots (Figure 6C) showed that GF109203X and rottlerin decreased by half the basal level of TRIM29 (respectively by 52 and 44%). After exposure to UVB, no increase of the abundance of TRIM29 protein took place in the presence of one of these inhibitors, suggesting that PKCδ controls both the basal and induced level of TRIM29.

### Role of TRIM29 in survival of reconstructed epidermis exposed to UVB

**Figure 7:**
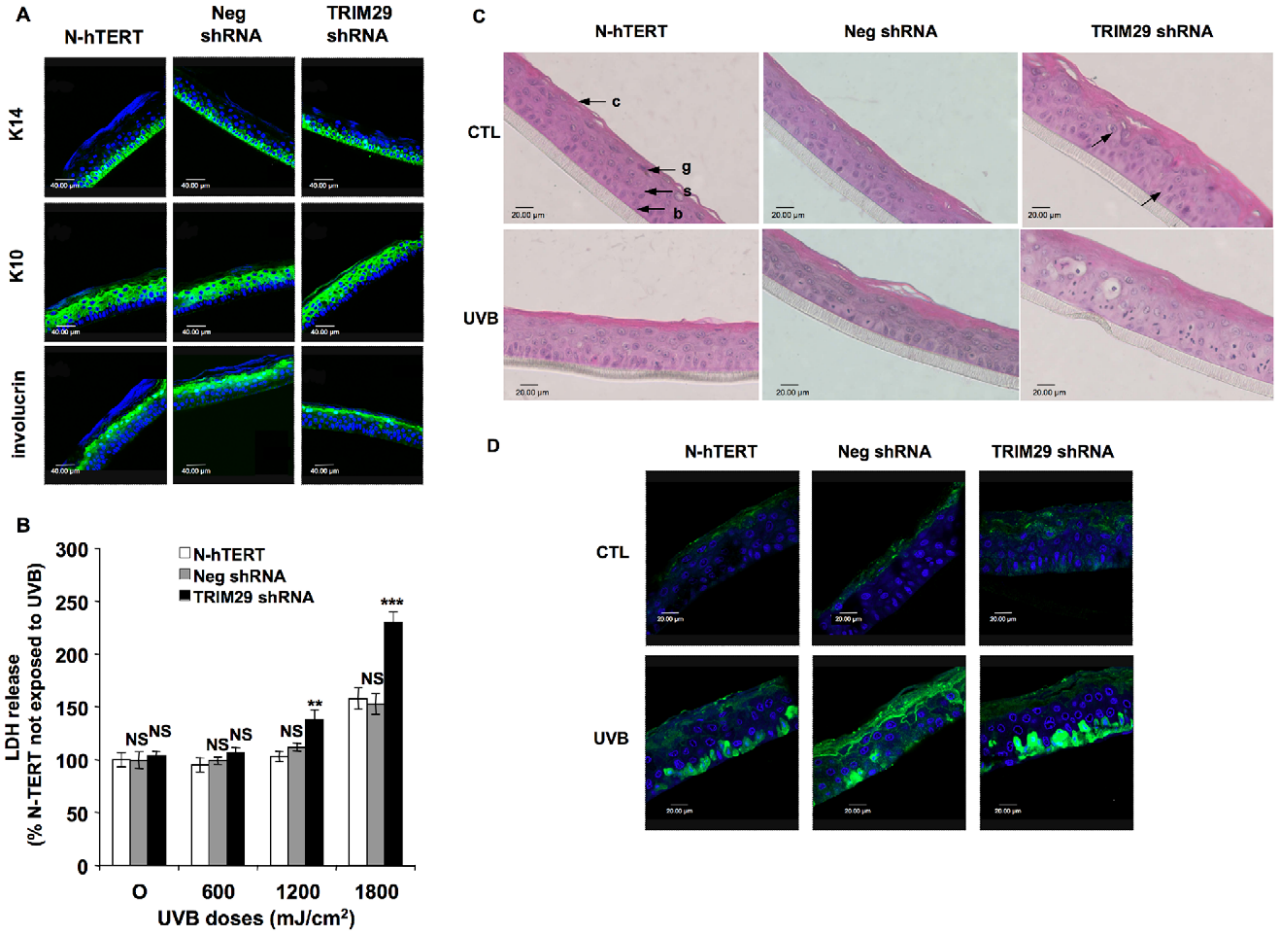
Effects of silencing of TRIM29 in reconstructed epidermis exposed to UVB. N-hTERT keratinocytes (N-hTERT) and transfectant cell lines expressing TRIM29 shRNA templates (TRIM29 shRNA) or the negative control shRNA (Neg shRNA) were used to reconstruct epidermises as explained in the material and methods section. **A:** Immunofluorescent detection of differentiation markers in histological sections from epidermises reconstructed with N-hTERT, Neg shRNA and TRIM29 shRNA cell lines. Primary antibodies labeling keratin 14 (K14), keratin 10 (K10) and involucrin were used (green). Nuclei were stained with TO-PRO-3 (blue). **B:** Effect of TRIM29 silencing on the UVB-induced cytotoxicity in reconstructed epidermis after exposure to UVB. N-hTERT, Neg shRNA and TRIM29 shRNA reconstructed epidermises were exposed to UVB at different doses. LDH release was measured 24 h later. The N-hTERT epidermis non-exposed to UVB (CTL) were considered as the 100% reference (NS: non significant, ** p<0.01, *** p<0.001 vs UVB in N-hTERT epidermis). The results are representative of three independent experiments. **C:** Effect of TRIM29 silencing on the histology of the reconstructed epidermises. N-hTERT, Neg shRNA and TRIM29 shRNA reconstructed epidermises were exposed to UVB at 1800 mJ/cm^2^. Sections perpendicular to the surface of reconstructed epidermises were obtained after fixation with formaldehyde, embedding in paraffin and staining with hematoxylin and eosin. The basal (b), spinous (s), granular (g) and cornified layers (c) are indicated (40× magnification). Picnotic cells are highlighted by arrows. Sunburn cells are detected in TRIM29 shRNA reconstructed epidermises exposed to UVB. **D:** Immunofluorescent detection of active-caspase 3 (green) in histological sections from epidermises reconstructed with N-hTERT, Neg shRNA and TRIM29 shRNA cell lines and exposed to UVB at 1800 mJ/cm^2^ or not (CTL). Nuclei were stained with TO-PRO-3 (blue).

To establish whether TRIM29 plays a role in the differentiation process of keratinocytes, the TRIM29 invalidated cell line (TRIM29 shRNA cell line 3), the N-hTERT and the Neg shRNA cell lines were grown at the air-liquid interface for 14 days in a model of reconstructed epidermis. We detected differentiation markers (K14, K10 and involucrin) specific to the state of differentiation of keratinocytes [1]. As expected, Keratin 14 was mainly localized in the basal layer, keratin 10 was abundant in the living suprabasal cell layers and involucrin was more abundant in the upper spinous and granular cell layers (Figure 7A). The reconstructed tissues were exposed to UVB at 600, 1,200 and 1,800 mJ/cm^2^. The cytotoxicity was assessed by monitoring total LDH release into the medium at 24 h after the exposure (Figure 7B). At 1,200 and 1,800 mJ/cm^2^, we observed a significantly increased cytotoxicity in the epidermis reconstructed with the TRIM29 shRNA cell line. The reconstructed epidermis showed normal histological features with a basal, spinous, granular and cornified layer without nucleus. However, we observed picnotic cells in the epidermis reconstructed with TRIM29 shRNA cell line 3, giving evidence of cell death during the differentiation process (Figure 7C). Moreover, after a single UVB stress at 1800 mJ/cm^2^, we can detect in the epidermis reconstructed with the TRIM29 shRNA cell line the presence of sunburn cells (Figure 7C), probably resulting from a higher stress-sensitivity of the cells lacking TRIM29. This was confirmed by an anti-active caspase 3 immunostaining in the same conditions (Figure 7D) where higher active-caspase 3 abundance was detected in the epidermis reconstructed with the TRIM29 shRNA cell line exposed to UVB.

## Discussion

Alternative differentiation is observed in human keratinocytes lacking p16^INK-4a^, expressing telomerase and retaining their differentiation capacities (N-hTERT cells) upon sublethal repeated exposures to UVB [7]. This UVB-induced alternative differentiation state is characterized by an increased abundance of involucrin, cytokeratins (K) K6, K16 and K17 [7], phosphorylation of p38^MAPK^ and HSP27, and elevated secretion of active metalloproteinase-9, as observed in primary keratinocytes and *in vivo* in the epidermis [4], [11], [12]. A 300 mJ/cm^2^ UVB dose may be considered as relevant. For instance, a single exposure of human skin grafts to 500 mJ/cm^2^ UVB induces sunburn cells in the suprabasal layers of the epidermal tissue, 24 h after the exposure [11]. A significant reduction in cell viability was observed after 8 repeated exposures to 400 mJ/cm^2^ UVB [7]. Immunodetection of active caspase-3 and analysis of cleavage of PARP as markers of apoptosis showed that 8 exposures to 300 mJ/cm^2^ UVB were not proapoptotic in contrast to keratinocytes exposed to a single 2,400 mJ/cm^2^ UVB dose. Thus keratinocytes responded differently after fractionated doses of UVB, as they survived to the treatment [7].

The proteomic profiling performed after the repeated exposures to UVB of N-hTERT keratinocytes identified proteins known to be involved in cell survival and differentiation (Table S1). These protein species include K6, K16, K17, PAI-2, 14-3-3σ and p-HSP27. CapG, isoforms of keratins namely P-S73 and P-S431 of K8 (results section of supplementary materials), and TRIM29 were identified in this context for the first time. Worth to discuss, CapG is an actin-capping protein involved in cell signaling, receptor-mediated membrane ruffling, phagocytosis and cell mobility [39]–[42]. Actin-binding proteins are prominent among the cytoskeletal proteins regulated by UV [6]. S73 phosphorylation of K8 is considered as a biomarker of stress response [43]. It contributes to the increase of solubility of K8, allowing a reorganization of keratin filaments [44]. Inability to phosphorylate K8 on S73 in transgenic mice models increases the susceptibility to apoptosis driven by Fas stimulation and to stress-induced liver injury [45]. Effects of phosphorylation of K8 on S73 may be extended to other keratins where this motif is found, such as K5 and K6 in keratinocytes [43]. Taken together, this suggests that this post-translational modification could promote the survival of keratinocytes after repeated exposures to UVB.

We showed that TRIM29 abundance is increased in N-hTERT and in primary keratinocytes after UVB exposure. A correlation was established between TRIM29 abundance and survival after exposure to UVB. Invalidation of TRIM29 expression allowed cell death to occur after exposure of reconstructed epidermis to UVB. H_2_O_2_ and etoposide increased TRIM29 mRNA abundance while taxol did not. In this model, repeated UVB exposures generate cyclobutane pyrimidine dimers in DNA rather than double strand breaks [7]. This suggests that the abundance of TRIM29 increases in other circumstances than case of double strand breaks. Interestingly, ATM was still phosphorylated, and the abundance of p53 protein and p21^WAF-1^ mRNA was still significantly increased upon exposure of TRIM29 shRNA cells to UVB, suggesting that the invalidation of TRIM29 did not impinge upon ATM and p53.

We observed that the abundance of the phosphorylation of HSP27 and p38^MAPK^ after a single exposure to UVB is higher in cells lacking TRIM29. Recently, Wang *et al.*, showed that TRIM29 activates the Β-catenin pathway in pancreatic cancer cells [46]. MAPK are key signaling pathways triggered by UV in epidermis [28]. Generally, the ERK pathway is linked to cell survival [47]. Activated p38^MAPK^ contributes to the activation of numerous transcription factors which promote events leading to cell cycle arrest, differentiation, apoptosis and inflammation in the afflicted tissues (for a review, [28], [48]).

The novel PKCδ and PKCη isoforms are associated with terminal differentiation in keratinocytes [34] and are key regulators of involucrin expression [35]. GF109203X blocked PMA and UVB-induced TRIM29 mRNA. As expected, rottlerin did not prevent the PMA-induced increase of TRIM29 mRNA since PMA is a general activator of many isoforms of PKCs. Rottlerin and GF109203X abolished the UVB-induced increase of TRIM29 mRNA and protein. These results suggest that the UVB-induced increase of TRIM29 mRNA and protein is regulated by PKCδ. As for basal levels, both inhibitors decreased the abundance of TRIM29 at protein level while only rottlerin decreased the level of TRIM29 mRNA. This effect is not clearly understood.

In addition to effects of PKCs on the expression of the gene, PKCs can phosphorylate the TRIM29 protein: PMA increases TRIM29 phosphorylation in human squamous carcinoma cells [20]. Phosphorylated isoforms could not be identified herein. However, preliminary 2D-DIGE data obtained already at 1 h after a single exposure to UVB showed that some spots identified as TRIM29 had increased intensity compared to the control cells, suggesting that a post-translational modification might occur. Thus, existing TRIM29 might be rapidly phosphorylated after stress and novel TRIM29 proteins could be synthesized, both through PKC-dependent processes.

Our experiments suggest that TRIM29 has a protective role. Consequently, it is important to note that the induction of TRIM29 may allow some altered cells to survive. In our previous study, we showed indeed that DNA damage persist in cells exposed repeatedly to UVB in contrast to a single exposure pointing the dangerous effects of cumulative exposures [7]. In such situation, increase of protein involved in cell survival, like TRIM29, may indeed potentially lead damaged cell to transformation. In this view, TRIM29 could be a protein involved in skin cancer. Indeed, while the abundance of TRIM29 is reduced in melanoma [49], in breast and in prostate cancer [50], [51], its abundance is induced and correlated with the severity of gastric cancer suggesting that function and role of TRIM29 in carcinogenesis may depend on the organ [52]. It would be interesting to compare the abundance of TRIM29 in transformed keratinocytes and in skin cancer.

In conclusion, one could propose that TRIM29 participates in the survival of differentiating keratinocytes, which are more and more exposed to UVB and pro-death stimuli. Interestingly, several picnotic cells were found in epidermis reconstructed with the TRIM29 shRNA cell line. This would be a novel mechanism allowing the survival of keratinocytes as they migrate and differentiate, in addition to other mechanisms which are already known [53].

Supplementary information is available at the journal's website.

## Materials and Methods

### Cell culture and treatments

#### Cultures in monolayers

Immortal human keratinocytes ectopically expressing the catalytic subunit of telomerase and characterized by non functional p16^INK-4A^ gene (N-hTERT keratinocytes) were a kind gift of P^r^ J. Rheinwald (Dpt of Medicine & Harvard Skin Disease Research Center, Boston MA, USA). N-hTERT keratinocytes were grown at 37°C under a 5% CO_2_ atmosphere in Epilife Medium containing human keratinocyte growth supplement HKGs (Cascade Biologics, Mansfield, UK). Cell culture conditions (no confluence) allowed control cells to remain proliferative, permitting the identification of differentially abundant proteins involved in growth arrest of the cells exposed to UVB. Primary keratinocytes were isolated by the trypsin float technique from normal adult human skin obtained during plastic surgery and were cultured similarly. Human hepatoma cells HepG2 were cultivated in DMEM (Invitrogen, Carlsbad, CA, USA) containing 10% (v:v) fetal bovine serum (Invitrogen, Carlsbad, CA, USA), under 5% CO_2_. Human dermal fibroblasts AG04431 (Coriell Institute for medical Research, Camden NJ, USA) were grown classically [54] in BME (Invitrogen, Carlsbad, CA, USA) containing 10% (v:v) fetal bovine serum (Invitrogen, Carlsbad, CA, USA) and 2 mM L-glutamine (Invitrogen, Carlsbad, CA, USA), under 5% CO_2_.

#### Reconstructed epidermis

Keratinocytes from N-hTERT, Neg shRNA or TRIM29 shRNA cell lines were seeded at a density of 5×10^5^ cells/cm^2^ on polycarbonate culture inserts (0.63 cm diameter) with 0.4 nm diameter pore size (Millipore, Brussels, Belgium) in 500 µl of EpiLife medium containing HKGs, KGF (10 ng/ml, R&D systems, Minneapolis, MN USA). CaCl_2_ was added to reach a final calcium concentration of 1.5 mM. The inserts were placed in six-well multiplates (Corning, Lowell, MA, USA) containing 1.4 ml of the same medium. One day later, the cells were exposed to the air-liquid interface by removing the culture medium in the upper compartment of the insert. Each insert was transferred in Petri culture dishes (Corning, Lowell, MA, USA) containing 14 ml of the EpiLife medium with HKGS, KGF (10 ng/ml) 1.5 mM calcium, and 50 g/ml vitamin C. Cells were incubated for another 13 days to obtain stratified cultures with the differentiation characteristics of a human epidermis.

#### Chemical treatments

N-hTERT keratinocytes were incubated for 16 h with 25 µM etoposide (Sigma-Aldrich, Bornem, Belgium) or with 5 µM paclitaxel (Molecular Probes, Leiden, The Netherlands), for 25 min with 500 µM H_2_O_2_ (Sigma-Aldrich, Munich, Germany) or for 15 min with 250 ng/ml PMA (Sigma-Aldrich, Munich, Germany), all diluted in Epilife medium. After treatment, fresh medium was provided for 16 h. N-hTERT keratinocytes were incubated with 5 µM rottlerin (Calbiochem, San Diego, CA, USA) or 10 µM GF109203X (Alexis Biochemicals, Farmingdale, NY, USA) from 1 h before exposure to UVB to 16 h after the exposure.

#### Exposure(s) to UVB

Single exposure of keratinocytes in monolayer to UVB: 200,000 keratinocytes were seeded in 25 cm^2^ flasks two days before exposure. The cells were washed with phosphate-buffered saline pH 7.4 (10 mM phosphate, 0.9% NaCl) (PBS), bathed in 3 ml of PBS and exposed to UVB at different doses between 300 and 2,400 mJ/cm^2^ using three TL 20W/01 lamps (Philips, Eindhoven, The Netherlands), emitting UVB peaking at 311 nm, and placed at 30 cm above the flasks. It is worth to note that this type of UVB lamp doesn't emit any UVC photons. The emitted radiation was checked under a flask lid using a UVR radiometer with UVB sensor (Bioblock Scientific, Tournai, Belgium).

Repeated exposures of keratinocytes in monolayer to UVB: keratinocytes were plated (2,000 cells/cm^2^) 64 h before the first exposure. The cells were washed with PBS and exposed to UVB at 300 mJ/cm^2^ in a thin layer of PBS. This exposure was repeated 4 times per day for 2 days. These conditions were shown previously to be sublethal [7]. After (each) exposure, fresh medium was provided. Control cells (CTL) were submitted to the same conditions without UVB.

Exposure of reconstructed epidermis: each insert of reconstructed epidermis was transferred in 1.2 ml of PBS in a 6-well plate and exposed to UVB at 600, 1,200 or 1,800 mJ/cm^2^. Afterwards the inserts were transferred to culture medium for 24 h. Control epidermis was submitted to the same conditions without UVB.

### Estimation of cell viability

In cell monolayers, cell viability was measured 16 hours after the last exposure to UVB, using the classical MTT method [55]. In the reconstructed epidermis, the release of lactate dehydrogenase (LDH) by the cells was assayed (Cytotoxicity detection kit, Roche, Indianapolis, IN, USA) according to the manufacturer's instructions.

### Protein extractions and Western blot analyses

The cells were lysed [30 mM Tris-HCl pH 8.5, 7 M urea, 2 M thiourea and 2% CHAPS (w/v)] and proteins analysed by SDS-PAGE and Western blotting as detailed in the materials and methods section of the supplementary materials. The following antibodies were used: mouse anti-PARP (BD Pharmingen, Erembodegem, Belgium), rabbit anti-TRIM29 (IMGENEX, San Diego, CA, USA), mouse anti-p53 (Upstate, Charlottesville, VA, USA), anti-phospho-ATM (Ser 1981, Cell Signaling, Leiden, The Netherlands), rabbit anti-phospho-HSP27 (Ser82) [56–489, Upstate USA], goat anti-HSP27 (C-20, Santa Cruz, CA, USA), mouse anti-phospho-p38^MAPK^ (Thr180/Tyr182), rabbit anti-p38^ MAPK^ (Cell Signaling, Leiden, The Netherlands) or mouse anti-α-tubulin (Sigma, Bornem, Belgium) as primary antibody. Secondary antibodies were anti-mouse, anti-rabbit and anti-goat horseradish peroxidase-linked (GE Healthcare, Uppsala, Sweden). α-Tubulin was used as loading control. Triplicates were performed.

### Immunofluorescence staining and confocal microscopy

Cells were fixed with 4% paraformaldehyde (Sigma, Bornem, Belgium) and permeabilized with 1% Triton X-100 (Sigma, Bornem, Belgium) before saturation with PBS containing 2% bovine serum albumin (BSA) (Sigma, Bornem, Belgium). For detection of cyclobutane pyrimidine dimers (CPDs), specific procedures were achieved according to the manufacturer's instructions (MBL, Woburn, MA, USA). Rabbit anti-TRIM29 antibody (IMGENEX, San Diego, CA, USA) was added before the specific Alexa Fluor 488 goat anti-rabbit IgG conjugates (Molecular Probes Leiden, The Netherlands). To visualize the nucleus, the cells were incubated with TO-PRO-3 (Molecular Probes). The coverslips were observed with a TCS confocal microscope (Leica, Solms, Germany) using a constant multiplier. Triplicates were performed.

### Histological and immunofluorescence analysis on reconstructed epidermis

The reconstructed tissues were fixed in 4% formaldehyde for 30 min, dehydrated by four incubations in methanol for 10 min, and incubated in toluene (four incubations for 10 min each) before embedding in paraffin. Tissue sections (6 µm thick) perpendicular to the filter were prepared for histological evaluation of stratified cultures with hematoxylin and eosin staining.

For immunofluorescent labeling, tissue sections were rehydrated, washed in PBS followed by 3 washes in PBS glycin (0.1 mM) and finally incubated in 1% BSA and 0.02% Triton X-100 in PBS for 30 min. Tissue sections were then incubated for 2 h with appropriate antibodies: mouse anti-involucrin antibody (Sigma, Bornem, Belgium), mouse anti-keratin 14 antibody (Novocastra, Zaventem, Belgium), mouse anti-keratin 10 antibody (Dako, Heverlee, Belgium). Secondary antibody incubation, TO-PRO-3 staining and observation by confocal microscopy were performed as described above.

Detection of active-caspase 3: tissue sections were rehydrated, washed in PBS followed by 3 washes in PBS glycin (0.1 mM), permeabilized in 0.1% Triton X-100 in PBS for 5 min and finally incubated in 0,5% BSA in PBS for 1 h. Tissue sections were then incubated overnight at 4°C with rabbit anti-active caspase 3 (Promega, USA). Secondary antibody incubation, TO-PRO-3 staining and observation by confocal microscopy were performed as described above.

Detection of TRIM29: human skin obtained from superficial normal adult skin from plastic surgery were used as positive control: frozen sections were fixed in 4% paraformaldehyde (Sigma, Bornem, Belgium), followed by 3 washes in PBS glycin (0.1 mM, Merck, Germany). For paraffin sections of reconstructed epidermis, antigen unmasking was performed by heat treatment at 95°C for 40 min in EDTA buffer (1 mM, pH 8.0, Merck, Germany) before saturation in PBS containing 1% BSA. TRIM29 immunofluorescence staining was performed classically.

### Real time RT-PCR

Total RNA was extracted (Total RNAgent extraction kit, Promega, Leiden, The Netherlands) and then reverse transcribed by the SuperScript II Reverse Transcriptase (Invitrogen, Carlsbad, CA, USA). Real time PCRs were performed using ABI PRISM 7900HT fast Real-Time PCR system (Applied Biosystems, Foster City, CA, USA). The sequences of the forward and reverse primers were respectively: 5′ –ctg gag act ctc agg gtc gaa- 3′ and 5′ –cca gga ctg cag gct tcc t- 3′ for p21^WAF-1^, 5′ –gtg gcc acc caa aca taa ata ac- 3′ and 5′ –cct agc gga ccc gaa ata agt- 3′ for involucrin, 5′- cta tgt gaa caa cta cac gaa cag -3′ and 5′– tgt cag gta cat gga gta tct ctt cat- 3′ for TRIM29, 5′ –acc cac tcc tcc acc ttt gac- 3′ and 5′ –gtc cac cac cct gtt gct gta- 3′ for GAPDH. Values are average of triplicates ± S.D.

### 2-D DIGE analysis

Protein extraction (at 16, 40 and 64 h after the last exposure of cells to UVB) and labeling with the cyanine dyes (GE Healthcare, Uppsala, Sweden) were performed as described in details in the Materials and methods section of the supplementary materials. 25 µg of each individual labeled samples (control, UVB-treated and pooled standard samples from triplicate experiments) were mixed and subjected to isoelectric focusing on a IPGphor isoelectric focusing unit (GE Healthcare, Uppsala, Sweden) along a continuous 4–7 pH gradient using IPG strips (18 cm, linear 4–7 pH, GE Healthcare, Uppsala, Sweden) with details in supplementary materials. Gels were scanned with a Typhoon 9400 imager (GE Healthcare, Uppsala, Sweden) to detect cyanin-labeled proteins. Determination of protein spot abundance and statistical analyses were carried out automatically using the DeCyder 2D Differential Analysis software 6.0 (GE Healthcare, Uppsala, Sweden). Protein spots with statistically significant Student's *t*-test (p<0.05) and more than 1.5-fold change in volume after normalization (between the two conditions in the triplicate) were considered as differentially abundant.

### Protein identification

A preparative gel was performed with 300 µg of unlabeled proteins (150 µg from each condition: UVB and control cells) and stained with Ruthenium II tris (bathophenanthroline disulfonate) (RuBPs) according to [57]. Each spot of interest was excised automatically using the Ettan spot picking system (GE Healthcare, Uppsala, Sweden). Detailed trypsin digestion and mass spectrometry procedures nanoLC-MS-MS (CapLC, Waters coupled on-line with a Q-tof2, Waters, Milford, MA, USA) are given in the Materials and methods section of the supplementary materials.

### TRIM29 and Neg shRNA cell lines generation

N-hTERT cell line, in which TRIM29 expression was silenced by a shRNA plasmid construct, were generated using the SureSilencing^™^ shRNA Plasmid product for human TRIM29 (SuperArray Bioscience Corporation, Leiden, The Netherlands) according to the manufacturer's instructions. Briefly, we used four plasmids containing four different shRNA templates directed against human TRIM29 sequence NM_012101 (termed clone 1, 2, 3 and 4) and cloned into the pGeneClip-Neo vector (Promega, Leiden, The Netherlands). The 21-nt target sequences are 5′-gcaggaatttggtgcattgat-3′; 5′-ccagaagaatttcaacaatct-3′; 5′-cggacaccatgaagagatact-3′ and 5′-aggacgacctgctcaatgtat-3′ respectively. Another plasmid contained the negative control shRNA (Neg shRNA; 5′-ggaatctcattcgatgccatac-3′), a scrambled artificial sequence that did not match any human, mouse or rat gene. Separate transformation and amplification into competent *E. coli* cells (*E. coli* Sure) was carried out. One day before transfection, 120,000 cells were seeded in a 6-wells plate without antibiotics. The five purified plasmids were transfected separately into N-hTERT cells using the FuGENE® 6 Transfection Reagent (Roche, Indianapolis, IN, USA) with 1 µg of each plasmid at a 6∶1 ratio of Transfection Reagent (µl) to DNA (µg), respectively. At 48 h post transfection, cells expressing the gene-specific shRNA were selected with 100 µg/ml of neomycin G418 (Invitrogen). After a polyclonal amplification, the extend of knock-down was tested by real-time RT-PCR and by Western blot analysis. Clone 3 yielded the best silencing of TRIM29 and was used in all the experiments (TRIM29 shRNA cell line) unless stated. This TRIM29 shRNA and the negative shRNA cell lines were maintained in culture with 100 µg/ml of neomycin G418 (Invitrogen) in Epilife medium.

### Statistical tests

Statistical analysis was carried out with the Student's *t*-test (NS: non significant, * p<0.05, ** p<0.01 and *** p<0.001 vs (respective) control.

## Supporting Information

Text S1 Supplementary manuscript including text, materials and methods and description of the functions of the most remarkable proteins identified in this study.
(0.49 MB DOC)

Figure S1 Increased abundance of CapG, several keratins and keratin phosphorylation after repeated exposures of N-hTERT keratinocytes to UVB. N-hTERT keratinocytes were exposed 8 times to UVB at 300 mJ/cm^2^. Control cells (CTL) were submitted to the same culture conditions without UVB. The results are representative of three independent experiments. A: Increased protein abundance of CapG in cells exposed to UVB. Western blot were carried out with samples of proteins obtained at 16, 40 and 64 h after the 8th exposure to UVB (UVB). A polyclonal antibody against CapG was used. α-tubulin protein level was used to assess loading. B: Localization and increased abundance of CapG after repeated exposures to UVB. CapG was detected by immunofluorescence at 16, 40 and 64 h after the 8th exposure to UVB. Micrographs of CapG immunofluorescence (green) were obtained by semi-quantitative confocal microscopy. Actin filaments were stained with fluorochrome-labeled phalloidin (red) and nuclei with TO-PRO-3 (blue). C: Increased of K6 abundance, K8 phosphorylations on serine residues (S73 and S431) without increased total K8 abundance at 16, 40 and 64 h after the 8th exposure to UVB. Phosphoserine-specific keratin antibodies for serine S73 or S431, K8 and K6 antibodies were used for a Western blot analysis. α-tubulin was used to assess protein loading.
(2.22 MB TIF)

Table S1 Proteins identified as differentially abundant in UVB-treated N-hTERT keratinocytes. Name of the identified proteins, Master number allocated by the DeCyder software at each time after the 8th exposure to UVB, and ratio of protein abundance between UVB-treated cells (UVB) and control cells (CTL) (a positive value means increased abundance of protein in UVB-exposed cells) are indicated. The corresponding p-value of the Student's t-test is given in parenthesis. The molecular weight (MW), the isoelectric point (pI), the accession number, the mascot score obtained from the identification by MS-MS, the number of peptides matched, the percentage of the sequence covering and the modifications observed are also mentioned (* proteins identified from a spot which contains a mix of several proteins; all the protein with significantly elevated score were taken into consideration). The list was sorted according to the main known function of the proteins.
(0.04 MB PDF)

Table S2 LC-MS-MS identification data including peptidic sequence of peptides. Proteins are listed according to their respective number in Table S1. S2A: Data on all the proteins identified in this study. S2B: Data on the four proteins for which only one peptide was identified (spot 740: dihydropyrimidinase-related protein 2; spot 418: Coronin-7; spot 1480: Glutathione S-transferase P (class-pi); and spot 1277: Nucleophosmin), on K16 for which only one peptide was specific in comparison with K17 and K14 (spot 986) and on Hsp27 found to be phosphorylated on S82 (spot 1302). S2C: MS spectrum of the four proteins for which only one peptide was identified (spot 740: dihydropyrimidinase-related protein 2; spot 418: Coronin-7; spot 1480: Glutathione S-transferase P (class-pi); and spot 1277: Nucleophosmin), of the only peptide of K16 which was specific in comparison with K17 and K14 (spot 986) and of the phosphorylation found on S82 of Hsp27 (spot 1302).
(1.01 MB PDF)

## References

[1] Eckert RL, Crish JF, Robinson NA (1997). The epidermal keratinocyte as a model for the study of gene regulation and cell differentiation.. Physiol Rev.

[2] Ichihashi M, Ueda M, Budiyanto A, Bito T, Oka M (2003). UV-induced skin damage.. Toxicology.

[3] Svobodova A, Walterova D, Vostalova J (2006). Ultraviolet light induced alteration to the skin.. Biomed Pap Med Fac Univ Palacky Olomouc Czech Repub.

[4] Rittie L, Fisher GJ (2002). UV-light-induced signal cascades and skin aging.. Ageing Res Rev.

[5] D'Errico M, Teson M, Calcagnile A, Proietti De Santis L, Nikaido O (2003). Apoptosis and efficient repair of DNA damage protect human keratinocytes against UVB.. Cell Death Differ.

[6] Li D, Turi TG, Schuck A, Freedberg IM, Khitrov G (2001). Rays and arrays: the transcriptional program in the response of human epidermal keratinocytes to UVB illumination.. Faseb J.

[7] Bertrand-Vallery V, Boilan E, Ninane N, Demazy C, Friguet B (2009). Repeated exposures to UVB induce differentiation rather than senescence of human keratinocytes lacking p16(INK-4A).. Biogerontology.

[8] Dickson MA, Hahn WC, Ino Y, Ronfard V, Wu JY (2000). Human keratinocytes that express hTERT and also bypass a p16(INK4a)-enforced mechanism that limits life span become immortal yet retain normal growth and differentiation characteristics.. Mol Cell Biol.

[9] Gorbunova V, Seluanov A, Pereira-Smith OM (2002). Expression of human telomerase (hTERT) does not prevent stress-induced senescence in normal human fibroblasts but protects the cells from stress-induced apoptosis and necrosis.. J Biol Chem.

[10] Lewis DA, Yi Q, Travers JB, Spandau DF (2008). UVB-induced Senescence in Human Keratinocytes Requires a Functional Insulin-like Growth Factor-1 Receptor and p53.. Mol Biol Cell.

[11] Del Bino S, Vioux C, Rossio-Pasquier P, Jomard A, Demarchez M (2004). Ultraviolet B induces hyperproliferation and modification of epidermal differentiation in normal human skin grafted on to nude mice.. Br J Dermatol.

[12] Sano T, Kume T, Fujimura T, Kawada H, Higuchi K (2009). Long-term alteration in the expression of keratins 6 and 16 in the epidermis of mice after chronic UVB exposure.. Arch Dermatol Res.

[13] Wong JW, Shi B, Farboud B, McClaren M, Shibamoto T (2000). Ultraviolet B-mediated phosphorylation of the small heat shock protein HSP27 in human keratinocytes.. J Invest Dermatol.

[14] Onoue S, Kobayashi T, Takemoto Y, Sasaki I, Shinkai H (2003). Induction of matrix metalloproteinase-9 secretion from human keratinocytes in culture by ultraviolet B irradiation.. J Dermatol Sci.

[15] Murnane JP, Kapp LN (1993). A critical look at the association of human genetic syndromes with sensitivity to ionizing radiation.. Semin Cancer Biol.

[16] McKinnon PJ (2004). ATM and ataxia telangiectasia.. EMBO Rep.

[17] Abraham RT (2001). Cell cycle checkpoint signaling through the ATM and ATR kinases.. Genes Dev.

[18] Kapp LN, Painter RB (1989). Stable radioresistance in ataxia-telangiectasia cells containing DNA from normal human cells.. Int J Radiat Biol.

[19] Brzoska PM, Chen H, Zhu Y, Levin NA, Disatnik MH (1995). The product of the ataxia-telangiectasia group D complementing gene, ATDC, interacts with a protein kinase C substrate and inhibitor.. Proc Natl Acad Sci U S A.

[20] Laderoute KR, Knapp AM, Green CJ, Sutherland RM, Kapp LN (1996). Expression of the ATDC (ataxia telangiectasia group D-complementing) gene in A431 human squamous carcinoma cells.. Int J Cancer.

[21] Batista LF, Kaina B, Meneghini R, Menck CF (2009). How DNA lesions are turned into powerful killing structures: Insights from UV-induced apoptosis.. Mutat Res.

[22] Han J, Colditz GA, Samson LD, Hunter DJ (2004). Polymorphisms in DNA double-strand break repair genes and skin cancer risk.. Cancer Res.

[23] Decraene D, Smaers K, Maes D, Matsui M, Declercq L (2005). A low UVB dose, with the potential to trigger a protective p53-dependent gene program, increases the resilience of keratinocytes against future UVB insults.. J Invest Dermatol.

[24] Huang CM, Foster KW, DeSilva T, Zhang J, Shi Z (2003). Comparative proteomic profiling of murine skin.. J Invest Dermatol.

[25] Boxman IL, Hensbergen PJ, Van Der Schors RC, Bruynzeel DP, Tensen CP (2002). Proteomic analysis of skin irritation reveals the induction of HSP27 by sodium lauryl sulphate in human skin.. Br J Dermatol.

[26] Huang CM, Xu H, Wang CC, Elmets CA (2005). Proteomic characterization of skin and epidermis in response to environmental agents.. Expert Rev Proteomics.

[27] Trautinger F (2001). Mechanisms of photodamage of the skin and its functional consequences for skin ageing.. Clin Exp Dermatol.

[28] Hildesheim J, Fornace AJ (2004). The dark side of light: the damaging effects of UV rays and the protective efforts of MAP kinase signaling in the epidermis.. DNA Repair (Amst).

[29] Karpinich NO, Tafani M, Rothman RJ, Russo MA, Farber JL (2002). The course of etoposide-induced apoptosis from damage to DNA and p53 activation to mitochondrial release of cytochrome c.. J Biol Chem.

[30] Armeni T, Battino M, Stronati A, Pugnaloni A, Tomassini G (2001). Total antioxidant capacity and nuclear DNA damage in keratinocytes after exposure to H2O2.. Biol Chem.

[31] Manfredi JJ, Horwitz SB (1984). Taxol: an antimitotic agent with a new mechanism of action.. Pharmacol Ther.

[32] Papp H, Czifra G, Lazar J, Gonczi M, Csernoch L (2003). Protein kinase C isozymes regulate proliferation and high cell density-mediated differentiation in HaCaT keratinocytes.. Exp Dermatol.

[33] Breitkreutz D, Braiman-Wiksman L, Daum N, Denning MF, Tennenbaum T (2007). Protein kinase C family: on the crossroads of cell signaling in skin and tumor epithelium.. J Cancer Res Clin Oncol.

[34] Denning MF (2004). Epidermal keratinocytes: regulation of multiple cell phenotypes by multiple protein kinase C isoforms.. Int J Biochem Cell Biol.

[35] Efimova T, Deucher A, Kuroki T, Ohba M, Eckert RL (2002). Novel protein kinase C isoforms regulate human keratinocyte differentiation by activating a p38 delta mitogen-activated protein kinase cascade that targets CCAAT/enhancer-binding protein alpha.. J Biol Chem.

[36] Matsumura M, Tanaka N, Kuroki T, Ichihashi M, Ohba M (2003). The eta isoform of protein kinase C inhibits UV-induced activation of caspase-3 in normal human keratinocytes.. Biochem Biophys Res Commun.

[37] D'Costa AM, Denning MF (2005). A caspase-resistant mutant of PKC-delta protects keratinocytes from UV-induced apoptosis.. Cell Death Differ.

[38] Toullec D, Pianetti P, Coste H, Bellevergue P, Grand-Perret T (1991). The bisindolylmaleimide GF 109203X is a potent and selective inhibitor of protein kinase C.. J Biol Chem.

[39] Sun HQ, Kwiatkowska K, Wooten DC, Yin HL (1995). Effects of CapG overexpression on agonist-induced motility and second messenger generation.. J Cell Biol.

[40] Witke W, Li W, Kwiatkowski DJ, Southwick FS (2001). Comparisons of CapG and gelsolin-null macrophages: demonstration of a unique role for CapG in receptor-mediated ruffling, phagocytosis, and vesicle rocketing.. J Cell Biol.

[41] Pellieux C, Desgeorges A, Pigeon CH, Chambaz C, Yin H (2003). Cap G, a gelsolin family protein modulating protective effects of unidirectional shear stress.. J Biol Chem.

[42] Silacci P, Mazzolai L, Gauci C, Stergiopulos N, Yin HL (2004). Gelsolin superfamily proteins: key regulators of cellular functions.. Cell Mol Life Sci.

[43] Toivola DM, Zhou Q, English LS, Omary MB (2002). Type II keratins are phosphorylated on a unique motif during stress and mitosis in tissues and cultured cells.. Mol Biol Cell.

[44] Ku NO, Azhar S, Omary MB (2002). Keratin 8 phosphorylation by p38 kinase regulates cellular keratin filament reorganization: modulation by a keratin 1-like disease causing mutation.. J Biol Chem.

[45] Ku NO, Omary MB (2006). A disease- and phosphorylation-related nonmechanical function for keratin 8.. J Cell Biol.

[46] Wang L, Heidt DG, Lee CJ, Yang H, Logsdon CD (2009). Oncogenic function of ATDC in pancreatic cancer through Wnt pathway activation and beta-catenin stabilization.. Cancer Cell.

[47] Martindale JL, Holbrook NJ (2002). Cellular response to oxidative stress: signaling for suicide and survival.. J Cell Physiol.

[48] Jinlian L, Yingbin Z, Chunbo W (2007). p38 MAPK in regulating cellular responses to ultraviolet radiation.. J Biomed Sci.

[49] Smith AP, Hoek K, Becker D (2005). Whole-genome expression profiling of the melanoma progression pathway reveals marked molecular differences between nevi/melanoma in situ and advanced-stage melanomas.. Cancer Biol Ther.

[50] Ernst T, Hergenhahn M, Kenzelmann M, Cohen CD, Ikinger U (2002). [Gene expression profiling in prostatic cancer].. Verh Dtsch Ges Pathol.

[51] Nacht M, Ferguson AT, Zhang W, Petroziello JM, Cook BP (1999). Combining serial analysis of gene expression and array technologies to identify genes differentially expressed in breast cancer.. Cancer Res.

[52] Kosaka Y, Inoue H, Ohmachi T, Yokoe T, Matsumoto T (2007). Tripartite motif-containing 29 (TRIM29) is a novel marker for lymph node metastasis in gastric cancer.. Ann Surg Oncol.

[53] Weisfelner ME, Gottlieb AB (2003). The role of apoptosis in human epidermal keratinocytes.. J Drugs Dermatol.

[54] Hayflick L, Moorhead PS (1961). The serial cultivation of human diploid cell strains.. Exp Cell Res.

[55] Mosmann T (1983). Rapid colorimetric assay for cellular growth and survival: application to proliferation and cytotoxicity assays.. J Immunol Methods.

[56] Bernerd F, Asselineau D (1997). Successive alteration and recovery of epidermal differentiation and morphogenesis after specific UVB-damages in skin reconstructed in vitro.. Dev Biol.

[57] Rabilloud T, Strub JM, Luche S, van Dorsselaer A, Lunardi J (2001). A comparison between Sypro Ruby and ruthenium II tris (bathophenanthroline disulfonate) as fluorescent stains for protein detection in gels.. Proteomics.

