# Supplementary material for: Proteomic Profiling of Human Keratinocytes Undergoing UVB-Induced Alternative Differentiation Reveals TRIpartite Motif Protein 29 as a Survival Factor
Source: PLoS One. 2010 May 3;5(5):e10462. doi: 10.1371/journal.pone.0010462 (PMC2862717; doi:10.1371/journal.pone.0010462)
Supplement: Text S1 — Supplementary manuscript including text, materials and methods and description of the functions of the most remarkable proteins identified in this study. (0.49 MB DOC) [file pone.0010462.s001.doc]

**SUPPLEMENTARY MATERIALS**

**MATERIALS AND METHODS (detailed procedures used in main part or procedures specifically used in this supplementary material section).**

***Western blots***

The proteins extracted were analyzed by SDS–polyacrylamide gel electrophoresis and transferred onto polyvinylidene difluoride (PVDF) membranes (GE Healthcare, Uppsala, Sweden). The membranes were blocked with PBS/Tween 20 (0.1%) containing 2% blocking agent (GE healthcare, Uppsala, Sweden), before incubating with specific primary antibodies. The antibodies used to obtain the results presented in this supplementary materials are : mouse anti-cytokeratin 8 (Novocastra, Zaventem, Belgium), mouse anti-phosphorylated K8 phospho-Ser73 specific (clone LJ4), or mouse anti-phosphorylated K8 phospho-Ser431 specific (clone 5B3) (Neomarkers, Fremont, CA, USA)*,* rabbit anti-CapG (a kind gift of P^r^ P. Silacci, Div. of Hypertension and vascular Medicine, Centre Hospitalier Universitaire Vaudois, Lausanne, Switzerland) or mouse anti-α-tubulin antibody (Sigma, Bornem, Belgium). After three washing steps, the membranes were incubated with the anti-mouse or anti-rabbit secondary antibody (horseradish peroxidase-linked secondaries antibodies (GE healthcare, Uppsala, Sweden). Finally, the bands were revealed with chemiluminescent substrates (ECL detection kit, GE healthcare, Uppsala, Sweden). α-tubulin was used as reference protein to check equal loading. Triplicates were performed.

***Immunofluorescence detection of CapG***

Cells were fixed with 4% paraformaldehyde (Sigma, Bornem, Belgium), permeabilized with triton X-100 1% (Sigma, Bornem, Belgium) before saturation in PBS containing 2% Bovine Serum Albumin (Sigma, Bornem, Belgium). The primary antibody against CapG (a kind gift of P^r^ P. Silacci, Div. of Hypertension and vascular Medicine, Centre Hospitalier Universitaire Vaudois, Lausanne, Switzerland) was used with an Alexa Fluor 546 phalloidin conjugate (Molecular Probes, Leiden, The Netherlands) following by the specific Alexa Fluor 488 goat anti-rabbit IgG conjugate antibody (Molecular Probes, Leiden, The Netherlands). To visualize the nucleus, cells were incubated with TO-PRO-3 (Molecular Probes, Leiden, The Netherlands). The coverslips were mounted in mowiol (Sigma) and observed with a TCS confocal microscope (Leica, Solms, Germany) using a constant multiplier.

***Sample preparation for 2-D DIGE analysis***

Cells from three independent experiments (at 16, 40 and 64 h after the last exposure of cells to UVB and respective controls at the same time) were rinsed with PBS, trypsinized, harvested and lysed in 100 μl of lysis buffer [30 mM Tris-HCl pH 8.5, 7 M urea, 2 M thiourea and 2% CHAPS (w/v)]. Samples were gently shacked at 8°C for 30 min at 1,200 rpm (Thermomixer Comfort, Eppendorf, Germany). Insoluble material was removed by centrifugation at 13,000 rpm (20 min at 4°C). Protein concentrations were determined in triplicates with Bio-Rad protein assay (BioRad, Germany) according to the Bradford method and adjusted between 5 to 10 μg/μl. The protein labeling with the cyanine dyes (GE Healthcare, Uppsala, Sweden) was performed according to the manufacturer’s instructions. Cy3 and Cy5 were used to label control (CTL) and UVB-treated (UVB) samples, while Cy2 was used to label a pooled standard. Briefly, 25 μg of proteins from either sample to be compared (CTL and UVB samples) were labeled with 200 pmol of dye (Cy3 or Cy5). To eliminate dye specific differences, samples from control cells (CTL) or cells exposed to UVB were labeled cross-wise with Cy3 and Cy5 for one of each triplicate. A pooled standard of 25 μg composed of equal amount of proteins from the 6 experimental conditions (controls and UVB treatments in triplicates) was labeled with 200 pmol of Cy2 that represented the internal standard on each gel. After 30 min on ice in the dark, the labeling was stopped by the addition of 10 mM lysine for 10 min. Cy3, Cy5 and Cy2 labeling was done identically with samples at 16, 40 and 64 h after the last exposure to UVB.

***Two-Dimensional In-Gel Electrophoresis***

25 μg of each individual labeled samples (control, UVB-treated and pooled standard samples from triplicate experiments) were combined, mixed with an equal volume of 2× sample buffer containing 7 M urea, 2 M thiourea, 2% CHAPS, 2% (w/v) DTT and 2% (v/v) IPG buffer pH 4-7 (GE Healthcare, Uppsala, Sweden) and then incubated at RT for 20 min to allow a better reduction before centrifugation for 10 min at 13,000 rpm. The mixed samples were subjected to isoelectric focusing on an IPGphor isoelectric focusing unit (Pharmacia Biotech, GE Healthcare, Uppsala, Sweden) along a continuous 4-7 pH gradient using IPG strips (18 cm, linear 4-7 pH, GE Healthcare). IPG strips were rehydrated overnight in 7 M urea, 2 M thiourea, 2 % CHAPS (w/v), 0.3 % DTT (w/v) and 0.5 % corresponding IPG buffer (v/v). Samples were loaded at the acidic end of the IPG strips by cup loading. IEF conditions were as follows : 300 V for 3 h, a gradient of 1,000 V for 8 h, followed by a gradient of 8,000 V for 3 h, and then 20,000 V / h (20°C) and a maximum current setting of 50 μA per strip. Prior to second-dimension separation, each IPG strip was equilibrated for 15 min in 10 ml of equilibration buffer [1.5 M Tris-HCl (pH 8.8), 6 M urea, 30% glycerol and 2% SDS] containing 10 mg/ml of DTT, followed by 15 min in 10 ml of equilibration buffer containing 25 mg/ml of iodoacetamide. The equilibrated strips were washed with SDS-PAGE running buffer (25 mM Tris-HCl pH 8.5, 192 mM glycine, and 0.1 % SDS), loaded on 10 % acrylamide gels, and migrated in electophoresis overnight at 14°C at 1 W per gel on a EttanDALT II system (GE Healthcare, Uppsala, Sweden).

***Gel scanning and image analysis***

Labeled proteins separated in the 2D gel were visualized using a Typhoon 9400 imager (GE Healthcare) at appropriate wavelength to detect the three cyanine dyes. The Cy2 images were scanned using 488 nm laser and an emission filter of 520 nm BP (band pass) 40. The Cy3 images were scanned using a 532 nm laser and an emission filter of 580 nm BP30. The Cy5 images were scanned using a 633 nm laser and a 670 nm BP30 emission filter. The resolution was 100 μm. Determination of protein spot abundance and statistical analyses were carried out automatically using the DeCyder 2D Differential Analysis software version 6.0 (GE Healthcare, Uppsala, Sweden). The three CyDye-labeled forms of each spot were co-detected within each gel. Ratios between sample (control cells versus UVB treatment) and internal standard abundances were calculated for each protein spot with the DIA (Differential In-gel Analysis) module. Inter-gel variability was corrected by matching of the internal standard spot maps by the BVA (Biological Variance Analysis) module of the DeCyder software that allows statistical analysis of protein abundance changes in different gels. Protein spots with statistically significant Student’s *t*-test (p< 0.05) and more than 1.5-fold change in volume after normalization (between the two conditions in the triplicate) were considered as differentially intense. Analysis was done for the samples collected at each time after the exposures to UVB.

***Preparative gels, staining, imaging, and spot excision***

As for protein identification and peptide sequencing, a preparative gel was performed with 300 μg of unlabeled proteins (150 μg from each condition : from cells exposed to UVB and control cells). These mixed proteins were loaded and separated in the same conditions as set up before. The preparative gels were stained with Ruthenium II tris for picking proteins of interest. The fluorescent probe ruthenium II tris (bathophenanthroline disulfonate) (RuBPs) was synthesized according to [1]. Briefly, 0.2 g of potassium pentachloro-aquo ruthenate (Alfa Aesar, Germany) was dissolved in 20 ml boiling water. The resulting solution was kept under reflux. After adding three molar equivalents of bathophenanthroline disulfonate (Alfa Aesar, Germany), the solution was kept under reflux for a further 20 min. 5 ml of a 500 mM sodium ascorbate solution were added to the mixture and refluxing was kept on for another 20 min. After cooling of the mixture to room temperature, pH was adjusted to 7.0 and distilled H_2_O was added to a total volume of 26 ml. Protein staining with fluorescent RuBPs was performed according to [1]. Briefly, gels were fixed 6 h in 30% ethanol, 10% acetic acid. Subsequently, 2D gels were rinsed in 20% ethanol for 3 × 30 min. Staining was carried out overnight in staining solution containing 10 μl of 20 mM RuBPs stock per liter 20% ethanol.

Prior to imaging, gels were equilibrated in water for 2 × 15 min. The gel was scanned using a 457 nm laser and a 610 nm BP30 emission filter (Typhoon 9400 imager, GE Healthcare, Uppsala, Sweden). Each spot of interest, defined based on CyDye images was matched with Ruthenium image and excised automatically using the Ettan spot picking system (GE Healthcare, Uppsala, Sweden). Following protein reduction 10 mM DTT in 100mM NH_4_HCO_3_ and alkylation 55 mM iodoacetamide in 100mM NH_4_HCO_3_, protein digestion was performed overnight at 37°C with trypsin Gold Mass (11 ng/μl, Promega, Leiden, The Netherlands) in 50mM NH_4_HCO_3_. The peptides were extracted in 5% formic acid for 20 min at 37°C.

***Protein identification and mass spectrometry***

Protein identification was performed using nanoLC-MS-MS (CapLC, Waters coupled on-line with a Q-tof2, Waters, Milford, MA, USA). Digested samples (20 μl) were loaded at a flow rate of 1 μl/min on a micro-precolumn cartridge (Symmetry 300 C18 5μm, Waters, USA). The precolumn was connected with the separating nanoscale C18 reverse-phase columns (Atlantis, 75μm × 150 mm, 3μm, NanoEase column, Waters, Milford, MA, USA). Peptides were eluted with a linear gradient (35 min) from 95% buffer A (H_2_O-acetonitrile-formic acid, 95/5/0.1 vol/vol) to 40% buffer B (acetonitrile-formic acid, 100/0.1 vol/vol), subjected to electrospray ionization and analyzed into a quadrupole-Tof Mass spectrometer (Waters, USA). The Q-Tof 2 was run in data direct analysis where a survey scan from 450 to 1200 m/z (mass-to-charge ratios) select the highest intensity peaks from doubly and triply charged ions. After collision-induced dissociation parent and daughter ions were detected from 50 to 1500 m/z. Data was acquired with massLynx 4.0 (Waters, USA). Mass data collected during LC-MS/MS analysis was processed and converted into a PKL file using the ProteinLynx Global Server software 2.2.5 (Waters) before searching for protein candidates in the human protein database (MSDB version of 200606, 3239079 sequences – Human) using the in-house Mascot 2.1 search software (Matrix Science, London, UK) allowing carbamidomethylation of cysteines as fixed modifications and oxidation of methionines as partial modification. For the other parameters, we use trypsin with one missed cleavage, peptide and MS/MS tolerance at 0.5 Da after calibration, all spectra underwent an internal mass correction using autodigested trypsin peak masses, m/z 211.10 Da. The mascot total score is derived from individual ions scores. Significant hits were inspected manually to eliminate false positives and individual peptides were selected based on criteria such as the individual ion score (cut off > 20) and the expected value (cut off < 1).

**RESULTS**

**2.A. Proteomic analysis of N-hTERT keratinocytes repeatedly exposed to UVB**

Most of the protein species underwent similar changes of abundance at 40 and 64 h after the last exposure to UVB, rather than at 16 h. The variations observed at 16 h might correspond to the immediate stress response in terms of cell cycle arrest and survival. The subsequent variations detected at 40 and 64 h could rather be effectors involved in the maintenance of the state of alternative differentiation. Among the spots selected in the proteomic analysis presented in the main part of this work, 69 proteins could be identified by LC-MS-MS. All the proteins identified were listed in Table S1. Full information about the LC-MS-MS identification data such as peptide sequences for each protein species is compiled in table S2A.

Also, data and mass spectrum on several proteins for which only one peptide was identified are shown in Table S2B and S2C.

Herein we described briefly the most remarkable of these proteins. They are sorted according to their main known function, namely in differentiation. Lastly, the increased abundance of CapG and the variations in the keratin phosphorylation pattern in N-hTERT keratinocytes exposed to repeated exposures to UVB were further characterized by western blot analyses.

**2.B. Description of the functions of the most remarkable proteins identified in this study.**

# *Proteins involved in the regulation of protein expression*

The down-regulation of Elongation Factor 2 observed in N-hTERT keratinocytes at 16h after repeated exposures to UVB is not surprising since UV have ribotoxic effects that inhibit translation [2]. In addition a transcriptomic analysis of primary keratinocytes after single exposure to UVB showed a rapid (< 4 h) but transient arrest of transcription.[3] RNA processing proteins were also identified as more abundant at 16 h such as N-ras upstream protein (NRU) which regulates mRNA turnover [4] or Leucine-rich PPR Motif-containing Protein which plays a role in translation or stability of mitochondrial mRNA [5,6]. Control of mRNA stability may be an important alternative to regulate the transcription after exposure to UV [7]. For example, UV can induce p21^WAF-1^ by stabilizing its mRNA in adult skin [8].

# *Proteins involved in the regulation of the cell cycle and in keratinocyte differentiation*

Several proteins involved in the cell cycle regulation were differentially abundant after the exposures to UVB. For instance proliferating cell nuclear antigen (PCNA) was down-regulated (- 1.59-fold at 64 h) favoring growth arrest [9-11]. Several proteins involved in keratinocyte differentiation were up-regulated like plasminogen activator inhibitor 2 or PAI-2 (2.3, 2.2 and 2.3-fold at 16, 40 and 64 h respectively) and adaptor protein 14-3-3σ (1.5-fold at 64 h). The serine protease inhibitor PAI-2 underwent the highest increase in protein abundance found in this study (2.3-fold). Furthermore, increased abundance of PAI-2 was found at 16, 40 and 64 h after the last exposure to UVB (Table S1). PAI-2 mRNA abundance increases in association with cornified envelope formation and cornification [12] after a single exposure of keratinocytes to UVB [3]. PAI-2 protein abundance increases in wound healing processes, inflammatory conditions and in cultured keratinocytes treated with irritant agents or pro-inflammatory cytokines like TNF-alpha [13-15]. PAI-2, also called Serpin B2 (serine proteinase inhibitor B2) protects against programmed cell death [16]. Interestingly, murine serine protease inhibitors are anti-apoptotic proteins [17]. Downregulation of the human Serpin B1 in reconstructed human epidermis at 24 h after exposure to irritant sodium lauryl sulphate potentially triggers cell death [18]. Consequently, upregulation of PAI-2 after a series of 8 sublethal exposures to UVB not only indicates an increased cornification but also a pro-inflammatory process. PAI-2 might also protect from exaggerate activation of serine proteases, thereby favoring their resistance to apoptosis.

Maspin is structurally homologous to the serine protease inhibitor superfamily and was more abundant in N-h-TERT keratinocytes exposed to UVB (1.6 and 1.7-fold at respectively 40 and 64 h). Maspin is a tumor suppressor up-regulated during keratinocyte senescence [19]. Therefore maspin could play a role in UVB-induced growth arrest.

The 14-3-3 family proteins regulate protein kinases and other proteins involved in the transduction of differentiation, proliferation and cell survival signals [20]. 14-3-3σ is an epithelial-specific marker found mainly in stratified squamous keratinizing epithelium, explaining it was originally called stratifin [21]. While 14-3-3σ expression is linked to keratinocyte differentiation, loss of its expression contributes to malignant transformation [22,23]. Following DNA-damage, 14-3-3σ is upregulated by a p53-dependent mechanism and blocks the cell cycle at G2/M, allowing repair of DNA damage [24,25]. Furthermore, 14-3-3σ can be associated *in vitro* and *in vivo* with cdk2 and cdk4, suggesting that it may also regulate G1/S progression [26]. Together with p21^WAF-1^ which mediates G1 arrest [27], 14-3-3σ could participate in growth arrest of N-hTERT keratinocytes after repeated exposures to UVB at sublethal dose. Cell cycle arrest and differentiation comply with the observed upregulation of p53, p21^WAF-1^ and involucrin [28].

# *Proteins involved in stress response*

The abundance of molecular chaperones was increased, including Hsp70 (1.5-fold at 40 h), Osmotic Stress Protein 94 (1.8 and 1.6-fold respectively at 16 and 64 h), phosphorylated Hsp27 (1.7-fold at 16 h) and protein disulfide-isomerase ER60 (1.7-fold at 16 h). These increased abundances indirectly suggest that protein misfolding and/or denaturation was generated in different subcellular compartments after the exposures to UVB. Increased amount of oxidized proteins were observed at 16 h in this model. Increased levels of oxidized and ubiquitinated proteins as well as proteins modified by the lipid peroxidation product 4-hydroxy-2-nonenal were observed in human keratinocytes exposed to 10 J/cm^2^ of UVA concomitantly with 0.05 J/cm^2^ of UVB [29]. Hsp27 is phosphorylated in human keratinocytes after a single exposure [30] and after repeated exposures to UVB. Increased abundance of chaperones protects against protein denaturation and may help protein refolding [31,32]. Furthermore Hsp70 and Hsp27 are anti-apoptotic [33,34]. In addition elevated Hsp can reduce the oxidative damage to proteins [31,32].

Glutathione S transferase was more abundant in N-h-TERT keratinocytes exposed to UVB (1.6-fold at 40 h). This enzyme can protect epithelial cells from oxidative stress [35]. The increased abundance of Tripartite motif-containing 29 (TRIM29) (1.8, 1.9 and 1.9-fold at 16 h) has been discussed in the main part of this article.

# *Structural proteins*

Alterations of the cytoskeleton accompany the response to UV in keratinocytes [36]. The greatest number of increases in protein abundance found in this proteome profiling is cytoskeleton-related proteins. The capping protein gelsolin like (CapG) and the cytokeratins K6, K8, K16, K17 are among these proteins and have been considered in the main part of this work. It is worth to note that Hsp27 is also an actin capping protein. Its phosphorylation is reported to stabilize actin microfilaments namely after exposure to UVB [30,37].

**2. C. CapG localizes at keratinocyte edges after repeated exposures to UVB.**

The balance between the unpolymerized monomeric actin pool and the filamentous actin polymer is highly regulated. In this respect, CapG abundance was 1.6, 1.7 and 1.6-fold increased at 16, 40 and 64 h after repeated exposures of N-hTERT keratinocytes to a sublethal dose of UVB. An increase in CapG abundance has been reported previously in endothelial cells exposed to shear stress [38], but had never been described in keratinocytes exposed to UVB. CapG is a ubiquitous gelsolin-related protein able to reversibly cap actin filaments [39]. Gelsolin-related proteins participate in the remodelling of the actin filament cytoskeleton [40,41]. Western blot analysis confirmed the increased abundance of CapG after repeated exposures to UVB at 16, 40 and 64 h after the last exposure (Figure S1A). No increase of CapG abundance was found in N-hTERT keratinocytes exposed to a single dose of 300 mJ/cm^2^ UVB (data not shown). Co-detection of CapG with actin-F was performed by immunohistochemistry and staining with rhodamine-labeled phalloidin (Figure S1B). Intense detection of CapG was observed with remarkable CapG localization at the cell edges after repeated exposures to UVB suggesting that CapG participates in the UVB-induced morphological changes observed in keratinocytes repeated exposed to UVB [28]. CapG is an actin-capping protein involved in cell signaling, receptor-mediated membrane ruffling, phagocytosis and cell mobility [38,42,43] by controlling actin filament polymerization and turnover, and affecting the metabolism of phosphoinositides [42,44]. Phosphoinositides generally favor the assembly and actin filament reticulation through recruitment of proteins, including capping proteins, at the plasma membrane [45]. A transient overexpression of CapG in fibroblasts leads to a decrease in the staining of actin filaments in the middle of the cell and not in cell periphery where the circumferential actin filaments remain prominent or even more pronounced. This results in disrupted actin filament organization [42]. Actin binding proteins are prominent among the cytoskeletal proteins regulated by UV [46]. In their transcriptional study of primary keratinocytes exposed to UVB, Li et al. showed that the overall picture of the regulation of cytoskeletal proteins by UVB begins with initial depolymerization, loosening of the actin cytoskeleton followed by a repolymerization of the filaments, and reconstitution of the cytoskeletal network. Actin polymerization, cross-link actin filaments to other proteins stabilizes and strengthens the microfilament cytoskeleton [46]. The increase in cell size observed after exposures to UVB [28] and modified actin microfilament dynamics driven by increased abundance of CapG could stabilize the cytoskeleton and could protect cells against oxidative stress-induced actin fragmentation [47].

**2.D. Keratins dynamics in response to repeated exposures to UVB of human keratinocytes.**

Cytokeratins compose intermediate filaments with a crucial role in the structural integrity of epithelial cells [48]. Different combinations of cytokeratins are expressed in epithelial cells according to cell type or differentiation state [49]. Epidermal injury involves transitory changes in their expression profile [50]. Beyond the primary protective function of keratins acting as a resilient scaffold, keratins dynamics play other roles in cell signaling, stress response to chemicals, apoptosis, as well as in pathophysiological mechanisms [49].

Keratins are poorly soluble and consequently poor extractable from biological samples. There is extensive overlap in their peptidic sequences making MS analysis complex [51,52]. Cytokeratins are frequently identified from spots that contain a mix of several cytokeratins [53,54]. Consequently keratins represent a challenge in proteomic studies [52].

The abundance of keratins K6, K16 and K17 was increased in keratinocytes repeatedly exposed to UVB confirming previous results obtained with western blots [28].

K6, K16, K17 were already known to be *de novo* synthesized after exposure of human skin to UVB [55]. K6 is induced in several skin diseases including abnormal differentiation [56,57] or hyperproliferative stratified epithelium such as in psoriasis [58]. K6 also participates in migration and wound healing processes [59,60]. K6, K16 and K17 expression coincides with alterations in activated keratinocytes and are interpreted as an alternative program of differentiation allowing for instance effective migration into the wound sites and sufficient resilience integrity in order to allow survival in the wound environment [50,59,61]. It was also shown in vivo that up-regulation of K16 leads to acanthosis and hyperkeratosis [62], suggesting that K16 may also act as a protective mechanism through epidermal thickening.

K17 is induced by interferon-γ (IFNγ) in keratinocytes [63], during skin inflammation linked with psoriasis [58], in skin allergy [63,64], in damaged epidermis [61] and in aged skin [65]. This suggests that K17 expression, together with PAI-2 [13], may be specific of inflammatory skin conditions that might be reproduced *in vitro* in our model after exposures to UVB.

K8 was identified from the 2D-DIGE profiling. K8 was identified in several differentially abundant spots located on the same isoelectric point, suggesting these were phosphorylated isoforms.

**LLS/TPL phosphorylation motif in K5/K6/K8 during resistance to stress**

K8 was already found in primary keratinocytes in vitro [66]. K8 can be phosphorylated during response of epithelial cells to injury [67]. Three sites of phosphorylation, on S23, S73 and S431, of human K8 have been identified *in vivo* [67]. We used two different antibodies available on the market that can bind to phospho-K8 and antibody able to bind total K8. From 16 to 64 h after 8 repeated exposures of N-hTERT keratinocytes to UVB, leading to alternative differentiation, K8 was phosphorylated on S73 (K8-pS73) and on S431 (Fig. S1C) while its abundance of K8 did not change (Figure S1C).

K8 was not phosphorylated on S73 or S431 after a single exposure to UVB at 300 mJ/cm^2^ (data not shown).

The phosphorylation of K8 on S73 belongs to a unique motif present in several type II keratins. Effects of phosphorylation of K8 on S73 may be extended to other keratins where this motif (LLS/TPL) is found, such as K5 and K6 in epidermal keratinocytes [68]. Cautious interpretation is needed. Indeed, in primary human cultured keratinocytes treated with UV-C, LLTPL motif of K5/6 becomes phosphorylated mainly at 24 h after the exposure and is recognized by the phosphospecific anti K8-pS73 antibody (clone LJ4). This type of phosphorylation is considered as a general biomarker of stress response [68].

This suggested it might be necessary to co-immunoprecipitate K8 and phospho-K8. This has been abandoned after several unsuccessful experiments

The phosphorylation of K8 on S73 is driven by p38^MAPK^ in stress conditions and contributes to increased solubility of K8, allowing a reorganization of keratin filaments [69]. It has been proposed that K8 phosphorylation on S73 is part of the anti-apoptotic response. Indeed, inability to phosphorylate K8 on S73 in transgenic mice models increases the susceptibility to apoptosis driven by Fas stimulation and to stress-induced liver injury. K8 P-S73 was proposed to represent a « phosphate sponge», thereby decreasing the phosphorylation of other proapoptotic substrates [70]. Taken together, the data about phosphorylation of K8 on S73 observed herein suggest that this post-translational modification might promote the survival of keratinocytes after repeated exposures to UVB.

**REFERENCES TO SUPPLEMENTARY MATERIAL**

1. Rabilloud T, Strub JM, Luche S, van Dorsselaer A, Lunardi J (2001) A comparison between Sypro Ruby and ruthenium II tris (bathophenanthroline disulfonate) as fluorescent stains for protein detection in gels. Proteomics 1: 699-704.

2. Iordanov MS, Pribnow D, Magun JL, Dinh TH, Pearson JA, et al. (1998) Ultraviolet radiation triggers the ribotoxic stress response in mammalian cells. J Biol Chem 273: 15794-15803.

3. Sesto A, Navarro M, Burslem F, Jorcano JL (2002) Analysis of the ultraviolet B response in primary human keratinocytes using oligonucleotide microarrays. Proc Natl Acad Sci U S A 99: 2965-2970.

4. Chang TC, Yamashita A, Chen CY, Yamashita Y, Zhu W, et al. (2004) UNR, a new partner of poly(A)-binding protein, plays a key role in translationally coupled mRNA turnover mediated by the c-fos major coding-region determinant. Genes Dev 18: 2010-2023.

5. Mili S, Pinol-Roma S (2003) LRP130, a pentatricopeptide motif protein with a noncanonical RNA-binding domain, is bound in vivo to mitochondrial and nuclear RNAs. Mol Cell Biol 23: 4972-4982.

6. Xu F, Morin C, Mitchell G, Ackerley C, Robinson BH (2004) The role of the LRPPRC (leucine-rich pentatricopeptide repeat cassette) gene in cytochrome oxidase assembly: mutation causes lowered levels of COX (cytochrome c oxidase) I and COX III mRNA. Biochem J 382: 331-336.

7. Mitchell P, Tollervey D (2000) mRNA stability in eukaryotes. Curr Opin Genet Dev 10: 193-198.

8. Gorospe M, Wang X, Holbrook NJ (1998) p53-dependent elevation of p21Waf1 expression by UV light is mediated through mRNA stabilization and involves a vanadate-sensitive regulatory system. Mol Cell Biol 18: 1400-1407.

9. Bravo R (1986) Synthesis of the nuclear protein cyclin (PCNA) and its relationship with DNA replication. Exp Cell Res 163: 287-293.

10. Travali S, Ku DH, Rizzo MG, Ottavio L, Baserga R, et al. (1989) Structure of the human gene for the proliferating cell nuclear antigen. J Biol Chem 264: 7466-7472.

11. Moore JO, Palep SR, Saladi RN, Gao D, Wang Y, et al. (2004) Effects of ultraviolet B exposure on the expression of proliferating cell nuclear antigen in murine skin. Photochem Photobiol 80: 587-595.

12. Jensen PJ, Wu Q, Janowitz P, Ando Y, Schechter NM (1995) Plasminogen activator inhibitor type 2: an intracellular keratinocyte differentiation product that is incorporated into the cornified envelope. Exp Cell Res 217: 65-71.

13. Chung NM, Marshall CM, Leyden JJ, Lavker RM, Jensen PJ, et al. (2001) Sodium dodecyl sulfate induces plasminogen activator inhibitor type 2 expression in epidermal keratinocytes in vivo and in vitro. J Invest Dermatol 117: 647-653.

14. Kruithof EK, Baker MS, Bunn CL (1995) Biological and clinical aspects of plasminogen activator inhibitor type 2. Blood 86: 4007-4024.

15. Wang Y, Jensen PJ (1998) Regulation of the level and glycosylation state of plasminogen activator inhibitor type 2 during human keratinocyte differentiation. Differentiation 63: 93-99.

16. Dickinson JL, Bates EJ, Ferrante A, Antalis TM (1995) Plasminogen activator inhibitor type 2 inhibits tumor necrosis factor alpha-induced apoptosis. Evidence for an alternate biological function. J Biol Chem 270: 27894-27904.

17. Rothbarth K, Kempf T, Juodka B, Glaser T, Stammer H, et al. (2001) Intracellular location and nuclear targeting of the Spi-1, Spi-2 and Spi-3 gene-derived serine protease inhibitors in non-secretory cells. Eur J Cell Biol 80: 341-348.

18. Fletcher ST, Basketter DA (2006) Proteomic analysis of the response of EpiDerm cultures to sodium lauryl sulphate. Toxicol In Vitro 20: 975-985.

19. Nickoloff BJ, Lingen MW, Chang BD, Shen M, Swift M, et al. (2004) Tumor suppressor maspin is up-regulated during keratinocyte senescence, exerting a paracrine antiangiogenic activity. Cancer Res 64: 2956-2961.

20. Mhawech P (2005) 14-3-3 proteins--an update. Cell Res 15: 228-236.

21. Leffers H, Madsen P, Rasmussen HH, Honore B, Andersen AH, et al. (1993) Molecular cloning and expression of the transformation sensitive epithelial marker stratifin. A member of a protein family that has been involved in the protein kinase C signalling pathway. J Mol Biol 231: 982-998.

22. Moreira JM, Gromov P, Celis JE (2004) Expression of the tumor suppressor protein 14-3-3 sigma is down-regulated in invasive transitional cell carcinomas of the urinary bladder undergoing epithelial-to-mesenchymal transition. Mol Cell Proteomics 3: 410-419.

23. Ferguson AT, Evron E, Umbricht CB, Pandita TK, Chan TA, et al. (2000) High frequency of hypermethylation at the 14-3-3 sigma locus leads to gene silencing in breast cancer. Proc Natl Acad Sci U S A 97: 6049-6054.

24. Hermeking H, Lengauer C, Polyak K, He TC, Zhang L, et al. (1997) 14-3-3 sigma is a p53-regulated inhibitor of G2/M progression. Mol Cell 1: 3-11.

25. Taylor WR, Stark GR (2001) Regulation of the G2/M transition by p53. Oncogene 20: 1803-1815.

26. Laronga C, Yang HY, Neal C, Lee MH (2000) Association of the cyclin-dependent kinases and 14-3-3 sigma negatively regulates cell cycle progression. J Biol Chem 275: 23106-23112.

27. Decraene D, Smaers K, Maes D, Matsui M, Declercq L, et al. (2005) A low UVB dose, with the potential to trigger a protective p53-dependent gene program, increases the resilience of keratinocytes against future UVB insults. J Invest Dermatol 125: 1026-1031.

28. Bertrand-Vallery V, Boilan E, Ninane N, Demazy C, Friguet B, et al. (2009) Repeated exposures to UVB induce differentiation rather than senescence of human keratinocytes lacking p16(INK-4A). Biogerontology.

29. Bulteau AL, Moreau M, Nizard C, Friguet B (2002) Impairment of proteasome function upon UVA- and UVB-irradiation of human keratinocytes. Free Radic Biol Med 32: 1157-1170.

30. Wong JW, Shi B, Farboud B, McClaren M, Shibamoto T, et al. (2000) Ultraviolet B-mediated phosphorylation of the small heat shock protein HSP27 in human keratinocytes. J Invest Dermatol 115: 427-434.

31. Arrigo AP (2001) Hsp27: novel regulator of intracellular redox state. IUBMB Life 52: 303-307.

32. Kampinga HH (2006) Chaperones in preventing protein denaturation in living cells and protecting against cellular stress. Handb Exp Pharmacol: 1-42.

33. Mosser DD, Caron AW, Bourget L, Meriin AB, Sherman MY, et al. (2000) The chaperone function of hsp70 is required for protection against stress-induced apoptosis. Mol Cell Biol 20: 7146-7159.

34. Paul C, Manero F, Gonin S, Kretz-Remy C, Virot S, et al. (2002) Hsp27 as a negative regulator of cytochrome C release. Mol Cell Biol 22: 816-834.

35. Hayes JD, Strange RC (1995) Potential contribution of the glutathione S-transferase supergene family to resistance to oxidative stress. Free Radic Res 22: 193-207.

36. Lee KM, Lee JG, Seo EY, Lee WH, Nam YH, et al. (2005) Analysis of genes responding to ultraviolet B irradiation of HaCaT keratinocytes using a cDNA microarray. Br J Dermatol 152: 52-59.

37. Guay J, Lambert H, Gingras-Breton G, Lavoie JN, Huot J, et al. (1997) Regulation of actin filament dynamics by p38 map kinase-mediated phosphorylation of heat shock protein 27. J Cell Sci 110 (Pt 3): 357-368.

38. Pellieux C, Desgeorges A, Pigeon CH, Chambaz C, Yin H, et al. (2003) Cap G, a gelsolin family protein modulating protective effects of unidirectional shear stress. J Biol Chem 278: 29136-29144.

39. Southwick FS, DiNubile MJ (1986) Rabbit alveolar macrophages contain a Ca2+-sensitive, 41,000-dalton protein which reversibly blocks the "barbed" ends of actin filaments but does not sever them. J Biol Chem 261: 14191-14195.

40. Silacci P, Mazzolai L, Gauci C, Stergiopulos N, Yin HL, et al. (2004) Gelsolin superfamily proteins: key regulators of cellular functions. Cell Mol Life Sci 61: 2614-2623.

41. Sun HQ, Yamamoto M, Mejillano M, Yin HL (1999) Gelsolin, a multifunctional actin regulatory protein. J Biol Chem 274: 33179-33182.

42. Sun HQ, Kwiatkowska K, Wooten DC, Yin HL (1995) Effects of CapG overexpression on agonist-induced motility and second messenger generation. J Cell Biol 129: 147-156.

43. Witke W, Li W, Kwiatkowski DJ, Southwick FS (2001) Comparisons of CapG and gelsolin-null macrophages: demonstration of a unique role for CapG in receptor-mediated ruffling, phagocytosis, and vesicle rocketing. J Cell Biol 154: 775-784.

44. Schafer DA, Cooper JA (1995) Control of actin assembly at filament ends. Annu Rev Cell Dev Biol 11: 497-518.

45. Kwiatkowski DJ (1999) Functions of gelsolin: motility, signaling, apoptosis, cancer. Curr Opin Cell Biol 11: 103-108.

46. Li D, Turi TG, Schuck A, Freedberg IM, Khitrov G, et al. (2001) Rays and arrays: the transcriptional program in the response of human epidermal keratinocytes to UVB illumination. Faseb J 15: 2533-2535.

47. Huot J, Houle F, Spitz DR, Landry J (1996) HSP27 phosphorylation-mediated resistance against actin fragmentation and cell death induced by oxidative stress. Cancer Res 56: 273-279.

48. Fuchs E, Weber K (1994) Intermediate filaments: structure, dynamics, function, and disease. Annu Rev Biochem 63: 345-382.

49. Coulombe PA, Omary MB (2002) 'Hard' and 'soft' principles defining the structure, function and regulation of keratin intermediate filaments. Curr Opin Cell Biol 14: 110-122.

50. Freedberg IM, Tomic-Canic M, Komine M, Blumenberg M (2001) Keratins and the keratinocyte activation cycle. J Invest Dermatol 116: 633-640.

51. Gorg A, Obermaier C, Boguth G, Harder A, Scheibe B, et al. (2000) The current state of two-dimensional electrophoresis with immobilized pH gradients. Electrophoresis 21: 1037-1053.

52. Plowman JE (2007) The proteomics of keratin proteins. J Chromatogr B Analyt Technol Biomed Life Sci 849: 181-189.

53. Lilley KS, Friedman DB (2004) All about DIGE: quantification technology for differential-display 2D-gel proteomics. Expert Rev Proteomics 1: 401-409.

54. Wu WW, Wang G, Baek SJ, Shen RF (2006) Comparative study of three proteomic quantitative methods, DIGE, cICAT, and iTRAQ, using 2D gel- or LC-MALDI TOF/TOF. J Proteome Res 5: 651-658.

55. Del Bino S, Vioux C, Rossio-Pasquier P, Jomard A, Demarchez M, et al. (2004) Ultraviolet B induces hyperproliferation and modification of epidermal differentiation in normal human skin grafted on to nude mice. Br J Dermatol 150: 658-667.

56. Stoler A, Kopan R, Duvic M, Fuchs E (1988) Use of monospecific antisera and cRNA probes to localize the major changes in keratin expression during normal and abnormal epidermal differentiation. J Cell Biol 107: 427-446.

57. Weiss RA, Eichner R, Sun TT (1984) Monoclonal antibody analysis of keratin expression in epidermal diseases: a 48- and 56-kdalton keratin as molecular markers for hyperproliferative keratinocytes. J Cell Biol 98: 1397-1406.

58. Carlen LM, Sanchez F, Bergman AC, Becker S, Hirschberg D, et al. (2005) Proteome analysis of skin distinguishes acute guttate from chronic plaque psoriasis. J Invest Dermatol 124: 63-69.

59. Wong P, Coulombe PA (2003) Loss of keratin 6 (K6) proteins reveals a function for intermediate filaments during wound repair. J Cell Biol 163: 327-337.

60. Paladini RD, Takahashi K, Bravo NS, Coulombe PA (1996) Onset of re-epithelialization after skin injury correlates with a reorganization of keratin filaments in wound edge keratinocytes: defining a potential role for keratin 16. J Cell Biol 132: 381-397.

61. Bousquet O, Coulombe PA (2002) Les kératines: un autre regard sur la biologie de la peau. Medecine/Sciences 18: 45-54.

62. Takahashi K, Folmer J, Coulombe PA (1994) Increased expression of keratin 16 causes anomalies in cytoarchitecture and keratinization in transgenic mouse skin. J Cell Biol 127: 505-520.

63. Jiang CK, Flanagan S, Ohtsuki M, Shuai K, Freedberg IM, et al. (1994) Disease-activated transcription factor: allergic reactions in human skin cause nuclear translocation of STAT-91 and induce synthesis of keratin K17. Mol Cell Biol 14: 4759-4769.

64. Komine M, Freedberg IM, Blumenberg M (1996) Regulation of epidermal expression of keratin K17 in inflammatory skin diseases. J Invest Dermatol 107: 569-575.

65. Gromov P, Skovgaard GL, Palsdottir H, Gromova I, Ostergaard M, et al. (2003) Protein profiling of the human epidermis from the elderly reveals up-regulation of a signature of interferon-gamma-induced polypeptides that includes manganese-superoxide dismutase and the p85beta subunit of phosphatidylinositol 3-kinase. Mol Cell Proteomics 2: 70-84.

66. Gazel A, Ramphal P, Rosdy M, De Wever B, Tornier C, et al. (2003) Transcriptional profiling of epidermal keratinocytes: comparison of genes expressed in skin, cultured keratinocytes, and reconstituted epidermis, using large DNA microarrays. J Invest Dermatol 121: 1459-1468.

67. Omary MB, Ku NO, Liao J, Price D (1998) Keratin modifications and solubility properties in epithelial cells and in vitro. Subcell Biochem 31: 105-140.

68. Toivola DM, Zhou Q, English LS, Omary MB (2002) Type II keratins are phosphorylated on a unique motif during stress and mitosis in tissues and cultured cells. Mol Biol Cell 13: 1857-1870.

69. Ku NO, Azhar S, Omary MB (2002) Keratin 8 phosphorylation by p38 kinase regulates cellular keratin filament reorganization: modulation by a keratin 1-like disease causing mutation. J Biol Chem 277: 10775-10782.

70. Ku NO, Omary MB (2006) A disease- and phosphorylation-related nonmechanical function for keratin 8. J Cell Biol 174: 115-125.

**FIGURE LEGENDS OF SUPPLEMENTARY DATA**

**Figure S1.** **Increased abundance of CapG, several keratins and keratin phosphorylation after repeated exposures of N-hTERT keratinocytes to UVB.** N-hTERT keratinocytes were exposed 8 times to UVB at 300 mJ/cm^2^. Control cells (CTL) were submitted to the same culture conditions without UVB. The results are representative of three independent experiments.

**A:** Increased protein abundance of CapG in cells exposed to UVB. Western blot were carried out with samples of proteins obtained at 16, 40 and 64 h after the 8^th^ exposure to UVB (UVB). A polyclonal antibody against CapG was used. α-tubulin protein level was used to assess loading.

**B:** Localization and increased abundance of CapG after repeated exposures to UVB. CapG was detected by immunofluorescence at 16, 40 and 64 h after the 8^th^ exposure to UVB. Micrographs of CapG immunofluorescence (green) were obtained by semi-quantitative confocal microscopy. Actin filaments were stained with fluorochrome-labeled phalloidin (red) and nuclei with TO-PRO-3 (blue).

**C:** Increased of K6 abundance, K8 phosphorylations on serine residues (S73 and S431) without increased total K8 abundance at 16, 40 and 64 h after the 8^th^ exposure to UVB. Phosphoserine-specific keratin antibodies for serine S73 or S431, K8 and K6 antibodies were used for a Western blot analysis. α-tubulin was used to assess protein loading.

**TABLE LEGENDS OF SUPPLEMENTARY MATERIALS**

**Table S1. Proteins identified as differentially abundant in UVB-treated N-hTERT keratinocytes.** Name of the identified proteins, Master number allocated by the DeCyder software at each time after the 8^th^ exposure to UVB, and ratio of protein abundance between UVB-treated cells (UVB) and control cells (CTL) (a positive value means increased abundance of protein in UVB-exposed cells) are indicated. The corresponding p-value of the Student’s *t*-test is given in parenthesis. The molecular weight (MW), the isoelectric point (pI), the accession number, the mascot score obtained from the identification by MS-MS, the number of peptides matched, the percentage of the sequence covering and the modifications observed are also mentioned (* proteins identified from a spot which contains a mix of several proteins; all the protein with significantly elevated score were taken into consideration). The list was sorted according to the main known function of the proteins.

**Table S2. LC-MS-MS identification data including peptidic sequence of peptides.** Proteins are listed according to their respective number in Table S1. **S2A:** Data on all the proteins identified in this study. **S2B:** Data on the four proteins for which only one peptide was identified (spot 740: dihydropyrimidinase-related protein 2; spot 418 : Coronin-7; spot 1480 : Glutathione S-transferase P (class-pi); and spot 1277 : Nucleophosmin), on K16 for which only one peptide was specific in comparison with K17 and K14 (spot 986) and on Hsp27 found to be phosphorylated on S82 (spot 1302). **S2C:** MS spectrum of the four proteins for which only one peptide was identified (spot 740 : dihydropyrimidinase-related protein 2; spot 418 : Coronin-7; spot 1480 : Glutathione S-transferase P (class-pi); and spot 1277 : Nucleophosmin), of the only peptide of K16 which was specific in comparison with K17 and K14 (spot 986) and of the phosphorylation found on S82 of Hsp27 (spot 1302).
