# Supplementary figures and images for: Proteomic Profiling of Human Keratinocytes Undergoing UVB-Induced Alternative Differentiation Reveals TRIpartite Motif Protein 29 as a Survival Factor
Source: PLoS One. 2010 May 3;5(5):e10462. doi: 10.1371/journal.pone.0010462 (PMC2862717; doi:10.1371/journal.pone.0010462)

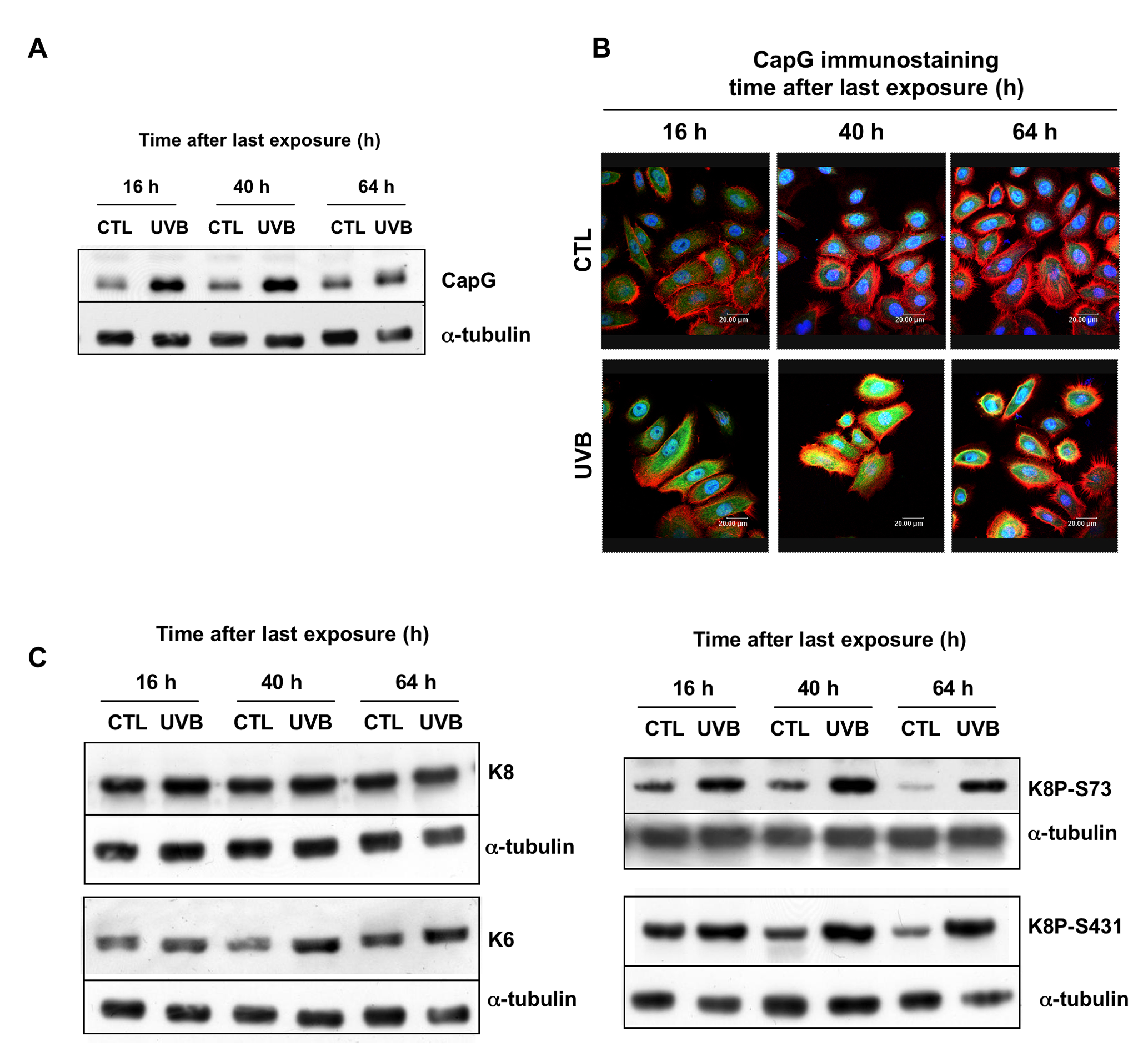

Supplement: Figure S1 — Increased abundance of CapG, several keratins and keratin phosphorylation after repeated exposures of N-hTERT keratinocytes to UVB. N-hTERT keratinocytes were exposed 8 times to UVB at 300 mJ/cm2. Control cells (CTL) were submitted to the same culture conditions without UVB. The results are representative of three independent experiments. A: Increased protein abundance of CapG in cells exposed to UVB. Western blot were carried out with samples of proteins obtained at 16, 40 and 64 h after the 8th exposure to UVB (UVB). A polyclonal antibody against CapG was used. α-tubulin protein level was used to assess loading. B: Localization and increased abundance of CapG after repeated exposures to UVB. CapG was detected by immunofluorescence at 16, 40 and 64 h after the 8th exposure to UVB. Micrographs of CapG immunofluorescence (green) were obtained by semi-quantitative confocal microscopy. Actin filaments were stained with fluorochrome-labeled phalloidin (red) and nuclei with TO-PRO-3 (blue). C: Increased of K6 abundance, K8 phosphorylations on serine residues (S73 and S431) without increased total K8 abundance at 16, 40 and 64 h after the 8th exposure to UVB. Phosphoserine-specific keratin antibodies for serine S73 or S431, K8 and K6 antibodies were used for a Western blot analysis. α-tubulin was used to assess protein loading. (2.22 MB TIF) [file pone.0010462.s002.tif]
