# Supplementary material for: Proteomic Profiling of Human Keratinocytes Undergoing UVB-Induced Alternative Differentiation Reveals TRIpartite Motif Protein 29 as a Survival Factor
Source: PLoS One. 2010 May 3;5(5):e10462. doi: 10.1371/journal.pone.0010462 (PMC2862717; doi:10.1371/journal.pone.0010462)
Supplement: Table S1 — Proteins identified as differentially abundant in UVB-treated N-hTERT keratinocytes. Name of the identified proteins, Master number allocated by the DeCyder software at each time after the 8th exposure to UVB, and ratio of protein abundance between UVB-treated cells (UVB) and control cells (CTL) (a positive value means increased abundance of protein in UVB-exposed cells) are indicated. The corresponding p-value of the Student's t-test is given in parenthesis. The molecular weight (MW), the isoelectric point (pI), the accession number, the mascot score obtained from the identification by MS-MS, the number of peptides matched, the percentage of the sequence covering and the modifications observed are also mentioned (* proteins identified from a spot which contains a mix of several proteins; all the protein with significantly elevated score were taken into consideration). The list was sorted according to the main known function of the proteins. (0.04 MB PDF) [file pone.0010462.s003.pdf]

Table S1. Proteins identified as differentially abundant in UVB-treated N-hTERT keratinocytes.

	Spot Number						ratio UVB/CTL (*t*-test)						
	16h	40h	64h	PM	pI	Protein identification	16h	40h	64h	accession number	Mascot score	Nb of peptides	seq cov %
	**Cell Cycle**												
1	360			81.8	6.1	minichromosome maintenance complex component 7	-1.7 (0.047)			AAH13375	381	9	14
2			1362	24.4	4.7	SFN protein = 14-3-3 protein sigma			1.51 (0.022)	P31947	361	9	39
3		1320		29	4.6	proliferating cell nuclear antigen			-1.59 (0.045)	AAH00491	273	9	34
	**Cytoskeleton, cytoskeletal associated protein and structural protein**												
4	528	725		62.7	6	dihydropyrimidinase-related protein 2 *	-2.73 (0.021)	-1.64 (0.039)		Q16555	67	3	4
5	518			62.7	6	dihydropyrimidinase-related protein 2	-1.75 (0.047)			Q16555	70	2	3
6	575			62.7	6	dihydropyrimidinase-related protein 2	-1.87 (0.037)			Q16555	167	4	7
7			740	62.7	6	dihydropyrimidinase-related protein 2			-1.69 (0.032)	Q16555	41	1	1
8	541			66	5.1	lamin B1 *	-1.75 (0.021)			P20700	961	26	37
9			693	66	5.1	lamin B1			-2.7 (0.038)	P20700	480	15	27
10			689	66	5.1	lamin B1			-2.31 (0.037)	P20700	490	15	25
11	1019	1180	1127	38.7	5.9	macrophage capping protein (CapG)	1.57 (0.021)	1.74 (0.018)	1.56 (0.037)	P40121	767	8	20
12	783			53.5	5.5	cytokeratin-8	1.55 (0.044)			P05787	1182	26	45
13	715			53-60	5.5-8	cytokeratin 8 and 6A *	1.69 (0.031)			P05787, AAH08807	286, 97	4, 5, 5	11, 9, 9
14	866			48	5	cytokeratin 17	1.65 (0.025)			Q04695	2702	28	52
15		1063		48-52	5-5.1	cytokeratin 17 and 14		1.81 (0.044)		Q04695, P02533	1278, 633	23, 15	45, 28
16	854			48-52	5-5.1	cytokeratin 17 and 14	1.6 (0.021)			Q04695, P02533	1487, 792	21, 12	39, 20
17		1054	1026	48-52	5-5.1	cytokeratin 17 and 14		2 (0.039)	1.66 (0.027)	Q04695, P02533	1195, 728	23, 14	43, 22
18			986	48-62	5-5.1	cytokeratin 17 and 14 and 16			1.58 (0.0091)	Q04695, P02533, P08779	751, 523, 223	19, 14, 10	40, 26, 17
19		1046		48-52	5-5.1	cytokeratin 17 and 14		1.69 (0.035)		Q04695 and P02533	932, 400	24, 13	47, 22
20	821			48-52	4.7-5	cytokeratin 14 and 17	1.71 (0.038)			P02533, Q04695	565, 147	14, 8	28, 17
21		1023		52	5.1	cytokeratin 14		1.53 (0.018)		P02533, P08779	2093	26	46
22		970	913	60-62	5.2-8	cytokeratin 5 and 6F *		1.77 (0.043)	1.61 (0.038)	P13647, P48669	316, 105	16, 7	25, 10
23			882	60	8	cytokeratin 6A *			1.52 (0.0091)	AAH08807	1259	23	44
24			870	60	8	cytokeratin 6A			1.54 (0.022)	AAH08807	662	19	34
25			862	60	8	cytokeratin 6A			1.58 (0.022)	AAH14152	427	15	29
26			871	60	8	cytokeratin 6A *			1.69 (0.022)	AAH08807	602	19	32
27		1525	1465	17	5.6	stathmin 1		-1.78 (0.02)	-2.39 (0.0091)	AAH82228	79	3	20
28		292		140	5.3	dynactin 1		1.56 (0.02)		AAD03694	343	12	9
29			418	102	5.5	coronin-7			1.52 (0.038)	P57737	56	1	1
	**Stress defense and DNA repair**												
30	1302			22.8	6	heat shock 27 kDa protein (Phospho S82)	1.69 (0.041)			P04792	375	6	24
31	239	442		95.4	5.6	osmotic stress protein 94	1.75 (0.039)	1.64 (0.013)		O95757	593	11	14
32	1319			26.8	5.4	glutathione S-transferase Mu 3	1.84 (0.03)			P21266	184	6	22
33		699		70	5.5	HSP70-2		1.54 (0.017)		AAD21815	159	6	7
34		1480		23.4	5.4	glutathione S-transferase P (class-pi)		1.55 (0.044)		P09211	55	1	4
35	539			66.5	6.7	tripartite motif-containing 29	1.83 (0.031)			AAH17352	366	12	22
36	525			66.5	6.7	tripartite motif-containing 29	1.92 (0.021)			AAH17352	304	11	20
37	532			66.5	6.7	tripartite motif-containing 29	1.94 (0.03)			AAH17352	325	13	23

Table S1

38		445		105	5.6	DNA mismatch repair protein		-1.82 (0.035)		I64819	115	4	3
	**Protease inhibitor**												
39	967			46.9	5.5	plasminogen activator inhibitor type 2 protein *	1.93 (0.021)			AAA36413	769	11	29
40		1118		44	5.5	plasminogen activator inhibitor type 2 protein		1.8 (0.013)		CAA01536	82	3	7
41	1001	1149	1092	46.8	5.5	plasminogen activator inhibitor 2 precursor	2.3 (0.039)	2.23 (0.021)	2.27 (0.033)	P05120	561	10	19
42		1145		44.2	5.5	plasminogen activator inhibitor type 2 protein *		2 (0.018)		CAA01536	245	4	12
43		1214	1159	42.5	5.7	maspin (protease inhibitor 5)		1.57 (0.013)	1.65 (0.022)	P36952	201	10	18
	**Metabolism**												
44		970	913	53	5.7	CNDP dipeptidase 2 *		1.77 (0.043)	1.61 (0.038)	AAH01375	84	3	7
45		1145		38.4	5.4	activator of 90 kDa heat shock protein ATPase homolog 1*		2 (0.018)		O95433	281	10	24
46			882	57.2	6.3	D-3-phosphoglycerate dehydrogenase*			1.52 (0.0091)	O43175	269	6	12
47			871	57.2	6.3	D-3-phosphoglycerate dehydrogenase *			1.69 (0.022)	O43175	188	6	14
	**Protein metabolism**												
48	648			57.5	4.8	protein disulfide-isomerase [Precursor]	2.02 (0.0087)			P07237	318	12	21
49	528			69	5.9	nuclear protein localization 4 homolog *	-2.73 (0.021)			AAH25930	163	7	10
50	715			57	6	protein disulfide-isomerase ER60 *	1.69 (0.031)			AAC51518	196	7	15
51		1235		32.7	4.7	nucleophosmin		-2.07 (0.028)		Q6V962	251	5	19
52		1277	1215	32.7	4.7	nucleophosmin		-1.7 (0.017)	-1.67 (0.024)	Q6V962	53	1	3
	**Metabolism - Energy pathways**												
53	502			81	7.2	glycerol-3-phosphate dehydrogenase, mitochondrial precursor	-1.56 (0.038)			P43304	308	13	17
54			876	57.8	5.2	3-hydroxy-3-methylglutaryl-Coenzyme A synthase 1			-1.87 (0.022)	AAH83514	264	8	15
55	1327	1457	1398	26.8	6.5	triosephosphate isomerase*	2.06 (0.03)	1.84 (0.013)	1.54 (0.043)	P60174	495	8	36
56	1327	1457	1398	28.3	6.3	ETHE1 protein *	2.06 (0.03)	1.84 (0.013)	1.54 (0.043)	O95571	53	2	12
57		1056	993	53	5.9	ubiquinol-cytochrome c reductase core protein I		-1.63 (0.017)	-1.6 (0.022)	AAH09586	296	8	15
	**Transcription regulatory protein, RNA binding protein, translation**												
58	266			96	6.4	elongation factor 2	-1.66 (0.041)			P13639	581	16	22
59	116			146	5.5	leucine-rich PPR motif-containing protein	-1.78 (0.042)			AAA67549	136	5	4
60	859			56.8	6.5	C-terminal binding protein 2	2.07 (0.047)			Q5SQP7	193	5	4
61	303			89.7	5.9	N-ras upstream protein NRU	-2.5 (0.013)			S29815	270	14	19
62	300			89.7	5.9	N-ras upstream protein NRU	-2.12 (0.021)			S29815	140	7	10
63	304			89.7	5.9	N-ras upstream protein NRU	-2.03 (0.037)			S29815	375	12	13
64			746	47.8	5.5	heterogeneous nuclear ribonucleoprotein K			-1.55 (0.043)	CAI16019	459	10	22
	**Miscellaneous**												
65	283			87.5	5.2	striatin 3 (Cell cycle autoantigen SG2NA)	1.53 (0.038)			Q13033	89	2	3
66		1240		26.8	4.7	hepatoma-derived growth factor		-1.8 (0.023)		A55055	217	4	16
67		805	769	56.8	5	EGF-containing fibulin-like extracellular matrix protein 1		-1.51 (0.024)	-1.6 (0.024)	AAH14410	254	6	15
68	541			70.8	5.2	L plastin*	-1.75 (0.021)			A35836	96	4	7
69	967			43	5.4	GA17 *	1.93 (0.021)			CAG29296	133	4	14
