# Supplementary material for: Proteomic Profiling of Human Keratinocytes Undergoing UVB-Induced Alternative Differentiation Reveals TRIpartite Motif Protein 29 as a Survival Factor
Source: PLoS One. 2010 May 3;5(5):e10462. doi: 10.1371/journal.pone.0010462 (PMC2862717; doi:10.1371/journal.pone.0010462)
Supplement: Table S2 — LC-MS-MS identification data including peptidic sequence of peptides. Proteins are listed according to their respective number in Table S1. S2A: Data on all the proteins identified in this study. S2B: Data on the four proteins for which only one peptide was identified (spot 740: dihydropyrimidinase-related protein 2; spot 418: Coronin-7; spot 1480: Glutathione S-transferase P (class-pi); and spot 1277: Nucleophosmin), on K16 for which only one peptide was specific in comparison with K17 and K14 (spot 986) and on Hsp27 found to be phosphorylated on S82 (spot 1302). S2C: MS spectrum of the four proteins for which only one peptide was identified (spot 740: dihydropyrimidinase-related protein 2; spot 418: Coronin-7; spot 1480: Glutathione S-transferase P (class-pi); and spot 1277: Nucleophosmin), of the only peptide of K16 which was specific in comparison with K17 and K14 (spot 986) and of the phosphorylation found on S82 of Hsp27 (spot 1302). (1.01 MB PDF) [file pone.0010462.s004.pdf]

Table S2A. LC-MS-MS identification data including peptidic sequence of peptides for all proteins identified.

N° in Table S1 N° spot protein identification

1 **360** **MCM7** minichromosome maintenance complex component 7

1. AAH13375 Mass: 81884 Score: 381 Coverage: 14% number of peptides : 9
BC013375 NID: - Homo sapiens replication licensing factor MCM7 - human
Check to include this hit in error tolerant search or archive report

Precursor mass **mass error observed** **peptide sequence**

Query	Observed	Mr(expt)	Mr(calc)	Delta	Miss	Score	Expect	Rank	Peptide
5	425.2719	848.5292	848.4504	0.0788	0	55	0.00084	1	K.YGNQLVR.L
13	515.3527	1028.6908	1028.5978	0.0930	0	40	0.024	1	K.AGILTTLNAR.C
22	594.3969	1186.7792	1186.6670	0.1123	0	72	1.6e-005	1	R.GSSGVGLTAAVLR.D
25	636.3489	1270.6832	1270.5717	0.1115	0	62	0.00012	1	R.QIAEEDFYEK.L
28	682.4042	1362.7938	1362.6714	0.1225	0	53	0,001	1	R.CSILAAANPAYGR.Y
30	683.3572	1364.6998	1364.5691	0.1307	0	81	1.8e-006	1	K.SEDDESGAGELTR.E
32	716.9060	1431.7974	1431.6704	0.1271	0	71	1.4e-005	1	R.QPPSQFEPLDMK.L + Oxidation (M)
34	725.4070	1448.7994	1448.6718	0.1277	0	46	0,0054	1	R.SPQNQYPAELMR.R + Oxidation (M)
39	574.9928	1721.9566	1721.8155	0.1411	0	24	0.74	1	K.MQEHSDQVPVGNIPR.S + Oxidation (M)

N° in Table S1 N° spot protein identification

2 **1362** **stratifin** 14-3-3 protein sigma

1. Q96DH0_HUMAN Mass: 24378 Score:361 Coverage: 39% number of peptides : 9
SFN protein.- Homo sapiens (Human).
Check to include this hit in error tolerant search or archive report

Query	Observed	Mr(expt)	Mr(calc)	Delta	Miss	Score	Expect	Rank	Peptide
8	452.2554	902.4962	902.5073	-0.0110	0	40	0.026	1	R.VLSSIEQK.S
9	454.2602	906.5058	906.5174	-0.0116	0	49	0.0037	1	R.NLLSVAYK.N
11	466.7119	931.4092	931.4221	-0.0129	1	36	0.061	1	K.MKGDYYR.Y
14	535.7637	1069.5128	1069.5226	-0.0098	0	40	0.024	1	K.EMPPTNPIR.L + Oxidation (M
15	554.7237	1107.4328	1107.4502	-0.0174	0	46	0.011	1	K.GEELSCEER.N
16	561.2314	1120.4482	1120.4569	-0.0086	0	43	0.012	1	R.YEDMAAFMK.G + Oxidation (M)
21	591.2830	1180.5514	1180.5611	-0.0097	0	75	6.2e-006	1	R.YLAEVATGDDK.K
25	629.7799	1257.5452	1257.5547	-0.0094	0	52	0,0014	1	R.SAYQEAMDISK.K + Oxidation (M)
31	516.2264	1545.6574	1545.6906	-0.0332	1	98	2.9e-008	1	K.SNEEGSEEKGPEVR.E

N° in Table S1 N° spot protein identification

3 **1320** **PCNA**
WMHUET Mass: 29092 Score: 273 Coverage: 34% number of peptides : 9
proliferating cell nuclear antigen - human
Check to include this hit in error tolerant search or archive report

Query	Observed	Mr(expt)	Mr(calc)	Delta	Miss	Score	Expect	Rank	Peptide
1	450.7358	899.4570	899.4535	0.0036	0	32	0.16	1	K.IADMGHLK.Y + Oxidation (M)
2	455.7072	909.3998	909.4014	-0.0016	0	50	0.0025	1	K.MPSGEFAR.I + Oxidation (M)
4	466.7490	931.4834	931.4803	0.0031	0	31	0,23	1	R.YLNFFTK.A
6	487.7135	973.4124	973.4141	-0.0016	0	47	0,0054	1	R.SEGFDTYR.C
7	493.3233	984.6320	984.6331	-0.0011	1	42	0,013	1	R.LVQGSILKK.V
9	631.3134	1260.6122	1260.6132	-0.0010	0	75	7e-006	1	K.CAGNEDIITLR.A
12	647.3226	1292.6306	1292.6360	-0.0054	0	84	7.7e-007	1	K.FSASGELGNGNIK.L
17	699.3422	1396.6698	1396.6690	0.0009	0	72	1.4e-005	1	R.NLAMGVNLTSMSK.I + 2 Oxidation (M)
19	757.3578	1512.7010	1512.6983	0.0027	1	27	0,38	1	K.YYLAPKIEDEEGS.-

N° in Table S1 N° spot protein identification

4 and 49 **528** **Nuclear protein localization 4 homolog and/or dihydropyrimidinase-related protein 2**

49 AAH25930 Mass: 69046 Score: 163 coverage: : 10% number of peptides : 7
Nuclear protein localization 4 homolog
BC025930 NID: - Homo sapiens
Check to include this hit in error tolerant search or archive report

Query	Observed	Mr(expt)	Mr(calc)	Delta	Miss	Score	Expect	Rank	Peptide
12	493.2974	984.5802	984.5352	0.0450	1	45	0,007	1	R.VQSPDGVKR.I
14	504.8145	1007.6144	1007.5651	0.0493	1	45	0,0069	1	R.ETAATFLKK.V
16	516.2776	1030.5406	1030.4978	0.0428	1	32	0,14	1	R.SRDPQLCR.H
20	522.7844	1043.5542	1043.5036	0.0507	0	41	0,021	1	R.LSPDGHFGSK.F
21	522.8054	1043.5962	1043.5498	0.0464	0	58	0,00038	1	K.AEVVDEIAAK.L
33	580.3257	1158.6368	1158.5815	0.0553	0	44	0,0086	1	K.CQPSAITLNR.Q
36	590.8409	1179.6672	1179.5957	0.0715	0	54	0,00088	1	K.FVALENISCK.I

4 JC5317 Mass: 62711 Score: 67 coverage: 4% number of peptides : 3
dihydropyrimidinase-related protein 2 - human
Check to include this hit in error tolerant search or archive report

Query	Observed	Mr(expt)	Mr(calc)	Delta	Miss	Score	Expect	Rank	Peptide
10	454.7752	907.5358	907.4915	0.0443	0	24	0,92	1	K.VFNLYPR.K
17	516.3001	1030.5856	1030.5407	0.0450	0	38	0,037	1	K.SSAEVIAQAR.K
25	542.8483	1083.6820	1083.6288	0.0533	0	58	0,00033	1	R.GSPLVVISQGK.I

N° in Table S1	N° spot	protein identification
5	518	**dihydropyrimidinase-related protein 2**

JC5317 Mass: 62711 Score: 70 Coverage: 3% number of peptides : 2
dihydropyrimidinase-related protein 2 - human
Check to include this hit in error tolerant search or archive report

Query	Observed	Mr(expt)	Mr(calc)	Delta	Miss	Score	Expect	Rank	Peptide
7	516.2861	1030.5576	1030.5407	0.0170	0	40	0,025	1	K.SSAEVIAQAR.K
11	542.8360	1083.6574	1083.6288	0.0287	0	60	0,00024	1	R.GSPLVVISQGK.I

N° in Table S1	N° spot	protein identification
6	575	**dihydropyrimidinase-related protein 2**

JC5317 Mass: 62711 Score: 167 Coverage: 7% number of peptides : 4
dihydropyrimidinase-related protein 2 - human
Check to include this hit in error tolerant search or archive report

Query	Observed	Mr(expt)	Mr(calc)	Delta	Miss	Score	Expect	Rank	Peptide
6	454.7765	907.5384	907.4915	0.0469	0	29	0,26	1	K.VFNLYPR.K
8	516.2947	1030.5748	1030.5407	0.0342	0	47	0,0051	1	K.SSAEVIAQAR.K
12	542.8485	1083.6824	1083.6288	0.0537	0	56	0,00053	1	R.GSPLVVISQGK.I
22	871.4625	1740.9104	1740.7988	0.1116	0	74	6.1e-006	1	K.MDENQFVAVTSTNAAK.V + Oxidation (M)

N° in Table S1	N° spot	protein identification
7	740	**dihydropyrimidinase-related protein 2**

2\. JC5317 Mass: 62711 Score: 41 Coverage: 1% number of peptides : 1
dihydropyrimidinase-related protein 2 - human
Check to include this hit in error tolerant search or archive report

Query	Observed	Mr(expt)	Mr(calc)	Delta	Miss	Score	Expect	Rank	Peptide
3	516.3078	1030.6010	1030.5407	0.0604	0	41	0,019	1	K.SSAEVIAQAR.K

N° in Table S1	N° spot	protein identification
8 and 68	541	**lamin B1 and/or L-plastin**

1\. VEHULB Mass: 66653 Score: 961 Coverage: 37% number of peptides : 26
8 lamin B1 - human
Check to include this hit in error tolerant search or archive report

Query	Observed	Mr(expt)	Mr(calc)	Delta	Miss	Score	Expect	Rank	Peptide
4	415.7192	829.4238	829.4294	-0.0055	0	34	0,15	1	K.LSPSPSSR.V
9	437.7292	873.4438	873.4556	-0.0117	0	43	0,014	1	R.LVEVDSGR.Q
14	486.7313	971.4480	971.4600	-0.0119	0	25	0,97	1	R.QIEYEYK.L
17	487.7354	973.4562	973.4716	-0.0153	0	54	0,0012	1	K.LLEGEEER.L
18	500.3123	998.6100	998.6124	-0.0023	1	57	0,00044	1	R.AKLQIELGK.C
19	512.7423	1023.4700	1023.4872	-0.0172	0	24	0,87	1	R.EYEAALNSK.D
20	515.7890	1029.5634	1029.5706	-0.0072	0	50	0,0025	1	K.QLADETLLK.V
22	522.7815	1043.5484	1043.5611	-0.0126	1	48	0,0044	1	R.LQEKEELR.E
24	523.7651	1045.5156	1045.5152	0.0004	1	45	0,0091	1	R.ALDDTARER.A
25	534.7801	1067.5456	1067.5546	-0.0089	0	55	0,00081	1	K.LAQALHEMR.E
26	536.3041	1070.5936	1070.6084	-0.0147	1	42	0,016	1	R.LKLSPSPSSR.V
27	537.7655	1073.5164	1073.5353	-0.0188	0	45	0,0079	1	K.ESDLNGAQIK.L
29	538.8239	1075.6332	1075.6389	-0.0057	1	51	0,002	1	R.LAVYIDKVR.S
31	549.2880	1096.5614	1096.5764	-0.0150	1	68	3e-005	1	R.KIGDTSVSYK.Y
35	577.8056	1153.5966	1153.6091	-0.0125	0	68	4.1e-005	1	R.AGGPTTPLSPTR.L
36	579.8349	1157.6552	1157.6656	-0.0103	1	65	8.4e-005	1	K.KQLADETLLK.V
37	586.3300	1170.6454	1170.6496	-0.0041	0	70	4.9e-005	1	R.IQELEDLLAK.E
41	587.3264	1172.6382	1172.6401	-0.0018	1	91	2.3e-007	1	K.DAALATALGDKK.S
44	608.3276	1214.6406	1214.6506	-0.0100	1	55	0,00067	1	K.LLEGEEERLK.L
48	626.3125	1250.6104	1250.6142	-0.0038	0	54	0,00076	1	K.ALYETELADAR.R
51	647.3407	1292.6668	1292.6724	-0.0056	1	67	4.2e-005	1	K.LREYEAALNSK.D
52	667.7970	1333.5794	1333.5898	-0.0104	0	51	0,0021	1	K.NQNSWGTGEDVK.V
54	700.8018	1399.5890	1399.5925	-0.0035	0	39	0,028	1	K.SMYEEEINETR.R
57	756.8514	1511.6882	1511.6886	-0.0003	0	114	7.8e-010	1	R.LSSEMNTSTVNSAR.E + Oxidation (M)
61	551.2751	1650.8035	1650.8253	-0.0218	1	62	0,00011	1	R.LYKEELEQTYHAK.L
63	512.7450	2046.9509	2047.0017	-0.0508	1	60	0,00014	1	K.LAQALHEMREQHDAQVR.L + Oxidation (M)

A35836 Mass: 70815 Score: 96 Coverage: 7% number of peptides : 4
68 L-plastin - human
Check to include this hit in error tolerant search or archive report

Query	Observed	Mr(expt)	Mr(calc)	Delta	Miss	Score	Expect	Rank	Peptide
33	559.2908	1116.5670	1116.5750	-0.0079	0	29	0,31	1	K.AACLPLPGYR.V
34	568.3066	1134.5986	1134.6033	-0.0046	0	61	0,00021	1	R.QFVTATDVVR.G
47	626.2692	1250.5238	1250.5383	-0.0145	0	52	0,0015	1	R.AECMLQQAER.L + Oxidation (M)
60	539.9177	1616.7313	1616.7641	-0.0329	1	30	0,17	1	K.TENLNDDEKLNNAK.Y

N° in Table S1	N° spot	protein identification
9	693	**lamim B1**<br>VEHULB Mass: 66653 Score: 480 Coverage: 27% number of peptides : 15<br>lamin B1 - human

1. Check to include this hit in error tolerant search or archive report

Query	Observed	Mr(expt)	Mr(calc)	Delta	Miss	Score	Expect	Rank	Peptide
4	415.7394	829.4642	829.4294	0.0349	0	49	0,0048	1	K.LSPSPSSR.V
8	437.7553	873.4960	873.4556	0.0405	0	42	0,021	1	R.LVEVDSGR.Q
11	485.2721	968.5296	968.4814	0.0482	0	64	7.9e-005	1	K.IGDTSVSYK.Y
14	487.7675	973.5204	973.4716	0.0489	0	40	0,03	1	K.LLEGEEER.L
15	512.7748	1023.5350	1023.4872	0.0478	0	31	0,16	1	R.EYEAALNSK.D
16	515.8153	1029.6160	1029.5706	0.0454	0	55	0,00087	1	K.QLADETLLK.V
17	537.7984	1073.5822	1073.5353	0.0470	0	53	0,0013	1	K.ESDLNGAQIK.L
19	559.2894	1116.5642	1116.5159	0.0483	0	38	0,04	1	K.NSQGEEVAQR.S
22	577.8395	1153.6644	1153.6091	0.0553	0	79	2.6e-006	1	R.AGGPTTPLSPTR.L
23	586.3619	1170.7092	1170.6496	0.0597	0	30	0,22	1	R.IQELEDLLAK.E
24	587.3559	1172.6972	1172.6401	0.0572	1	104	8.8e-009	1	K.DAALATALGDKK.S
25	626.3455	1250.6764	1250.6142	0.0622	0	56	0,00059	1	K.ALYETELADAR.R
26	667.8342	1333.6538	1333.5898	0.0640	0	49	0,0035	1	K.NQNSWGTGEDVK.V
27	708.8346	1415.6546	1415.5874	0.0672	0	55	0,00058	1	K.SMYEEEINETR.R + Oxidation (M)
28	756.8853	1511.7560	1511.6886	0.0675	0	130	2.1e-011	1	R.LSSEMNTSTVNSAR.E + Oxidation (M)

N° in Table S1	N° spot	protein identification
10	689	**lamim B1**

1. VEHULB Mass: 66653 Score: 490 Coverage: 25% number of peptides : 15
lamin B1 - human
Check to include this hit in error tolerant search or archive report

Query	Observed	Mr(expt)	Mr(calc)	Delta	Miss	Score	Expect	Rank	Peptide
1	400.7723	799.5300	799.4803	0.0497	0	28	0,46	1	K.LQIELGK.C
2	411.2684	820.5222	820.4694	0.0528	0	35	0,078	1	R.LAVYIDK.V
4	415.7461	829.4776	829.4294	0.0483	0	45	0,011	1	K.LSPSPSSR.V
8	437.7625	873.5104	873.4556	0.0549	0	42	0,022	1	R.LVEVDSGR.Q
12	485.2760	968.5374	968.4814	0.0560	0	64	7.7e-005	1	K.IGDTSVSYK.Y
16	487.7742	973.5338	973.4716	0.0623	0	46	0,007	1	K.LLEGEEER.L
18	512.7827	1023.5508	1023.4872	0.0636	0	46	0,0053	1	R.EYEAALNSK.D
19	515.8230	1029.6314	1029.5706	0.0608	0	62	0,0002	1	K.QLADETLLK.V
20	522.8178	1043.6210	1043.5611	0.0600	1	46	0,0059	1	R.LQEKEELR.E
22	549.3292	1096.6438	1096.5764	0.0674	1	45	0,0063	1	R.KIGDTSVSYK.Y
24	577.8475	1153.6804	1153.6091	0.0713	0	57	0,00049	1	R.AGGPTTPLSPTR.L
26	587.3626	1172.7106	1172.6401	0.0706	1	70	2.5e-005	1	K.DAALATALGDKK.S
27	667.8409	1333.6672	1333.5898	0.0774	0	51	0,0022	1	K.NQNSWGTGEDVK.V
28	708.8447	1415.6748	1415.5874	0.0874	0	55	0,00067	1	K.SMYEEEINETR.R + Oxidation (M)
30	756.8973	1511.7800	1511.6886	0.0915	0	130	2e-011	1	R.LSSEMNTSTVNSAR.E + Oxidation (M)

N° in Table S1	N° spot	protein identification
11	1019	**CapG**

2. A43358 Mass: 38779 Score: 767 Coverage: 20% number of peptides : 8
macrophage capping protein - human
Check to include this hit in error tolerant search or archive report

Query	Observed	Mr(expt)	Mr(calc)	Delta	Miss	Score	Expect	Rank	Peptide
7	499.8599	997.7052	997.6032	0.1020	1	42	0.013	1	K.ARDLALAIR.D
12	510.8261	1019.6376	1019.5399	0.0977	0	64	9.6e-005	1	K.ANAQAAALYK.V
16	522.8574	1043.7002	1043.5975	0.1028	1	55	0,00082	1	K.TSTGAPAAIKK.L
27	629.9046	1257.7946	1257.6677	0.1270	1	33	0,12	1	R.DLALAIRDSER.Q
30	640.8735	1279.7324	1279.6078	0.1247	0	97	4e-008	1	K.VSDATGQMNLTK.V + Oxidation (M)
36	659.3541	1316.6936	1316.5732	0.1205	0	82	1.2e-006	1	K.EGNPEEDLTADK.A
42	676.3806	1350.7466	1350.6204	0.1263	0	85	5.9e-007	1	K.YQEGGVESAFHK.T
57	550.6633	1648.9681	1648.8208	0.1472	1	57	0,00034	1	R.GLKYQEGGVESAFHK.T

N° in Table S1	N° spot	protein identification
12	783	**keratin 8 (K8)**

1. K2C8_HUMAN Mass: 53510 Score: 1182 Coverage: 45% number of peptides : 26
Keratin, type II cytoskeletal 8 (Cytokeratin 8) (K8) (CK 8).- Homo sapiens (Human).
Check to include this hit in error tolerant search or archive report

Query	Observed	Mr(expt)	Mr(calc)	Delta	Miss	Score	Expect	Rank	Peptide
2	414.2479	826.4812	826.4225	0.0588	0	38	0,038	1	K.FASFIDK.V
3	435.7549	869.4952	869.4243	0.0710	0	70	1.8e-005	1	R.ISSSSFSR.V
5	466.7748	931.5350	931.4610	0.0740	0	50	0,0026	1	K.LLEGEESR.L
6	500.8219	999.6292	999.5600	0.0692	0	53	0,0012	1	R.LQAEIEGLK.G

8	515.8284	1029.6422	1029.5607	0.0815	0	32	0,17	1	K.WSLLQQQK.T
13	523.3251	1044.6356	1044.5451	0.0905	0	55	0,00078	1	R.QLETLGQEK.L
14	530.8319	1059.6492	1059.5560	0.0933	1	63	0,00013	1	R.KLLEGEESR.L
15	533.8095	1065.6044	1065.5090	0.0954	1	47	0,005	1	K.YEDEINKR.T
16	540.3059	1078.5972	1078.5043	0.0929	0	49	0,0028	1	K.AQYEDIANR.S
17	540.3157	1078.6168	1078.5294	0.0874	0	36	0,057	1	R.QLYEEEIR.E
19	541.8482	1081.6818	1081.5920	0.0899	1	52	0,0013	1	K.FASFIDKVR.F
21	550.7804	1099.5462	1099.4492	0.0971	0	26	0.56	1	K.DVDEAYMNK.V + Oxidation (M)
24	565.3637	1128.7128	1128.6138	0.0990	1	83	1.1e-006	1	R.GELAIKDANAK.L
25	565.3646	1128.7146	1128.6138	0.1008	0	72	1.4e-005	1	K.LSELEAALQR.A
27	569.3269	1136.6392	1136.5713	0.0679	0	69	5.6e-005	1	K.YEELQSLAGK.H
29	577.3301	1152.6456	1152.5485	0.0972	0	46	0.0057	1	R.EYQELMNVK.L
33	587.3561	1172.6976	1172.6288	0.0688	0	68	4.2e-005	1	K.LVSESSDVLPK.-
36	593.3292	1184.6438	1184.5383	0.1055	0	62	0,00016	1	R.AEAESMYQIK.Y + Oxidation (M)
37	601.3842	1200.7538	1200.6462	0.1076	1	48	0,0037	1	R.RQLETLGQEK.L
40	612.8548	1223.6950	1223.5816	0.1135	1	49	0,00044	1	R.TKTEISEMNR.N + Oxidation (M)
45	668.8961	1335.7776	1335.6591	0.1185	0	87	4.1e-007	1	R.SLDMDSIIAEVK.A + Oxidation (M)
47	672.8967	1343.7788	1343.6681	0.1108	0	86	5.6e-007	1	R.ASLEAAIADAEQR.G
50	684.8989	1367.7832	1367.6642	0.1190	0	73	1.6e-005	1	R.TEMENEFVLIK.K + Oxidation (M)
52	714.9066	1427.7986	1427.6714	0.1272	1	64	6.8e-005	1	R.SRAEAESMYQIK.Y + Oxidation (M)
56	738.4141	1474.8136	1474.6908	0.1229	0	112	1.2e-009	1	R.LESGMQNMSIHTK.T
57	499.6308	1495.8706	1495.7592	0.1114	1	72	1.4e-005	1	h

N° in Table S1	N° spot	protein identification
**13 and 50**	**715**	**ER60, K8 and K6A**

50 — JC5704 Mass: 57160 Score:196 Coverage: 15% number of peptides : 7
protein disulfide-isomerase (EC 5.3.4.1) ER60 precursor - human
Check to include this hit in error tolerant search or archive report

Query	Observed	Mr(expt)	Mr(calc)	Delta	Miss	Score	Expect	Rank	Peptide
19	542.8403	1083.6660	1083.5600	0.1060	0	60	0,0002	1	K.YGVSGYPTLK.I
26	590.3557	1178.6968	1178.5792	0.1177	1	27	0,47	1	K.AASNLRDNYR.F
28	594.8289	1187.6432	1187.5281	0.1152	0	52	0,0015	1	K.FVMQEEFSR.D + Oxidation (M)
30	596.3607	1190.7068	1190.5931	0.1137	0	55	0,00076	1	R.LAPEYEAAATR.L
32	599.4230	1196.8314	1196.7128	0.1186	1	37	0,036	1	K.LSKDPNIVIAK.M
37	618.8224	1235.6302	1235.5054	0.1248	0	38	0,037	1	R.DGEEAGAYDGPR.T
51	790.5003	1578.9860	1578.8253	0.1607	0	42	0.0095	1	R.EATNPPVIQEEKPK.K

13 — K2C8_HUMAN Mass: 53510 Score: 286 Coverage: 11% number of peptides : 4
Keratin, type II cytoskeletal 8 (Cytokeratin 8) (K8) (CK 8).- Homo sapiens (Human).
Check to include this hit in error tolerant search or archive report

Query	Observed	Mr(expt)	Mr(calc)	Delta	Miss	Score	Expect	Rank	Peptide
24	565.3693	1128.7240	1128.6138	0.1102	0	49	0,0031	2	K.LSELEAALQR.A
39	668.9056	1335.7966	1335.6591	0.1375	0	61	0,00017	1	R.SLDMDSIIAEVK.A + Oxidation (M)
41	672.9132	1343.8118	1343.6681	0.1438	0	79	2.5e-006	1	R.ASLEAAIADAEQR.G
55	899.5122	1797.0098	1796.8250	0.1848	1	107	3.7e-009	1	K.DVDEAYMNKVELESR.L

KRHUEA Mass: 60293 Score: 97 Coverage: 9% number of peptides : 5
keratin 6a, type II - human
Check to include this hit in error tolerant search or archive report

13

Query	Observed	Mr(expt)	Mr(calc)	Delta	Miss	Score	Expect	Rank	Peptide
9	473.3047	944.5948	944.5039	0.0909	1	35	0,1	1	R.GRLDSELR.G
11	503.2877	1004.5608	1004.4597	0.1012	0	30	0,22	1	K.LLEGEECR.L
13	506.8061	1011.5976	1011.4985	0.0992	0	46	0,0053	1	R.SGFSSVSVSR.S
34	602.3854	1202.7562	1202.6295	0.1267	0	41	0,018	1	K.WTLLQEQGTK.T
42	675.9300	1349.8454	1349.7190	0.1264	1	43	0,014	1	R.TAAENEFVTLKK.D

N° in Table S1	N° spot	protein identification
**14**	**866**	**K17**

1. K1CQ_HUMAN Mass: 48230 Score: 2702 Coverage: 52% number of peptides : 28
Keratin, type I cytoskeletal 17 (Cytokeratin 17) (K17) (CK 17) (39.1).- Homo sapiens (Human).
Check to include this hit in error tolerant search or archive report

Query	Observed	Mr(expt)	Mr(calc)	Delta	Miss	Score	Expect	Rank	Peptide
1	404.1901	806.3656	806.3922	-0.0266	0	57	0,00056	1	R.LAADDFR.T
2	405.2026	808.3906	808.4330	-0.0424	0	35	0,069	1	R.LASYLDK.V
5	487.7318	973.4490	973.5080	-0.0589	0	36	0,064	1	R.TIEELQNK.I

Query	Observed	Mr(expt)	Mr(calc)	Delta	Miss	Score	Expect	Rank	Peptide
7	492.7357	983.4568	983.4924	-0.0355	0	56	0,0006	1	R.QFTSSSSIK.G
9	495.2571	988.4996	988.5301	-0.0305	1	36	0,069	1	K.SEISELRR.T
10	497.2414	992.4682	992.5542	-0.0860	1	30	0,29	1	K.EELAYLKK.N
11	497.6992	993.3838	993.4192	-0.0353	0	29	0,27	1	R.DYSQYYR.T
15	515.2935	1028.5724	1028.5866	-0.0141	0	53	0,0013	1	R.VLDELTLAR.A
24	517.7283	1033.4420	1033.4974	-0.0554	0	65	8e-005	1	R.LSGGLGAGSCR.L
27	518.7578	1035.5010	1035.5250	-0.0239	1	35	0,08	1	K.IRDWYQR.Q
29	539.7452	1077.4758	1077.4873	-0.0114	0	50	0,0023	1	K.ATMQNLNDR.L + Oxidation (M)
33	559.2590	1116.5034	1116.5662	-0.0628	0	70	2.2e-005	1	R.TIVEEVQDGK.V
39	561.7824	1121.5502	1121.5717	-0.0214	0	61	0,0002	1	R.LEQEIATYR.R
41	572.7481	1143.4816	1143.4873	-0.0056	0	46	0,0067	1	K.DAEDWFFSK.T
45	593.3437	1184.6728	1184.6877	-0.0148	1	41	0,018	1	R.RVLDELTLAR.A
46	593.8182	1185.6218	1185.6353	-0.0135	0	71	1.9e-005	1	R.LSVEADINGLR.R
48	611.7949	1221.5752	1221.6353	-0.0601	1	70	2e-005	1	R.TKFETEQALR.L
51	621.7675	1241.5204	1241.5458	-0.0254	0	65	7.7e-005	1	K.NHEEEMNALR.G
55	636.7943	1271.5740	1271.5822	-0.0082	1	80	5.1e-006	1	R.KDAEDWFFSK.T
61	660.3223	1318.6300	1318.6438	-0.0138	0	86	4,90E-07	1	R.ADLEMQIENLK.E + Oxidation (M)
66	671.8676	1341.7206	1341.7364	-0.0158	1	66	5.2e-005	1	R.LSVEADINGLRR.V
71	673.3010	1344.5874	1344.6772	-0.0898	0	115	7.7e-010	1	R.ALEEANTELEVK.I
77	681.3477	1360.6808	1360.6834	-0.0025	0	99	3.2e-008	1	R.EVATNSELVQSGK.S
80	690.3457	1378.6768	1378.7204	-0.0436	1	91	1.7e-007	1	K.TRLEQEIATYR.R
87	702.2980	1402.5814	1402.6688	-0.0873	0	113	1.1e-009	1	K.ASLEGNLAETENR.Y
95	751.7925	1501.5704	1501.5813	-0.0109	0	86	5.2e-007	1	R.CEMEQQNQEYK.I + Oxidation (M)
100	758.8665	1515.7184	1515.7569	-0.0384	0	118	3e-010	1	R.LLEGEDAHLTQYK.K
106	558.2799	1671.8179	1671.8580	-0.0401	1	91	1.6e-007	1	R.RLLEGEDAHLTQYK.K

N° in Table S1	N° spot	protein identification
15	1063	K17 and K14

1. K1CQ_HUMAN Mass: 48230 Score: 1278 Coverage: 45% — number of peptides : 23

Keratin, type I cytoskeletal 17 (Cytokeratin 17) (K17) (CK 17) (39.1).- Homo sapiens (Human).

Check to include this hit in error tolerant search or archive report

Query	Observed	Mr(expt)	Mr(calc)	Delta	Miss	Score	Expect	Rank	Peptide
1	404.2172	806.4198	806.3922	0.0276	0	43	0,014	1	R.LAADDFR.T
2	405.2363	808.4580	808.4330	0.0250	0	33	0,12	1	R.LASYLDK.V
8	487.7757	973.5368	973.5080	0.0289	0	37	0,055	1	R.TIEELQNK.I
9	492.7670	983.5194	983.4924	0.0271	0	58	0,00035	1	R.QFTSSSSIK.G
11	495.2862	988.5578	988.5301	0.0277	1	33	0,13	1	K.SEISELRR.T
12	497.2723	992.5300	992.4927	0.0374	0	52	0,0018	1	K.FETEQALR.L
14	497.7352	993.4558	993.4192	0.0367	0	26	0,6	1	R.DYSQYYR.T
15	515.3189	1028.6232	1028.5866	0.0367	0	43	0,014	1	R.VLDELTLAR.A
16	517.7715	1033.5284	1033.4974	0.0310	0	68	3.7e-005	1	R.LSGGLGAGSCR.L
17	518.7886	1035.5626	1035.5250	0.0377	1	27	0,5	1	K.IRDWYQR.Q
23	539.7677	1077.5208	1077.4873	0.0336	0	67	5.2e-005	1	K.ATMQNLNDR.L + Oxidation (M)
26	559.3101	1116.6056	1116.5662	0.0394	0	66	6.2e-005	1	R.TIVEEVQDGK.V
28	561.8143	1121.6140	1121.5717	0.0424	0	52	0,0016	1	R.LEQEIATYR.R
29	572.7736	1143.5326	1143.4873	0.0454	0	36	0,06	1	K.DAEDWFFSK.T
36	611.8444	1221.6742	1221.6353	0.0389	1	72	1.2e-005	1	R.TKFETEQALR.L
42	629.7987	1257.5828	1257.5408	0.0421	0	43	0,012	1	K.NHEEEMNALR.G + Oxidation (M)
46	673.3731	1344.7316	1344.6772	0.0544	0	99	3e-008	1	R.ALEEANTELEVK.I
48	681.3714	1360.7282	1360.6834	0.0449	0	95	7.7e-007	1	R.EVATNSELVQSGK.S
51	690.3946	1378.7746	1378.7204	0.0542	1	64	9.3e-005	1	K.TRLEQEIATYR.R
53	462.8973	1385.6701	1385.6357	0.0344	1	38	0,03	1	K.KNHEEEMNALR.G + Oxidation (M)
54	702.3699	1402.7252	1402.6688	0.0565	0	82	1.2e-006	1	K.ASLEGNLAETENR.Y
59	751.8244	1501.6342	1501.5813	0.0529	0	89	2.7e-007	1	R.CEMEQQNQEYK.I + Oxidation (M)
61	548.9725	1643.8957	1643.8518	0.0438	1	59	0,0002	1	R.LLEGEDAHLTQYKK.E

KRHUE Mass: 51916 Score: 633 Coverage: 28%

15 keratin 14, type I, cytoskeletal - human — number of peptides : 15 with 4 specific for K14 in comparison with K17

2. Check to include this hit in error tolerant search or archive report

Query	Observed	Mr(expt)	Mr(calc)	Delta	Miss	Score	Expect	Rank	Peptide
1	404.2172	806.4198	806.3922	0.0276	0	43	0,014	1	R.LAADDFR.T
2	405.2363	808.4580	808.4330	0.0250	0	33	0,12	1	R.LASYLDK.V
11	495.2862	988.5578	988.5301	0.0277	1	33	0,13	1	K.SEISELRR.T
15	515.3189	1028.6232	1028.5866	0.0367	0	43	0,014	1	R.VLDELTLAR.A
17	518.7886	1035.5626	1035.5250	0.0377	1	27	0,5	1	K.IRDWYQR.Q
18	519.2889	1036.5632	1036.5189	0.0444	0	30	0,24	1	K.YETELNLR.M

24	553.7845	1105.5544	1105.5186	0.0359	0	66	6.9e-005	1	K.VTMQNLNDR.L + Oxidation (M)
25	553.8028	1105.5910	1105.5549	0.0361	0	54	0,0009	1	R.ISSVLAGGSCR.A
28	561.8143	1121.6140	1121.5717	0.0424	0	52	0,0016	1	R.LEQEIATYR.R
32	610.8260	1219.6374	1219.5932	0.0443	0	74	9e-006	1	K.ASLENSLEETK.G
42	629.7987	1257.5828	1257.5408	0.0421	0	43	0,012	1	K.NHEEEMNALR.G + Oxidation (M)
48	681.3714	1360.7282	1360.6834	0.0449	0	95	7.7e-008	1	R.EVATNSELVQSGK.S
51	690.3946	1378.7746	1378.7204	0.0542	1	64	9.3e-005	1	K.TRLEQEIATYR.R
53	462.8973	1385.6701	1385.6357	0.0344	1	38	0,03	1	K.KNHEEEMNALR.G + Oxidation (M)
59	751.8244	1501.6342	1501.5813	0.0529	0	89	2.7e-007	1	R.CEMEQQNQEYK.I + Oxidation (M)

N° in Table S1	N° spot	protein identification
16	854	K17 and K14

1. K1CQ_HUMAN Mass: 48230 Score: 1487 Coverage: 39% number of peptides : 21
Keratin, type I cytoskeletal 17 (Cytokeratin 17) (K17) (CK 17) (39.1).- Homo sapiens (Human).
Check to include this hit in error tolerant search or archive report

Query	Observed	Mr(expt)	Mr(calc)	Delta	Miss	Score	Expect	Rank	Peptide
1	404.2360	806.4574	806.3923	0.0652	0	43	0.014	1	R.LAADDFR.T
5	417.2530	832.4914	832.4290	0.0624	0	35	0.081	1	K.SEISELR.R
7	487.8008	973.5870	973.5080	0.0791	0	37	0.058	1	R.TIEELQNK.I
8	492.7920	983.5694	983.4924	0.0771	0	49	0.0029	1	R.QFTSSSSIK.G
9	497.3239	992.6332	992.4927	0.1406	0	33	0.13	1	K.FETEQALR.L
13	515.3411	1028.6676	1028.5866	0.0811	0	50	0.0028	1	R.VLDELTLAR.A
16	517.7889	1033.5632	1033.4975	0.0658	0	68	3.7e-005	1	R.LSGGLGAGSCR.L
21	539.7940	1077.5734	1077.4873	0.0862	0	51	0.0021	1	K.ATMQNLNDR.L + Oxidation (M)
25	559.3331	1116.6516	1116.5663	0.0854	0	66	6.6e-005	1	R.TIVEEVQDGK.V
29	561.8388	1121.6630	1121.5717	0.0914	0	54	0.00094	1	R.LEQEIATYR.R
32	593.3966	1184.7786	1184.6877	0.0910	1	52	0.0013	1	R.RVLDELTLAR.A
33	593.8734	1185.7322	1185.6353	0.0969	0	47	0.0054	1	R.LSVEADINGLR.R
38	611.8728	1221.7310	1221.6353	0.0957	1	71	1.8e-005	1	R.TKFETEQALR.L
44	629.8298	1257.6450	1257.5408	0.1043	0	48	0.0036	1	K.NHEEEMNALR.G + Oxidation (M)
48	448.2847	1341.8323	1341.7364	0.0958	1	37	0.037	1	R.LSVEADINGLRR.V
51	673.4006	1344.7866	1344.6772	0.1094	0	100	2.2e-008	1	R.ALEEANTELEVK.I
58	681.3838	1360.7530	1360.6834	0.0696	0	109	2.8e-009	1	R.EVATNSELVQSGK.S
63	690.4233	1378.8320	1378.7204	0.1116	1	75	6e-006	1	K.TRLEQEIATYR.R
64	462.9176	1385.7310	1385.6357	0.0953	1	68	3.4e-005	1	K.KNHEEEMNALR.G + Oxidation (M)
66	702.3848	1402.7550	1402.6688	0.0863	0	106	4.6e-009	1	K.ASLEGNLAETENR.Y
70	751.8578	1501.7010	1501.5813	0.1197	0	89	2.7e-007	1	R.CEMEQQNQEYK.I + Oxidation (M)

KRHUE Mass: 51916 Score: 792 Coverage: 20% number of peptides : 12 with 2 specific for K14 in comparison with K17
keratin 14, type I, cytoskeletal - human
Check to include this hit in error tolerant search or archive report

16

Query	Observed	Mr(expt)	Mr(calc)	Delta	Miss	Score	Expect	Rank	Peptide
1	404.2360	806.4574	806.3923	0.0652	0	43	0.014	1	R.LAADDFR.T
5	417.2530	832.4914	832.4290	0.0624	0	35	0.081	1	K.SEISELR.R
13	515.3411	1028.6676	1028.5866	0.0811	0	50	0.0028	1	R.VLDELTLAR.A
22	553.8110	1105.6074	1105.5186	0.0888	0	86	7e-007	1	K.VTMQNLNDR.L + Oxidation (M)
29	561.8388	1121.6630	1121.5717	0.0914	0	54	0.00094	1	R.LEQEIATYR.R
32	593.3966	1184.7786	1184.6877	0.0910	1	52	0.0013	1	R.RVLDELTLAR.A
35	610.8543	1219.6940	1219.5932	0.1009	0	75	6.8e-006	1	K.ASLENSLEETK.G
44	629.8298	1257.6450	1257.5408	0.1043	0	48	0.0036	1	K.NHEEEMNALR.G + Oxidation (M)
58	681.3838	1360.7530	1360.6834	0.0696	0	109	2.8e-009	1	R.EVATNSELVQSGK.S
63	690.4233	1378.8320	1378.7204	0.1116	1	75	6e-006	1	K.TRLEQEIATYR.R
64	462.9176	1385.7310	1385.6357	0.0953	1	68	3.4e-005	1	K.KNHEEEMNALR.G + Oxidation (M)
70	751.8578	1501.7010	1501.5813	0.1197	0	89	2.7e-007	1	R.CEMEQQNQEYK.I + Oxidation (M)

N° in Table S1	N° spot	protein identification
17	1054	K17 and K14

1. K1CQ_HUMAN Mass: 48230 Score: 1195 Coverage: 43% number of peptides : 23
Keratin, type I cytoskeletal 17 (Cytokeratin 17) (K17) (CK 17) (39.1).- Homo sapiens (Human).
Check to include this hit in error tolerant search or archive report

Query	Observed	Mr(expt)	Mr(calc)	Delta	Miss	Score	Expect	Rank	Peptide
1	404.1989	806.3832	806.3923	-0.0090	0	43	0.015	1	R.LAADDFR.T
2	405.2188	808.4230	808.4330	-0.0100	0	24	0.98	1	R.LASYLDK.V
7	487.7562	973.4978	973.5080	-0.0101	0	31	0.24	1	R.TIEELQNK.I
9	492.7485	983.4824	983.4924	-0.0099	0	52	0.0016	1	R.QFTSSSSIK.G

12	495.2681	988.5216	988.5301	-0.0085	1	36	0.069	1	K.SEISELRR.T
13	497.2530	992.4914	992.4927	-0.0012	0	35	0.078	1	K.FETEQALR.L
14	497.7109	993.4072	993.4192	-0.0119	0	30	0.23	1	R.DYSQYYR.T
16	515.2944	1028.5742	1028.5866	-0.0123	0	44	0.01	1	R.VLDELTLAR.A
17	517.7523	1033.4900	1033.4975	-0.0074	0	63	0.00012	1	R.LSGGLGAGSCR.L
18	518.7648	1035.5150	1035.5250	-0.0099	1	33	0.12	1	K.IRDWYQR.Q
22	531.7476	1061.4806	1061.4924	-0.0117	0	63	0.00013	1	K.ATMQNLNDR.L
28	559.2860	1116.5574	1116.5663	-0.0088	0	62	0.00017	1	R.TIVEEVQDGK.V
29	561.7889	1121.5632	1121.5717	-0.0084	0	61	0.00018	1	R.LEQEIATYR.R
30	593.3457	1184.6768	1184.6877	-0.0109	1	75	7.9e-006	1	R.RVLDELTLAR.A
34	611.8211	1221.6276	1221.6353	-0.0077	1	66	4.9e-005	1	R.TKFETEQALR.L
38	629.7746	1257.5346	1257.5408	-0.0061	0	36	0.055	1	K.NHEEEMNALR.G + Oxidation (M)
42	673.3407	1344.6668	1344.6772	-0.0104	0	100	2.2e-008	1	R.ALEEANTELEVK.I
45	681.3314	1360.6482	1360.6834	-0.0352	0	89	2.9e-007	1	R.EVATNSELVQSGK.S
49	690.3664	1378.7182	1378.7204	-0.0022	1	75	6.1e-006	1	K.TRLEQEIATYR.R
50	462.8745	1385.6017	1385.6357	-0.0340	1	44	0.0073	1	K.KNHEEEMNALR.G + Oxidation (M)
51	702.3365	1402.6584	1402.6688	-0.0103	0	129	2.6e-011	1	K.ASLEGNLAETENR.Y
55	751.7926	1501.5706	1501.5813	-0.0107	0	89	2.5e-007	1	R.CEMEQQNQEYK.I + Oxidation (M)
59	548.9495	1643.8267	1643.8519	-0.0252	1	61	0.00013	1	R.LLEGEDAHLTQYKK.E

KRHUE Mass: 51916 Score: 728 Coverage: 22% number of peptides : 14 with 2 specific for K14 in comparison with K17

17 keratin 14, type I, cytoskeletal - human

Check to include this hit in error tolerant search or archive report

Query	Observed	Mr(expt)	Mr(calc)	Delta	Miss	Score	Expect	Rank	Peptide
1	404.1989	806.3832	806.3923	-0.0090	0	43	0.015	1	R.LAADDFR.T
2	405.2188	808.4230	808.4330	-0.0100	0	24	0.98	1	R.LASYLDK.V
12	495.2681	988.5216	988.5301	-0.0085	1	36	0.069	1	K.SEISELRR.T
16	515.2944	1028.5742	1028.5866	-0.0123	0	44	0.01	1	R.VLDELTLAR.A
18	518.7648	1035.5150	1035.5250	-0.0099	1	33	0.12	1	K.IRDWYQR.Q
26	553.7802	1105.5458	1105.5550	-0.0091	0	47	0.0055	1	R.ISSVLAGGSCR.A
29	561.7889	1121.5632	1121.5717	-0.0084	0	61	0.00018	1	R.LEQEIATYR.R
30	593.3457	1184.6768	1184.6877	-0.0109	1	75	7.9e-006	1	R.RVLDELTLAR.A
31	610.8000	1219.5854	1219.5932	-0.0077	0	94	9.2e-008	1	K.ASLENSLEETK.G
38	629.7746	1257.5346	1257.5408	-0.0061	0	36	0.055	1	K.NHEEEMNALR.G + Oxidation (M)
45	681.3314	1360.6482	1360.6834	-0.0352	0	89	2.9e-007	1	R.EVATNSELVQSGK.S
49	690.3664	1378.7182	1378.7204	-0.0022	1	75	6.1e-006	1	K.TRLEQEIATYR.R
50	462.8745	1385.6017	1385.6357	-0.0340	1	44	0.0073	1	K.KNHEEEMNALR.G + Oxidation (M)
55	751.7926	1501.5706	1501.5813	-0.0107	0	89	2.5e-007	1	R.CEMEQQNQEYK.I + Oxidation (M)

N° in Table S1	N° spot	protein identification
18	986	**K17 and K14 and K16**

1. K1CQ_HUMAN Mass: 48230 Score: 751 Coverage: 40% number of peptides : 19

Keratin, type I cytoskeletal 17 (Cytokeratin 17) (K17) (CK 17) (39.1).- Homo sapiens (Human).

Check to include this hit in error tolerant search or archive report

Query	Observed	Mr(expt)	Mr(calc)	Delta	Miss	Score	Expect	Rank	Peptide
1	404.2005	806.3864	806.3923	-0.0058	0	40	0.029	1	R.LAADDFR.T
2	405.2203	808.4260	808.4330	-0.0070	0	44	0.01	1	R.LASYLDK.V
9	487.7583	973.5020	973.5080	-0.0059	0	32	0.19	1	R.TIEELQNK.I
11	492.7505	983.4864	983.4924	-0.0059	0	49	0.0028	1	R.QFTSSSSIK.G
12	495.2688	988.5230	988.5301	-0.0071	1	38	0.043	1	K.SEISELRR.T
14	515.2975	1028.5804	1028.5866	-0.0061	0	46	0.0067	1	R.VLDELTLAR.A
15	517.7520	1033.4894	1033.4975	-0.0080	0	56	0.00065	1	R.LSGGLGAGSCR.L
16	518.7676	1035.5206	1035.5250	-0.0043	1	32	0.14	1	K.IRDWYQR.Q
22	532.8054	1063.5962	1063.6026	-0.0063	1	28	0.37	1	R.LASYLDKVR.A
24	539.7459	1077.4772	1077.4873	-0.0100	0	67	5.3e-005	1	K.ATMQNLNDR.L + Oxidation (M)
28	559.2867	1116.5588	1116.5663	-0.0074	0	66	6.2e-005	1	R.TIVEEVQDGK.V
30	561.7925	1121.5704	1121.5717	-0.0012	0	61	0.00022	1	R.LEQEIATYR.R
34	611.8231	1221.6316	1221.6353	-0.0037	1	52	0.0015	1	R.TKFETEQALR.L
38	629.7725	1257.5304	1257.5408	-0.0103	0	48	0.0037	1	K.NHEEEMNALR.G + Oxidation (M)
44	673.3444	1344.6742	1344.6772	-0.0030	0	89	2.8e-007	1	R.ALEEANTELEVK.I
46	681.3449	1360.6752	1360.6834	-0.0082	0	92	1.5e-007	1	R.EVATNSELVQSGK.S
51	702.3395	1402.6644	1402.6688	-0.0043	0	78	3.1e-006	1	K.ASLEGNLAETENR.Y
52	485.5635	1453.6687	1453.6871	-0.0184	1	47	0.0035	1	R.ILNEMRDQYEK.M + Oxidation (M)
56	751.7924	1501.5702	1501.5813	-0.0111	0	89	2.6e-007	1	R.CEMEQQNQEYK.I + Oxidation (M)

KRHUE Mass: 51916 Score: 523 Coverage: 26% number of peptides : 14 with 3 specific for K14 in comparison with K17

18 keratin 14, type I, cytoskeletal - human

2. Check to include this hit in error tolerant search or archive report

Query	Observed	Mr(expt)	Mr(calc)	Delta	Miss	Score	Expect	Rank	Peptide
1	404.2005	806.3864	806.3923	-0.0058	0	40	0.029	1	R.LAADDFR.T
2	405.2203	808.4260	808.4330	-0.0070	0	44	0.01	1	R.LASYLDK.V
12	495.2688	988.5230	988.5301	-0.0071	1	38	0.043	1	K.SEISELRR.T
14	515.2975	1028.5804	1028.5866	-0.0061	0	46	0.0067	1	R.VLDELTLAR.A
16	518.7676	1035.5206	1035.5250	-0.0043	1	32	0.14	1	K.IRDWYQR.Q
22	532.8054	1063.5962	1063.6026	-0.0063	1	28	0.37	1	R.LASYLDKVR.A
**25**	**553.7610**	**1105.5074**	**1105.5186**	**-0.0112**	**0**	**86**	**6.5e-007**	**1**	**K.VTMQNLNDR.L + Oxidation (M)**
**26**	**553.7827**	**1105.5508**	**1105.5550**	**-0.0041**	**0**	**45**	**0.008**	**1**	**R.ISSVLAGGSCR.A**
30	561.7925	1121.5704	1121.5717	-0.0012	0	61	0.00022	1	R.LEQEIATYR.R
**33**	**610.8013**	**1219.5880**	**1219.5932**	**-0.0051**	**0**	**69**	**3.2e-005**	**1**	**K.ASLENSLEETK.G**
38	629.7725	1257.5304	1257.5408	-0.0103	0	48	0.0037	1	K.NHEEEMNALR.G + Oxidation (M)
46	681.3449	1360.6752	1360.6834	-0.0082	0	92	1.5e-007	1	R.EVATNSELVQSGK.S
52	485.5635	1453.6687	1453.6871	-0.0184	1	47	0.0035	1	R.ILNEMRDQYEK.M + Oxidation (M)
56	751.7924	1501.5702	1501.5813	-0.0111	0	89	2.6e-007	1	R.CEMEQQNQEYK.I + Oxidation (M)

K1CP_HUMAN Mass: 51447 Score: 223 Coverage: 17% number of peptides : 10 with 1 specific for K16 in comparison with K14
Keratin, type I cytoskeletal 16 (Cytokeratin 16) (K16) (CK 16).- Homo sapiens (Human).

3. Check to include this hit in error tolerant search or archive report

18

Query	Observed	Mr(expt)	Mr(calc)	Delta	Miss	Score	Expect	Rank	Peptide
1	404.2005	806.3864	806.3923	-0.0058	0	40	0.029	1	R.LAADDFR.T
2	405.2203	808.4260	808.4330	-0.0070	0	44	0.01	1	R.LASYLDK.V
14	515.2975	1028.5804	1028.5866	-0.0061	0	46	0.0067	1	R.VLDELTLAR.T
16	518.7676	1035.5206	1035.5250	-0.0043	1	32	0.14	1	K.IRDWYQR.Q
22	532.8054	1063.5962	1063.6026	-0.0063	1	28	0.37	1	R.LASYLDKVR.A
25	553.7610	1105.5074	1105.5186	-0.0112	0	86	6.5e-007	1	K.VTMQNLNDR.L + Oxidation (M)
26	553.7827	1105.5508	1105.5550	-0.0041	0	45	0.008	1	R.ISSVLAGGSCR.A
30	561.7925	1121.5704	1121.5717	-0.0012	0	61	0.00022	1	R.LEQEIATYR.R
33	610.8013	1219.5880	1219.5932	-0.0051	0	69	3.2e-005	1	K.ASLENSLEETK.G
**39**	**630.7877**	**1259.5608**	**1259.5630**	**-0.0021**	**0**	**46**	**0.0057**	**1**	**R.EVFTSSSSSSR.Q**

N° in Table S1	N° spot	protein identification
**19**	**1046**	**K17 and 14**

1. K1CQ_HUMAN Mass: 48230 Score: 932 Coverage: 47% number of peptides : 24
Keratin, type I cytoskeletal 17 (Cytokeratin 17) (K17) (CK 17) (39.1).- Homo sapiens (Human).
Check to include this hit in error tolerant search or archive report

Query	Observed	Mr(expt)	Mr(calc)	Delta	Miss	Score	Expect	Rank	Peptide
1	**404.1957**	**806.3768**	**806.3923**	**-0.0154**	**0**	**31**	**0.22**	**1**	**R.LAADDFR.T**
2	**405.2173**	**808.4200**	**808.4330**	**-0.0130**	**0**	**41**	**0.019**	**1**	**R.LASYLDK.V**
5	**417.2150**	**832.4154**	**832.4290**	**-0.0136**	**0**	**26**	**0.7**	**1**	**K.SEISELR.R**
9	**487.7504**	**973.4862**	**973.5080**	**-0.0217**	**0**	**37**	**0.058**	**1**	**R.TIEELQNK.I**
11	**492.7466**	**983.4786**	**983.4924**	**-0.0137**	**0**	**49**	**0.0031**	**1**	**R.QFTSSSSIK.G**
12	**495.2643**	**988.5140**	**988.5301**	**-0.0161**	**1**	**36**	**0.068**	**1**	**K.SEISELRR.T**
13	**497.2630**	**992.5114**	**992.4927**	**0.0188**	**0**	**46**	**0.0063**	**1**	**K.FETEQALR.L**
15	**515.2913**	**1028.5680**	**1028.5866**	**-0.0185**	**0**	**46**	**0.006**	**1**	**R.VLDELTLAR.A**
17	**517.7479**	**1033.4812**	**1033.4975**	**-0.0162**	**0**	**66**	**6.4e-005**	**1**	**R.LSGGLGAGSCR.L**
22	**531.7451**	**1061.4756**	**1061.4924**	**-0.0167**	**0**	**63**	**0.00014**	**1**	**K.ATMQNLNDR.L**
26	**559.2808**	**1116.5470**	**1116.5663**	**-0.0192**	**0**	**61**	**0.00017**	**1**	**R.TIVEEVQDGK.V**
29	**561.7832**	**1121.5518**	**1121.5717**	**-0.0198**	**0**	**36**	**0.061**	**1**	**R.LEQEIATYR.R**
32	**611.8185**	**1221.6224**	**1221.6353**	**-0.0129**	**1**	**74**	**8e-006**	**1**	**R.TKFETEQALR.L**
33	**629.7666**	**1257.5186**	**1257.5408**	**-0.0221**	**0**	**48**	**0.0035**	**1**	**K.NHEEEMNALR.G + Oxidation (M)**
34	**660.3214**	**1318.6282**	**1318.6438**	**-0.0156**	**0**	**45**	**0.0065**	**1**	**R.ADLEMQIENLK.E + Oxidation (M)**
35	**671.8688**	**1341.7230**	**1341.7364**	**-0.0134**	**1**	**47**	**0.0042**	**1**	**R.LSVEADINGLRR.V**
36	**673.3373**	**1344.6600**	**1344.6772**	**-0.0172**	**0**	**100**	**2.3e-008**	**1**	**R.ALEEANTELEVK.I**
37	**681.3411**	**1360.6676**	**1360.6834**	**-0.0158**	**0**	**89**	**2.9e-007**	**1**	**R.EVATNSELVQSGK.S**
40	**690.3581**	**1378.7016**	**1378.7204**	**-0.0188**	**1**	**61**	**0.00017**	**1**	**K.TRLEQEIATYR.R**
42	**702.3337**	**1402.6528**	**1402.6688**	**-0.0159**	**0**	**97**	**3.7e-008**	**1**	**K.ASLEGNLAETENR.Y**
44	**727.8416**	**1453.6686**	**1453.6871**	**-0.0184**	**1**	**52**	**0.0013**	**1**	**R.ILNEMRDQYEK.M + Oxidation (M)**
45	**743.7952**	**1485.5758**	**1485.5864**	**-0.0105**	**0**	**90**	**2.1e-007**	**1**	**R.CEMEQQNQEYK.I**
48	**758.8800**	**1515.7454**	**1515.7569**	**-0.0114**	**0**	**100**	**2e-008**	**1**	**R.LLEGEDAHLTQYK.K**
49	**548.9440**	**1643.8102**	**1643.8519**	**-0.0417**	**1**	**69**	**2.5e-005**	**1**	**R.LLEGEDAHLTQYKK.E**

KRHUE Mass: 51916 Score:400 Coverage: 22% number of peptides : 13 with 2 specific for K14 in comparison with K17

19 keratin 14, type I, cytoskeletal - human

Check to include this hit in error tolerant search or archive report

Query	Observed	Mr(expt)	Mr(calc)	Delta	Miss	Score	Expect	Rank	Peptide
1	404.1957	806.3768	806.3923	-0.0154	0	31	0.22	1	R.LAADDFR.T
2	405.2173	808.4200	808.4330	-0.0130	0	41	0.019	1	R.LASYLDK.V
5	417.2150	832.4154	832.4290	-0.0136	0	26	0.7	1	K.SEISELR.R
12	495.2643	988.5140	988.5301	-0.0161	1	36	0.068	1	K.SEISELRR.T
15	515.2913	1028.5680	1028.5866	-0.0185	0	46	0.006	1	R.VLDELTLAR.A
**23**	**553.7594**	**1105.5042**	**1105.5186**	**-0.0144**	**0**	**43**	**0.013**	**1**	**K.VTMQNLNDR.L + Oxidation (M)**
**24**	**553.7761**	**1105.5376**	**1105.5550**	**-0.0173**	**0**	**49**	**0.003**	**1**	**R.ISSVLAGGSCR.A**
29	561.7832	1121.5518	1121.5717	-0.0198	0	36	0.061	1	R.LEQEIATYR.R
33	629.7666	1257.5186	1257.5408	-0.0221	0	48	0.0035	1	K.NHEEEMNALR.G + Oxidation (M)
37	681.3411	1360.6676	1360.6834	-0.0158	0	89	2.9e-007	1	R.EVATNSELVQSGK.S
40	690.3581	1378.7016	1378.7204	-0.0188	1	61	0.00017	1	K.TRLEQEIATYR.R
44	727.8416	1453.6686	1453.6871	-0.0184	1	52	0.0013	1	R.ILNEMRDQYEK.M + Oxidation (M)
45	743.7952	1485.5758	1485.5864	-0.0105	0	90	2.1e-007	1	R.CEMEQQNQEYK.I

N° in Table S1	N° spot	protein identification
**20**	**821**	**K14 and K17**

K1CN_HUMAN Mass: 51744 Score: 565 Coverage: 28% — number of peptides : 14

Keratin, type I cytoskeletal 14 (Cytokeratin 14) (K14) (CK 14).- Homo sapiens (Human).

Check to include this hit in error tolerant search or archive report

20

Query	Observed	Mr(expt)	Mr(calc)	Delta	Miss	Score	Expect	Rank	Peptide
2	404.1964	806.3782	806.3923	-0.0140	0	34	0.11	1	R.LAADDFR.T
3	405.2180	808.4214	808.4330	-0.0116	0	24	0.9	1	R.LASYLDK.V
15	515.2958	1028.5770	1028.5866	-0.0095	0	42	0.016	1	R.VLDELTLAR.A
18	532.8032	1063.5918	1063.6026	-0.0107	1	49	0.0028	1	R.LASYLDKVR.A
**19**	**545.7590**	**1089.5034**	**1089.5237**	**-0.0202**	**0**	**62**	**0.00015**	**1**	**K.VTMQNLNDR.L**
**25**	**553.7770**	**1105.5394**	**1105.5550**	**-0.0155**	**0**	**42**	**0.016**	**1**	**R.ISSVLAGGSCR.A**
**38**	**610.7980**	**1219.5814**	**1219.5932**	**-0.0117**	**0**	**39**	**0.03**	**1**	**K.ASLENSLEETK.G**
**44**	**633.8361**	**1265.6576**	**1265.6615**	**-0.0039**	**1**	**45**	**0.0063**	**1**	**R.TKYETELNLR.M**
**45**	**639.7897**	**1277.5648**	**1277.5783**	**-0.0134**	**0**	**41**	**0.017**	**1**	**K.GSCGIGGGIGGGSSR.I**
47	651.3268	1300.6390	1300.6510	-0.0120	0	118	3.5e-010	1	R.ALEEANADLEVK.I
**48**	**681.3401**	**1360.6656**	**1360.6834**	**-0.0178**	**0**	**89**	**3.2e-007**	**1**	**R.EVATNSELVQSGK.S**
50	690.3669	1378.7192	1378.7204	-0.0012	1	39	0.029	1	K.TRLEQEIATYR.R
**54**	**713.3475**	**1424.6804**	**1424.6896**	**-0.0091**	**0**	**91**	**1.7e-007**	**1**	**R.APSTYGGGLSVSSSR.F**
**56**	**478.5715**	**1432.6927**	**1432.7157**	**-0.0231**	**1**	**96**	**4.7e-008**	**1**	**K.ASLENSLEETKGR.Y**

K1CQ_HUMAN Mass: 48230 Score: 147 Coverage: 17% — number of peptides : 8 with 2 specific for K17 in comparison with K14

Keratin, type I cytoskeletal 17 (Cytokeratin 17) (K17) (CK 17) (39.1).- Homo sapiens (Human).

20 Check to include this hit in error tolerant search or archive report

Query	Observed	Mr(expt)	Mr(calc)	Delta	Miss	Score	Expect	Rank	Peptide
2	404.1964	806.3782	806.3923	-0.0140	0	34	0.11	1	R.LAADDFR.T
3	405.2180	808.4214	808.4330	-0.0116	0	24	0.9	1	R.LASYLDK.V
15	515.2958	1028.5770	1028.5866	-0.0095	0	42	0.016	1	R.VLDELTLAR.A
18	532.8032	1063.5918	1063.6026	-0.0107	1	49	0.0028	1	R.LASYLDKVR.A
**32**	**572.7475**	**1143.4804**	**1143.4873**	**-0.0068**	**0**	**33**	**0.13**	**1**	**K.DAEDWFFSK.T**
48	681.3401	1360.6656	1360.6834	-0.0178	0	89	3.2e-007	1	R.EVATNSELVQSGK.S
50	690.3669	1378.7192	1378.7204	-0.0012	1	39	0.029	1	K.TRLEQEIATYR.R
**69**	**941.4813**	**1880.9480**	**1880.9223**	**0.0257**	**0**	**39**	**0.02**	**1**	**R.TMQALEIELQSQLSMK.A + 2 Oxidation (M)**

N° in Table S1	N° spot	protein identification
**21**	**1023**	**K14**

K1CN_HUMAN Mass: 51744 Score: 2093 Coverage: 46% — number of peptides : 26

Keratin, type I cytoskeletal 14 (Cytokeratin 14) (K14) (CK 14).- Homo sapiens (Human).

Check to include this hit in error tolerant search or archive report

Query	Observed	Mr(expt)	Mr(calc)	Delta	Miss	Score	Expect	Rank	Peptide
**1**	**405.2167**	**808.4188**	**808.4330**	**-0.0142**	**0**	**41**	**0.019**	**1**	**R.LASYLDK.V**
**2**	**417.2251**	**832.4356**	**832.4290**	**0.0066**	**0**	**31**	**0.22**	**1**	**K.SEISELR.R**
**3**	**421.2540**	**840.4934**	**840.4817**	**0.0117**	**0**	**35**	**0.062**	**1**	**R.QRPAEIK.D**
**7**	**501.7215**	**1001.4284**	**1001.4488**	**-0.0203**	**0**	**66**	**6.7e-005**	**1**	**R.QFTSSSSMK.G**
**13**	**515.3062**	**1028.5978**	**1028.5866**	**0.0113**	**0**	**46**	**0.0067**	**1**	**R.VLDELTLAR.A**
**21**	**518.7637**	**1035.5128**	**1035.5250**	**-0.0121**	**1**	**36**	**0.063**	**1**	**K.IRDWYQR.Q**
**22**	**519.2715**	**1036.5284**	**1036.5189**	**0.0096**	**0**	**56**	**0.00062**	**1**	**K.YETELNLR.M**
**27**	**553.7750**	**1105.5354**	**1105.5186**	**0.0168**	**0**	**86**	**6.3e-007**	**1**	**K.VTMQNLNDR.L + Oxidation (M)**
**33**	**553.7892**	**1105.5638**	**1105.5550**	**0.0089**	**0**	**57**	**0.00052**	**1**	**R.ISSVLAGGSCR.A**
**34**	**561.7873**	**1121.5600**	**1121.5717**	**-0.0116**	**0**	**62**	**0.00016**	**1**	**R.LEQEIATYR.R**
**37**	**584.3348**	**1166.6550**	**1166.6408**	**0.0143**	**0**	**71**	**1.8e-005**	**1**	**K.VVSTHEQVLR.T**

Query	Observed	Mr(expt)	Mr(calc)	Delta	Miss	Score	Expect	Rank	Peptide
38	586.7756	1171.5366	1171.5186	0.0181	0	24	0.86	1	K.DAEEWFFTK.T
40	593.3602	1184.7058	1184.6877	0.0181	1	73	1.2e-005	1	R.RVLDELTLAR.A
47	610.7772	1219.5398	1219.5932	-0.0533	0	98	4.1e-008	1	K.ASLENSLEETK.G
49	610.8097	1219.6048	1219.5867	0.0182	0	62	0.00016	1	R.MSVEADINGLR.R + Oxidation (M)
60	629.7902	1257.5658	1257.5408	0.0251	0	63	0.00012	1	K.NHEEEMNALR.G + Oxidation (M)
63	422.8939	1265.6599	1265.6615	-0.0017	1	75	7.2e-006	1	R.TKYETELNLR.M
66	638.8353	1275.6560	1275.6380	0.0180	0	77	4.4e-006	1	R.ADLEMQIESLK.E
67	639.7905	1277.5664	1277.5783	-0.0118	0	85	7.6e-007	1	K.GSCGIGGGIGGGSSR.I
70	651.3383	1300.6620	1300.6510	0.0110	0	100	2.1e-008	1	R.ALEEANADLEVK.I
75	454.2398	1359.6976	1359.6929	0.0047	1	46	0.0051	1	R.MSVEADINGLRR.V
81	681.3607	1360.7068	1360.6834	0.0234	0	92	1.5e-007	1	R.EVATNSELVQSGK.S
87	690.3665	1378.7184	1378.7204	-0.0020	1	74	9.4e-006	1	K.TRLEQEIATYR.R
90	713.3462	1424.6778	1424.6896	-0.0117	0	95	6.3e-008	1	R.APSTYGGGLSVSSSR.F
91	478.5821	1432.7245	1432.7157	0.0087	1	78	3e-006	1	K.ASLENSLEETKGR.Y
92	743.7980	1485.5814	1485.5864	-0.0049	0	78	2.9e-006	1	R.CEMEQQNQEYK.I

N° in Table S1	N° spot	protein identification
22 and 44	970	**K5 and K6F and Carnosinase 2**

22 — 1. Q6PI71_HUMAN Mass: 62568 Score: 316 Coverage: 25% — number of peptides : 16

Keratin 5.- Homo sapiens (Human).

Check to include this hit in error tolerant search or archive report

Query	Observed	Mr(expt)	Mr(calc)	Delta	Miss	Score	Expect	Rank	Peptide
3	414.2289	826.4432	826.4225	0.0207	0	35	0.071	1	K.FASFIDK.V
7	442.7400	883.4654	883.4400	0.0255	0	44	0.0083	1	R.TSFTSVSR.S
14	473.2722	944.5298	944.5039	0.0259	1	49	0.0039	1	R.GRLDSELR.N
20	503.2518	1004.4890	1004.4597	0.0294	0	37	0.045	1	K.LLEGEECR.L
22	508.2474	1014.4802	1014.4552	0.0250	0	40	0.027	1	K.HEISEMNR.M
24	508.7881	1015.5616	1015.5298	0.0318	0	41	0.022	1	R.QLDSIVGER.G
26	521.7456	1041.4766	1041.4437	0.0330	0	26	0.68	1	K.DVDAAYMNK.V + Oxidation (M)
33	547.2844	1092.5542	1092.5199	0.0343	0	42	0.016	1	K.AQYEEIANR.S
35	547.8176	1093.6206	1093.5880	0.0327	1	42	0.012	1	R.SLYNLGGSKR.I
40	556.3075	1110.6004	1110.5669	0.0335	0	56	0.0006	1	R.ISISTSGGSFR.N
43	572.3328	1142.6510	1142.6183	0.0328	0	69	2.7e-005	1	K.LAELEEALQK.A
48	586.2881	1170.5616	1170.5227	0.0390	0	37	0.044	1	R.EYQELMNTK.L + Oxidation (M)
52	597.8085	1193.6024	1193.5676	0.0348	0	56	0.00057	1	K.YEELQQTAGR.H
53	602.3398	1202.6650	1202.6295	0.0355	0	37	0.049	1	K.WTLLQEQGTK.T
56	627.8043	1253.5940	1253.5598	0.0342	0	69	2.9e-005	1	R.NMQDLVEDFK.N + Oxidation (M)
63	713.8899	1425.7652	1425.7173	0.0479	1	37	0.037	1	R.TTAENEFVMLKK.D + Oxidation (M)

K2C6F_HUMAN Mass: 60184 Score: 105 Coverage: 10% — number of peptides : 7 with 2 specific for K6 in comparison with K5

22 — Keratin, type II cytoskeletal 6F (Cytokeratin 6F) (CK 6F) (K6f keratin).- Homo sapiens (Human).

Check to include this hit in error tolerant search or archive report

Query	Observed	Mr(expt)	Mr(calc)	Delta	Miss	Score	Expect	Rank	Peptide
3	414.2289	826.4432	826.4225	0.0207	0	35	0.071	1	K.FASFIDK.V
14	473.2722	944.5298	944.5039	0.0259	1	49	0.0039	1	R.GRLDSELR.N
20	503.2518	1004.4890	1004.4597	0.0294	0	37	0.045	1	K.LLEGEECR.L
26	521.7456	1041.4766	1041.4437	0.0330	0	26	0.68	1	K.DVDAAYMNK.V + Oxidation (M)
38	554.2915	1106.5684	1106.5356	0.0328	0	47	0.0048	1	K.AQYEEIAQR.S
49	590.3232	1178.6318	1178.5931	0.0387	0	55	0.00081	1	K.YEELQITAGR.H
53	602.3398	1202.6650	1202.6295	0.0355	0	37	0.049	1	K.WTLLQEQGTK.T

CAD56843 Mass: 53187 Score: 84 Coverage: 7%

Carnosinase 2.- Homo sapiens (Human). — number of peptides : 3

Check to include this hit in error tolerant search or archive report

44

Query	Observed	Mr(expt)	Mr(calc)	Delta	Miss	Score	Expect	Rank	Peptide
27	526.7520	1051.4894	1051.4570	0.0324	0	29	0.25	1	K.YIDENQDR.Y
36	548.7706	1095.5266	1095.4940	0.0326	0	53	0.001	1	R.MMEVAAADVK.Q + 2 Oxidation (M)
41	556.8236	1111.6326	1111.5986	0.0341	0	46	0.0054	1	K.TGQEIPVNVR.F

N° in Table S1	N° spot	protein identification
23 and 46	882	**K6A and D-3-phosphoglycerate dehydrogenase**

1. KRHUEA Mass: 60293 Score: 1259 Coverage: 44% — number of peptides : 23

23 — keratin 6a, type II - human

Check to include this hit in error tolerant search or archive report

Query	Observed	Mr(expt)	Mr(calc)	Delta	Miss	Score	Expect	Rank	Peptide
1	414.2136	826.4126	826.4225	-0.0099	0	36	0.054	1	K.FASFIDK.V

5	453.7297	905.4448	905.4607	-0.0158	0	28	0.45	1	R.FLEQQNK.V
10	473.2498	944.4850	944.5039	-0.0189	1	44	0.012	1	R.GRLDSELR.G
11	483.2448	964.4750	964.4839	-0.0088	1	51	0.0025	1	R.RGFSANSAR.L
12	486.7536	971.4926	971.5036	-0.0109	0	65	8.5e-005	1	K.QEIAEINR.M
15	503.2326	1004.4506	1004.4597	-0.0090	0	41	0.021	1	K.LLEGEECR.L
17	506.7518	1011.4890	1011.4985	-0.0095	0	84	8.7e-007	1	R.SGFSSVSVSR.S
19	508.7670	1015.5194	1015.5298	-0.0104	0	41	0.021	1	R.QLDSIVGER.G
26	533.7540	1065.4934	1065.5090	-0.0156	1	46	0.0054	1	K.YEDEINKR.T
30	554.2653	1106.5160	1106.5356	-0.0196	0	42	0.015	1	K.AQYEEIAQR.S
31	567.2774	1132.5402	1132.5546	-0.0144	1	53	0.0012	1	R.KLLEGEECR.L
33	577.2772	1152.5398	1152.5485	-0.0086	0	55	0.00069	1	K.EYQELMNVK.L
36	583.2896	1164.5646	1164.5775	-0.0128	0	72	1.6e-005	1	K.YEELQVTAGR.H
45	585.7710	1169.5274	1169.5387	-0.0112	1	46	0.0053	1	K.KDVDAAYMNK.V + Oxidation (M)
47	599.2723	1196.5300	1196.5383	-0.0083	0	64	8.6e-005	1	R.GMQDLVEDFK.N + Oxidation (M)
51	602.3169	1202.6192	1202.6295	-0.0103	0	49	0.0031	1	K.WTLLQEQGTK.T
53	611.8150	1221.6154	1221.6241	-0.0087	0	78	3e-006	1	R.TAAENEFVTLK.K
63	675.8615	1349.7084	1349.7191	-0.0106	1	116	6.4e-010	1	R.TAAENEFVTLKK.D
66	679.3645	1356.7144	1356.7249	-0.0104	1	113	1.2e-009	1	K.NKLEGLEDALQK.A
68	712.7966	1423.5786	1423.6263	-0.0476	0	134	7.9e-012	1	R.GSGGLGGACGGAGFGSR.S
70	720.3424	1438.6702	1438.6762	-0.0060	1	76	4e-006	1	R.GMQDLVEDFKNK.Y + Oxidation (M)
71	724.3814	1446.7482	1446.7678	-0.0196	0	114	7.6e-010	1	R.AIGGGLSSVGGGSSTIK.Y
74	508.6021	1522.7845	1522.8065	-0.0220	1	32	0.11	1	R.LLKEYQELMNVK.L + Oxidation (M)

46 SERA_HUMAN Mass: 57225 Score: 269 Coverage: 12%
D-3-phosphoglycerate dehydrogenase (EC 1.1.1.95) (3-PGDH).- Homo sapiens (Human). number of peptides : 6
Check to include this hit in error tolerant search or archive report

	Query	Observed	Mr(expt)	Mr(calc)	Delta	Miss	Score	Expect	Rank	Peptide
☑	3	450.2813	898.5480	898.5600	-0.0120	0	38	0.035	1	K.TLGILGLGR.I
☑	16	506.7376	1011.4606	1011.4695	-0.0089	0	35	0.067	1	K.FMGTELNGK.T + Oxidation (M)
☑	27	536.2872	1070.5598	1070.5720	-0.0122	0	88	4e-007	1	K.QADVNLVNAK.L
☑	28	550.3054	1098.5962	1098.6033	-0.0071	0	58	0.00032	1	R.GGIVDEGALLR.A
☑	61	673.3832	1344.7518	1344.7613	-0.0094	0	70	2.1e-005	1	K.GTIQVITQGTSLK.N
☑	73	744.8636	1487.7126	1487.7216	-0.0090	0	127	3.7e-011	1	R.AGTGVDNVDLEAATR.K

N° in Table S1	N° spot	protein identification
**24**	**870**	**K6A**

1. KRHUEA Mass: 60293 Score: 662 Coverage: 34% number of peptides : 19
keratin 6a, type II - human
Check to include this hit in error tolerant search or archive report

Query	Observed	Mr(expt)	Mr(calc)	Delta	Miss	Score	Expect	Rank	Peptide
2	414.2411	826.4676	826.4225	0.0451	0	38	0.039	1	K.FASFIDK.V
12	473.2849	944.5552	944.5039	0.0513	1	42	0.019	1	R.GRLDSELR.G
17	486.7865	971.5584	971.5036	0.0549	0	51	0.0021	1	K.QEIAEINR.M
18	503.2638	1004.5130	1004.4597	0.0534	0	39	0.03	1	K.LLEGEECR.L
21	506.7849	1011.5552	1011.4985	0.0567	0	95	6.3e-008	1	R.SGFSSVSVSR.S
23	508.8010	1015.5874	1015.5298	0.0576	0	50	0.003	1	R.QLDSIVGER.G
28	521.7582	1041.5018	1041.4437	0.0582	0	35	0.075	1	K.DVDAAYMNK.V + Oxidation (M)
30	533.7916	1065.5686	1065.5090	0.0596	1	46	0.0055	1	K.YEDEINKR.T
31	541.8350	1081.6554	1081.5920	0.0634	1	28	0.31	1	K.FASFIDKVR.F
38	567.3174	1132.6202	1132.5546	0.0656	1	48	0.0039	1	R.KLLEGEECR.L
39	577.3153	1152.6160	1152.5485	0.0676	0	42	0.015	1	K.EYQELMNVK.L
42	583.3296	1164.6446	1164.5775	0.0672	0	58	0.00035	1	K.YEELQVTAGR.H
48	599.3125	1196.6104	1196.5383	0.0721	0	56	0.0005	1	R.GMQDLVEDFK.N + Oxidation (M)
51	602.3565	1202.6984	1202.6295	0.0689	0	54	0.00088	1	K.WTLLQEQGTK.T
53	611.8542	1221.6938	1221.6241	0.0697	0	87	3.9e-007	1	R.TAAENEFVTLK.K
59	450.9363	1349.7871	1349.7191	0.0680	1	72	1.7e-005	1	R.TAAENEFVTLKK.D
61	679.4083	1356.8020	1356.7249	0.0772	1	33	0.1	1	K.NKLEGLEDALQK.A
63	712.8631	1423.7116	1423.6263	0.0854	0	108	2.7e-009	1	R.GSGGLGGACGGAGFGSR.S
64	724.4338	1446.8530	1446.7678	0.0852	0	102	1.3e-008	1	R.AIGGGLSSVGGGSSTIK.Y

N° in Table S1	N° spot	protein identification
**25**	**862**	**K6A**

1. Q96CL4_HUMAN Mass: 60266 Score: 427 Coverage: 29% number of peptides : 15

Keratin 6A.- Homo sapiens (Human).
Check to include this hit in error tolerant search or archive report

Query	Observed	Mr(expt)	Mr(calc)	Delta	Miss	Score	Expect	Rank	Peptide
8	453.7573	905.5000	905.4607	0.0394	0	28	0.44	1	R.FLEQQNK.V
9	473.2813	944.5480	944.5039	0.0441	1	44	0.012	1	R.GRLDSELR.G
12	486.7813	971.5480	971.5036	0.0445	0	46	0.0063	1	K.QEIAEINR.M
14	503.2612	1004.5078	1004.4597	0.0482	0	37	0.048	1	K.LLEGEECR.L
15	506.7801	1011.5456	1011.4985	0.0471	0	80	2.1e-006	1	R.SGFSSVSVSR.S
16	508.7969	1015.5792	1015.5298	0.0494	0	49	0.0035	1	R.QLDSIVGER.G
18	521.7522	1041.4898	1041.4437	0.0462	0	30	0.24	1	K.DVDAAYMNK.V + Oxidation (M)
21	554.2993	1106.5840	1106.5356	0.0484	0	34	0.087	1	K.AQYEEIAQR.S
25	583.3248	1164.6350	1164.5775	0.0576	0	50	0.0021	1	K.YEELQVTAGR.H
26	585.3054	1168.5962	1168.5434	0.0529	0	34	0.085	1	K.EYQELMNVK.L + Oxidation (M)
29	591.3089	1180.6032	1180.5434	0.0598	0	46	0.005	1	R.GMQDLVEDFK.N
33	602.3510	1202.6874	1202.6295	0.0579	0	44	0.01	1	K.WTLLQEQGTK.T
38	712.8575	1423.7004	1423.6263	0.0742	0	81	1.5e-006	1	R.GSGGLGGACGGAGFGSR.S
40	724.4305	1446.8464	1446.7678	0.0786	0	70	2.2e-005	1	R.AIGGGLSSVGGGSSTIK.Y
42	799.9243	1597.8340	1597.7519	0.0822	0	121	1.5e-010	1	R.ISIGGGSCAISGGYGSR.A

N° in Table S1 N° spot protein identification

26 and 47 **871** **K6A and D-3-phosphoglycerate dehydrogenase**

Q96CL4_HUMAN Mass: 60266 Score: 602 Coverage: 32% number of peptides : 19

26 Keratin 6A.- Homo sapiens (Human).
Check to include this hit in error tolerant search or archive report

Query	Observed	Mr(expt)	Mr(calc)	Delta	Miss	Score	Expect	Rank	Peptide
2	414.2170	826.4194	826.4225	-0.0031	0	38	0.034	1	K.FASFIDK.V
13	473.2594	944.5042	944.5039	0.0003	1	37	0.059	1	R.GRLDSELR.G
16	486.7575	971.5004	971.5036	-0.0031	0	43	0.012	1	K.QEIAEINR.M
19	503.2367	1004.4588	1004.4597	-0.0008	0	54	0.00093	1	K.LLEGEECR.L
21	506.7544	1011.4942	1011.4985	-0.0043	0	77	4.5e-006	1	R.SGFSSVSVSR.S
23	508.7714	1015.5282	1015.5298	-0.0016	0	38	0.043	1	R.QLDSIVGER.G
26	521.7274	1041.4402	1041.4437	-0.0034	0	33	0.12	1	K.DVDAAYMNK.V + Oxidation (M)
29	533.7592	1065.5038	1065.5090	-0.0052	1	46	0.0057	1	K.YEDEINKR.T
35	554.2739	1106.5332	1106.5356	-0.0024	0	48	0.004	1	K.AQYEEIAQR.S
44	583.2949	1164.5752	1164.5775	-0.0022	0	58	0.00036	1	K.YEELQVTAGR.H
46	585.2784	1168.5422	1168.5434	-0.0011	0	41	0.02	1	K.EYQELMNVK.L + Oxidation (M)
48	599.2766	1196.5386	1196.5383	0.0003	0	66	5e-005	1	R.GMQDLVEDFK.N + Oxidation (M)
50	602.3235	1202.6324	1202.6295	0.0029	0	49	0.0028	1	K.WTLLQEQGTK.T
51	611.8188	1221.6230	1221.6241	-0.0011	0	87	4e-007	1	R.TAAENEFVTLK.K
58	658.3523	1314.6900	1314.6891	0.0009	1	84	8.8e-007	1	R.NTKQEIAEINR.M
62	450.9080	1349.7022	1349.7191	-0.0169	1	69	3.9e-005	1	R.TAAENEFVTLKK.D
63	679.3706	1356.7266	1356.7249	0.0018	1	33	0.11	1	K.NKLEGLEDALQK.A
64	712.8226	1423.6306	1423.6263	0.0044	0	88	2.9e-007	1	R.GSGGLGGACGGAGFGSR.S
67	724.3908	1446.7670	1446.7678	-0.0008	0	95	6.3e-008	1	R.AIGGGLSSVGGGSSTIK.Y

SERA_HUMAN Mass: 57225 Score:188 Coverage: 14% number of peptides : 6
D-3-phosphoglycerate dehydrogenase (EC 1.1.1.95) (3-PGDH).- Homo sapiens (Human).
Check to include this hit in error tolerant search or archive report

47

Query	Observed	Mr(expt)	Mr(calc)	Delta	Miss	Score	Expect	Rank	Peptide
31	536.2927	1070.5708	1070.5720	-0.0012	0	83	1.4e-006	1	K.QADVNLVNAK.L
32	545.7814	1089.5482	1089.5488	-0.0006	0	25	0.77	1	R.QIPQATASMK.D + Oxidation (M)
33	550.3076	1098.6006	1098.6033	-0.0027	0	26	0.57	1	R.GGIVDEGALLR.A
39	565.8051	1129.5956	1129.5979	-0.0022	0	54	0.00099	1	K.VTADVINAAEK.L
55	649.8688	1297.7230	1297.7242	-0.0011	0	102	1.1e-008	1	K.ILQDGGLQVVEK.Q
61	673.3870	1344.7594	1344.7613	-0.0018	0	33	0.12	1	K.GTIQVITQGTSLK.N

N° in Table S1 N° spot protein identification

27 **1525** **Stathmin**

1. STMN1_HUMAN Mass: 17161 Score: 79 Coverage: 20% number of peptides : 3
Stathmin (Phosphoprotein p19) (pp19) (Oncoprotein 18) (Op18) (Leukemia-associated phosphoprotein p1
Check to include this hit in error tolerant search or archive report

Query	Observed	Mr(expt)	Mr(calc)	Delta	Miss	Score	Expect	Rank	Peptide
8	537.8100	1073.6054	1073.5604	0.0450	0	38	0.043	1	K.DLSLEEIQK.K
10	583.2971	1164.5796	1164.5411	0.0386	0	59	0.00028	1	K.AIEENNNFSK.M
13	653.7860	1305.5574	1305.5208	0.0366	1	44	0.0084	1	K.ESKDPADETEAD.-

N° in Table S1	N° spot	protein identification
28	292	**dynactin 1**

1. AAD03694 Mass: 140419 Score: 343 Coverage: 9% number of peptides : 12
HSDCTN22 NID: - Homo sapiens
Check to include this hit in error tolerant search or archive report

Query	Observed	Mr(expt)	Mr(calc)	Delta	Miss	Score	Expect	Rank	Peptide
5	445.2356	888.4566	888.4552	0.0014	0	37	0.068	1	R.ETELELR.E
6	493.7660	985.5174	985.5192	-0.0018	0	47	0.0052	1	K.NQELEVVR.Q
8	503.7332	1005.4518	1005.4549	-0.0031	0	38	0.043	1	R.EQLDMAGAR.V + Oxidation (M)
9	536.8028	1071.5910	1071.5924	-0.0013	0	63	0.00014	1	R.LEETQALLR.K
11	592.2841	1182.5536	1182.5517	0.0020	0	90	1.8e-007	1	K.GSDGAASSYQLK.Q
13	598.2677	1194.5208	1194.5187	0.0022	0	46	0.0055	1	R.DLETSCSDIR.Q
16	608.8252	1215.6358	1215.6347	0.0012	0	56	0.00063	1	R.AEITDAEGLGLK.L
17	631.7936	1261.5726	1261.5721	0.0006	0	68	3.9e-005	1	K.MQEQQADLQR.R + Oxidation (M)
18	432.2310	1293.6712	1293.6789	-0.0078	0	47	0.0042	1	R.QLTAHLQDVNR.E
20	448.9045	1343.6917	1343.7045	-0.0128	1	23	0.92	1	K.IKGEELSEANVR.L
22	702.3417	1402.6688	1402.6688	0.0000	0	92	1.1e-007	1	R.ELTNQQEASVER.Q
23	712.8510	1423.6874	1423.6799	0.0075	0	72	1.2e-005	1	R.QSCNILISTMNK.L + Oxidation (M)

N° in Table S1	N° spot	protein identification
29	418	**Coronin-7**

BAB15211 Mass: 101596 Score: 56 Coverage: 1% number of peptides : 1
AK025674 NID: - Homo sapiens (Coronin-7)
Check to include this hit in error tolerant search or archive report

Query	Observed	Mr(expt)	Mr(calc)	Delta	Miss	Score	Expect	Rank	Peptide
5	646.8463	1291.6780	1291.6408	0.0372	0	56	0.00057	1	R.SGPEPLQEGPGPK.G

N° in Table S1	N° spot	protein identification
30	1302	**Hsp-27 Phospho S82**

1. HHHU27 Mass: 22826 Score:375 Coverage: 24% number of peptides : 6
heat shock protein 27 - human
Check to include this hit in error tolerant search or archive report

Query	Observed	Mr(expt)	Mr(calc)	Delta	Miss	Score	Expect	Rank	Peptide
9	471.3003	940.5860	940.4978	0.0883	0	33	0.23	1	R.AQLGGPEAAK.S
20	552.8088	1103.6030	1103.4996	0.1035	0	61	0.00034	1	R.QDEHGYISR.C
22	573.8721	1145.7296	1145.6292	0.1004	1	65	0.00013	1	K.TKDGVVEITGK.H
23	578.3216	1154.6286	1154.5332	0.0954	0	71	4e-005	1	R.QLSSGVSEIR.H + Phospho (ST)
33	490.2900	1467.8482	1467.7318	0.1164	1	70	4.4e-005	1	K.DGVVEITGKHEER.Q
34	548.6583	1642.9531	1642.8162	0.1369	1	116	9.7e-010	1	R.AQLGGPEAAKSDETAAK.-

N° in Table S1	N° spot	protein identification
31	239	**Osmotic stress protein 94**

1. HS74L_HUMAN Mass: 95453 Score: 593 Coverage: 14% number of peptides : 11
Heat shock 70 kDa protein 4L (Osmotic stress protein 94) (Heat shock 70-related protein APG-1).- Ho
Check to include this hit in error tolerant search or archive report

Query	Observed	Mr(expt)	Mr(calc)	Delta	Miss	Score	Expect	Rank	Peptide
1	405.2249	808.4352	808.4331	0.0022	0	25	0.69	1	R.LGTVYEK.F
5	449.2319	896.4492	896.4504	-0.0012	0	36	0.048	1	K.NHPAPFSK.V
6	458.7638	915.5130	915.5138	-0.0007	0	46	0.0065	1	K.SQIVTNVR.N
12	506.7690	1011.5234	1011.5237	-0.0002	0	45	0.0067	1	K.AEVPEDKPK.A
13	525.2799	1048.5452	1048.5441	0.0012	0	49	0.003	1	K.FITPEDLSK.L
15	550.3216	1098.6286	1098.6285	0.0002	0	68	3.7e-005	1	K.LSLTQDPVVK.V
17	566.8144	1131.6142	1131.6135	0.0007	1	46	0.0063	1	K.LKETSENALK.K
20	588.2949	1174.5752	1174.5764	-0.0012	0	57	0.00046	1	K.AVMEQANLQR.E + Oxidation (M)
22	611.2888	1220.5630	1220.5642	-0.0011	0	36	0.065	1	R.CTPACISLGSR.T
24	653.8178	1305.6210	1305.6201	0.0009	0	45	0.0056	1	R.SFDDPIVQTER.I
25	738.3699	1474.7252	1474.7263	-0.0011	0	85	6e-007	1	K.DISTTLNADEAVAR.G

N° in Table S1	N° spot	protein identification
32	1319	**Glutathione S-transferase Mu 3**

1. GSTM3_HUMAN Mass: 26867 Score: 184 Coverage: 22% number of peptides : 6
Glutathione S-transferase Mu 3 (EC 2.5.1.18) (GSTM3-3) (GST class-mu 3) (hGSTM3-3).- Homo sapiens (
Check to include this hit in error tolerant search or archive report

Query	Observed	Mr(expt)	Mr(calc)	Delta	Miss	Score	Expect	Rank	Peptide
6	508.3033	1014.5920	1014.5822	0.0099	0	67	4.7e-005	1	K.ITQSNAILR.Y
8	568.2872	1134.5598	1134.5379	0.0219	0	43	0.013	1	K.CLDEFPNLK.A
9	569.7542	1137.4938	1137.4761	0.0178	0	31	0.17	1	R.LCYSSDHEK.L
13	610.7866	1219.5586	1219.5478	0.0109	0	48	0.0037	1	K.MAQWGNKPIC.- + Oxidation (M)

Query	Observed	Mr(expt)	Mr(calc)	Delta	Miss	Score	Expect	Rank	Peptide
14	629.3758	1256.7370	1256.7200	0.0170	1	38	0.038	1	K.NKITQSNAILR.Y
16	673.7784	1345.5422	1345.5245	0.0178	0	59	0.00029	1	R.YTCGEAPDYDR.S

N° in Table S1	N° spot	protein identification
33	699	**HSP70**

1. AAD21815 Mass: 70267 Score: 159 Coverage: 7% number of peptides : 6
AF134726 NID: - Homo sapiens
Check to include this hit in error tolerant search or archive report

Query	Observed	Mr(expt)	Mr(calc)	Delta	Miss	Score	Expect	Rank	Peptide
1	402.7232	803.4318	803.4389	-0.0070	0	41	0.024	1	K.ITITNDK.G
3	502.2713	1002.5280	1002.5345	-0.0065	1	63	0.00013	1	R.LSKEEIER.M
4	509.2865	1016.5584	1016.5614	-0.0030	1	50	0.0029	1	K.ITITNDKGR.L
5	566.7945	1131.5744	1131.5771	-0.0027	1	62	0.00014	1	K.SAVEDEGLKGK.I
6	569.2785	1136.5424	1136.5462	-0.0037	1	54	0.00088	1	K.YKAEDEVQR.E
8	569.8091	1137.6036	1137.6030	0.0007	1	36	0.05	1	K.VQVSYKGETK.A

N° in Table S1	N° spot	protein identification
34	1480	**glutathione S transferase P**

1. GTP_HUMAN Mass: 23438 Score: 55 Coverage: 4% number of peptides : 1
Glutathione S-transferase P (EC 2.5.1.18) (GST class-pi) (GSTP1-1).- Homo sapiens (Human).
Check to include this hit in error tolerant search or archive report

Query	Observed	Mr(expt)	Mr(calc)	Delta	Miss	Score	Expect	Rank	Peptide
4	568.8204	1135.6262	1135.5696	0.0567	0	55	0.00076	1	K.ASCLYGQLPK.F

N° in Table S1	N° spot	protein identification
35	539	**TRIM29**

1. AAH17352 Mass: 66478 Score:366 Coverage: 22% number of peptides : 12
BC017352 NID: - Homo sapiens
Check to include this hit in error tolerant search or archive report

Query	Observed	Mr(expt)	Mr(calc)	Delta	Miss	Score	Expect	Rank	Peptide
8	495.7635	989.5124	989.5294	-0.0170	0	39	0.038	1	K.AILEQNFR.D
10	508.7827	1015.5508	1015.5702	-0.0194	0	41	0.019	1	K.KPPVTFAEK.G
11	509.7388	1017.4630	1017.4841	-0.0211	0	46	0.007	1	R.YSMYLTPK.G + Oxidation (M)
12	516.2442	1030.4738	1030.4931	-0.0193	0	30	0.22	1	R.EQDAVDQVK.V
15	535.7573	1069.5000	1069.5192	-0.0192	0	31	0.16	1	K.NFNNLYGTK.G
16	539.2542	1076.4938	1076.5172	-0.0233	0	58	0.00042	1	K.VIMDALDER.A + Oxidation (M)
17	540.2512	1078.4878	1078.5043	-0.0165	0	40	0.022	1	R.TSYQPSSPGR.F
19	586.3134	1170.6122	1170.6357	-0.0234	0	62	0.00014	1	K.SLGSALKPGEGR.S
20	588.3176	1174.6206	1174.6346	-0.0140	0	69	3.1e-005	1	R.SPYAGLQLGAAK.K
23	659.3086	1316.6026	1316.6208	-0.0182	0	88	3.5e-007	1	R.SPSGPSGSLENGTK.A
28	487.5707	1459.6903	1459.7341	-0.0438	0	44	0.0071	1	R.KPTVSIMEPGETR.R + Oxidation (M)
30	827.8486	1653.6826	1653.6974	-0.0148	0	80	1.6e-006	1	K.SGSEEVLCDSCIGNK.Q

N° in Table S1	N° spot	protein identification
36	525	**TRIM29**

1. AAH17352 Mass: 66478 Score: 304 Coverage: 20% number of peptides : 11
BC017352 NID: - Homo sapiens
Check to include this hit in error tolerant search or archive report

Query	Observed	Mr(expt)	Mr(calc)	Delta	Miss	Score	Expect	Rank	Peptide
6	433.7544	865.4942	865.4294	0.0649	0	51	0.0018	1	R.ADTGLFSR.S
8	477.2958	952.5770	952.4978	0.0793	1	38	0.034	1	R.KSIFSESR.K
11	495.8099	989.6052	989.5294	0.0758	0	35	0.08	1	K.AILEQNFR.D
15	516.2980	1030.5814	1030.4931	0.0883	0	30	0.26	1	R.EQDAVDQVK.V
22	535.8091	1069.6036	1069.5192	0.0844	0	39	0.029	1	K.NFNNLYGTK.G
24	539.3118	1076.6090	1076.5172	0.0919	0	57	0.00056	1	K.VIMDALDER.A + Oxidation (M)
25	540.3068	1078.5990	1078.5043	0.0947	0	52	0.0014	1	R.TSYQPSSPGR.F
29	586.3747	1170.7348	1170.6357	0.0992	0	41	0.017	1	K.SLGSALKPGEGR.S
31	588.3700	1174.7254	1174.6346	0.0909	0	61	0.00019	1	R.SPYAGLQLGAAK.K
38	638.3973	1274.7800	1274.6718	0.1083	1	97	4.3e-008	1	K.AEKETELSLQK.E
44	730.9382	1459.8618	1459.7341	0.1278	0	40	0.017	1	R.KPTVSIMEPGETR.R + Oxidation (M)

N° in Table S1	N° spot	protein identification
37	532	**TRIM29**

1. AAH17352 Mass: 66478 Score: 325 Coverage: 23% number of peptides : 13
BC017352 NID: - Homo sapiens
Check to include this hit in error tolerant search or archive report

Query	Observed	Mr(expt)	Mr(calc)	Delta	Miss	Score	Expect	Rank	Peptide

Query	Observed	Mr(expt)	Mr(calc)	Delta	Miss	Score	Expect	Rank	Peptide
11	495.7802	989.5458	989.5294	0.0164	0	35	0.081	1	K.AILEQNFR.D
13	508.7993	1015.5840	1015.5702	0.0138	0	57	0.00059	1	K.KPPVTFAEK.G
14	509.7560	1017.4974	1017.4841	0.0133	0	37	0.051	1	R.YSMYLTPK.G + Oxidation (M)
19	534.2936	1066.5726	1066.5659	0.0068	1	47	0.0067	1	R.IKSFTTNEK.A
20	535.7772	1069.5398	1069.5192	0.0206	0	24	0.94	1	K.NFNNLYGTK.G
21	539.2730	1076.5314	1076.5172	0.0143	0	58	0.00043	1	K.VIMDALDER.A + Oxidation (M)
22	540.2622	1078.5098	1078.5043	0.0055	0	47	0.0042	1	R.TSYQPSSPGR.F
24	575.7990	1149.5834	1149.5567	0.0268	0	38	0.033	1	R.SALFAGNEWR.R
25	586.3337	1170.6528	1170.6357	0.0172	0	62	0.00013	1	K.SLGSALKPGEGR.S
26	588.3377	1174.6608	1174.6346	0.0262	0	74	9.4e-006	1	R.SPYAGLQLGAAK.K
32	730.8862	1459.7578	1459.7341	0.0238	0	47	0.0037	1	R.KPTVSIMEPGETR.R + Oxidation (M)
33	827.8808	1653.7470	1653.6974	0.0496	0	70	1.7e-005	1	K.SGSEEVLCDSCIGNK.Q
34	567.2976	1698.8710	1698.8537	0.0173	1	23	0.8	1	R.AALEQREQDAVDQVK.V

N° in Table S1 N° spot protein identification

38 445 DNA mismatch repair protein

I64819 Mass: 105418 Score: 115 Coverage: 3% number of peptides : 4

DNA mismatch repair protein - human

Check to include this hit in error tolerant search or archive report

Query	Observed	Mr(expt)	Mr(calc)	Delta	Miss	Score	Expect	Rank	Peptide
6	489.2871	976.5596	976.5593	0.0003	0	47	0.0052	1	K.IIQEFLSK.V
12	547.2869	1092.5592	1092.5597	-0.0005	0	45	0.0066	1	K.MQSTLISAAR.D + Oxidation (M)
14	558.3027	1114.5908	1114.5982	-0.0074	0	59	0.00027	1	R.QTLQEDLLR.R
18	599.3032	1196.5918	1196.5925	-0.0006	0	57	0.00044	1	K.LTSLNEEYTK.N

N° in Table S1 N° spot protein identification

39 and 69 967 PAI-2 and/or GA17 protein

39 AAA36413 Mass: 46939 Score: 769 Coverage: 29% number of peptides : 11

HUMPAI2 NID: - Homo sapiens

1. Check to include this hit in error tolerant search or archive report

Query	Observed	Mr(expt)	Mr(calc)	Delta	Miss	Score	Expect	Rank	Peptide
10	499.7111	997.4076	997.4287	-0.0211	0	65	6.2e-005	1	R.ANFSGMSER.N
15	544.7617	1087.5088	1087.5298	-0.0209	0	42	0.014	1	K.LEEHYELR.S
19	554.3125	1106.6104	1106.6236	-0.0132	1	54	0.001	1	K.KLNGLYPFR.V
29	573.2240	1144.4334	1144.4529	-0.0194	0	58	0.00038	1	R.SMGMEDAFNK.G + Oxidation (M)
39	585.7743	1169.5340	1169.5573	-0.0232	0	46	0.0045	1	R.TPVQMMYLR.E + 2 Oxidation (M)
44	589.3212	1176.6278	1176.6390	-0.0112	0	66	5.2e-005	1	K.LNIGYIEDLK.A
48	600.3218	1198.6290	1198.6420	-0.0130	0	66	5.9e-005	1	R.MVLVNAVYFK.G + Oxidation (M)
55	419.8683	1256.5831	1256.6149	-0.0318	1	55	0.00071	1	K.SASFREEYIR.L
61	458.8525	1373.5357	1373.5704	-0.0347	1	62	0.00014	1	R.SMGMEDAFNKGR.A + 2 Oxidation (M)
68	774.3855	1546.7564	1546.7627	-0.0063	0	77	4.1e-006	1	K.GSYPDAILQAQAADK.I
70	791.9081	1581.8016	1581.7999	0.0018	0	53	0.00099	1	K.IPNLLPEGSVDGDTR.M

69 Q96KM8_HUMAN Mass: 42932 Score: 133 Coverage: 14% number of peptides : 4

DJ69B10.1 (GA17 protein) (Dendritic cell protein).- Homo sapiens (Human).

2. Check to include this hit in error tolerant search or archive report

Query	Observed	Mr(expt)	Mr(calc)	Delta	Miss	Score	Expect	Rank	Peptide
31	573.7905	1145.5664	1145.5791	-0.0126	0	29	0.26	1	R.YTVYCSLIK.V
52	416.2153	1245.6241	1245.6578	-0.0337	1	38	0.035	1	K.FREGERPSLR.L
60	451.2396	1350.6970	1350.7217	-0.0247	1	40	0.018	1	R.LLYEALVDCKK.S
63	693.3398	1384.6650	1384.6731	-0.0080	0	83	1.1e-006	1	R.LLTFMGMAVENK.E + 2 Oxidation (M)

N° in Table S1 N° spot protein identification

40 1118 PAI-2

1. 1BY7A Mass: 40607 Score: 82 Coverage: 7% number of peptides : 3

plasminogen activator inhibitor-2 mutant RESIDUES 66 98 EXCISED - human (fragments)

Check to include this hit in error tolerant search or archive report

Query	Observed	Mr(expt)	Mr(calc)	Delta	Miss	Score	Expect	Rank	Peptide
13	544.7851	1087.5556	1087.5298	0.0259	0	50	0.0025	1	K.LEEHYELR.S
14	581.2483	1160.4820	1160.4478	0.0342	0	43	0.012	1	R.SMGMEDAFNK.G + 2 Oxidation (M)
15	585.8010	1169.5874	1169.5573	0.0302	0	38	0.031	1	R.TPVQMMYLR.E + 2 Oxidation (M)

N° in Table S1 N° spot protein identification

41 1001 PAI-2

A32853 Mass: 46851 Score: 561 Coverage: 19% number of peptides : 10

plasminogen activator inhibitor 2 precursor - human
Check to include this hit in error tolerant search or archive report

Query	Precursor mass Observed	Mr(expt)	Mr(calc)	mass error observed Delta	Miss	Score	Expect	Rank	peptide sequence Peptide
6	468.2933	934.5720	934.4912	0.0808	1	32	0.2	1	K.WKTPFEK.K
10	490.3109	978.6072	978.5287	0.0786	0	37	0.055	1	K.LNGLYPFR.V
15	507.7589	1013.5032	1013.4236	0.0796	0	57	0.00053	1	R.ANFSGMSER.N + Oxidation (M)
23	544.8186	1087.6226	1087.5298	0.0929	0	46	0.0057	1	K.LEEHYELR.S
27	554.3665	1106.7184	1106.6236	0.0948	1	56	0.00063	1	K.KLNGLYPFR.V
34	573.2828	1144.5510	1144.4529	0.0982	0	66	6.1e-005	1	R.SMGMEDAFNK.G + Oxidation (M)
36	577.8364	1153.6582	1153.5624	0.0959	0	48	0.0035	1	R.TPVQMMYLR.E + Oxidation (M)
51	600.3798	1198.7450	1198.6420	0.1030	0	53	0.0011	1	R.MVLVNAVYFK.G + Oxidation (M)
59	458.8971	1373.6695	1373.5704	0.0991	1	57	0.00036	1	R.SMGMEDAFNKGR.A + 2 Oxidation (M)
63	774.4555	1546.8964	1546.7627	0.1337	0	80	1.7e-006	1	K.GSYPDAILQAQAADK.I

N° in Table S1	N° spot	protein identification
42 and 45	**1145**	**Activator of 90 kDa heat shock protein ATPase homolog 1 (p38K protein) and/or PAI-2**

1. JC7769 Mass: 38421 Score: 281 Coverage: 24% number of peptides : 10

45 38K protein - human
Check to include this hit in error tolerant search or archive report

Query	Observed	Mr(expt)	Mr(calc)	Delta	Miss	Score	Expect	Rank	Peptide
8	416.2300	830.4454	830.4286	0.0168	0	38	0.047	1	R.WIVEER.A
14	450.2289	898.4432	898.4297	0.0135	0	41	0.018	1	K.QTFGYGAR.L
16	453.7452	905.4758	905.4607	0.0152	0	61	0.00022	1	K.LNWTGTSK.S
22	476.2354	950.4562	950.4379	0.0184	0	31	0.19	1	K.CEVTEVSK.L
25	499.2602	996.5058	996.4876	0.0183	0	49	0.003	1	R.GIPAPEEER.T
33	544.7806	1087.5466	1087.5258	0.0209	0	68	4.3e-005	1	K.LDGEASINNR.K
48	608.8310	1215.6474	1215.6207	0.0267	1	36	0.051	1	K.LDGEASINNRK.G
50	621.3438	1240.6730	1240.6373	0.0357	0	58	0.00028	1	R.EAMGIYISTLK.T + Oxidation (M)
52	656.2836	1310.5526	1310.5231	0.0296	0	52	0.0014	1	K.NGETELCMEGR.G + Oxidation (M)
53	681.3518	1360.6890	1360.6544	0.0346	0	78	3.4e-006	1	K.DEPDTNLVALMK.E + Oxidation (M)

1BY7A Mass: 40607 Score: 245 Coverage: 12% number of peptides : 4
plasminogen activator inhibitor-2 mutant RESIDUES 66 98 EXCISED - human (fragments)

2. Check to include this hit in error tolerant search or archive report

42

Query	Observed	Mr(expt)	Mr(calc)	Delta	Miss	Score	Expect	Rank	Peptide
27	507.7288	1013.4430	1013.4236	0.0194	0	56	0.00067	1	R.ANFSGMSER.N + Oxidation (M)
34	544.7822	1087.5498	1087.5298	0.0201	0	53	0.0012	1	K.LEEHYELR.S
39	573.2462	1144.4778	1144.4529	0.0250	0	68	3.3e-005	1	R.SMGMEDAFNK.G + Oxidation (M)
45	585.7980	1169.5814	1169.5573	0.0242	0	43	0.011	1	R.TPVQMMYLR.E + 2 Oxidation (M)

N° in Table S1	N° spot	protein identification
**43**	**1214**	**Maspin**

1. A36898 Mass: 42568 Score: 201 Coverage: 18% number of peptides : 10
maspin - human
Check to include this hit in error tolerant search or archive report

Query	Observed	Mr(expt)	Mr(calc)	Delta	Miss	Score	Expect	Rank	Peptide
8	437.2598	872.5050	872.4716	0.0335	0	27	0.69	1	K.GQINNSIK.D
11	472.7778	943.5410	943.5015	0.0396	0	34	0.11	1	K.LSSFYSLK.L
13	483.2683	964.5220	964.4865	0.0355	1	36	0.07	1	K.KFPESETK.E
16	490.7701	979.5256	979.4862	0.0394	0	47	0.0046	1	K.ELETVDFK.D
19	509.2957	1016.5768	1016.5324	0.0444	0	62	0.00018	1	K.ACLENLGLK.H
29	551.3464	1100.6782	1100.6230	0.0553	0	35	0.067	1	K.IIELPFQNK.H
33	611.3006	1220.5866	1220.5408	0.0458	0	80	2.7e-006	1	K.DVEDESTGLEK.I
34	612.3372	1222.6598	1222.6081	0.0517	1	69	2.9e-005	1	K.ELETVDFKDK.L
33	611.3006	1220.5866	1220.5408	0.0458	0	80	5e-006	1	K.DVEDESTGLEK.I
34	612.3372	1222.6598	1222.6081	0.0518	1	69	5.9e-005	1	K.ELETVDFKDK.L

N° in Table S1	N° spot	protein identification
**44**		see on N°22
**45**		see on N°42
**46**		see on N°23
**47**		see on N°26

N° in Table S1	N° spot	protein identification
**48**	**648**	**protein disulfide-isomerase precursor**

1. ISHUSS Mass: 57480 Score: 318 Coverage: 21% number of peptides : 12
protein disulfide-isomerase (EC 5.3.4.1) precursor - human
Check to include this hit in error tolerant search or archive report

Query	Observed	Mr(expt)	Mr(calc)	Delta	Miss	Score	Expect	Rank	Peptide
1	404.7610	807.5074	807.4491	0.0584	1	35	0.072	1	R.TLDGFKK.F
5	431.7678	861.5210	861.4596	0.0615	0	29	0.35	1	K.ALAPEYAK.A
8	455.7581	909.5016	909.4345	0.0672	0	35	0.082	1	K.FFPASADR.T

Query	Observed	Mr(expt)	Mr(calc)	Delta	Miss	Score	Expect	Rank	Peptide
10	464.8001	927.5856	927.5178	0.0678	0	40	0.023	1	K.VHSFPTLK.F
11	481.7663	961.5180	961.4440	0.0741	0	55	0.00082	1	R.ITEFCHR.F
12	485.8084	969.6022	969.5284	0.0739	0	35	0.065	1	K.QLAPIWDK.L
13	501.8189	1001.6232	1001.5505	0.0727	1	48	0.0043	1	K.LKAEGSEIR.L
14	519.2869	1036.5592	1036.4825	0.0767	0	48	0.0034	1	R.NNFEGEVTK.E
18	579.8451	1157.6756	1157.5829	0.0928	0	41	0.019	1	K.SNFAEALAAHK.Y
19	611.8619	1221.7092	1221.6162	0.0930	0	56	0.0005	1	R.LITLEEEMTK.Y + Oxidation (M)
22	655.3495	1308.6844	1308.5867	0.0977	0	100	2.3e-008	1	K.MDSTANEVEAVK.V + Oxidation (M)
23	484.6019	1450.7839	1450.6939	0.0899	0	34	0.07	1	K.YKPESEELTAER.I

N° in Table S1 N° spot protein identification

49 see on N°4
50 see on N°13

N° in Table S1 N° spot protein identification

51 1235 nucleophosmin

1. Q6V962_HUMAN Mass: 32754 Score: 251 Coverage: 19% number of peptides : 5
Nucleophosmin.- Homo sapiens (Human).
Check to include this hit in error tolerant search or archive report

Query	Observed	Mr(expt)	Mr(calc)	Delta	Miss	Score	Expect	Rank	Peptide
2	403.7180	805.4214	805.4004	0.0211	0	30	0.31	1	K.MQASIEK.G
7	466.2529	930.4912	930.4658	0.0254	0	52	0.0015	1	K.GPSSVEDIK.A
11	512.2606	1022.5066	1022.4821	0.0245	1	29	0.3	1	K.ADKDYHFK.V
15	565.8221	1129.6296	1129.5979	0.0318	1	84	8.3e-007	1	K.GPSSVEDIKAK.M
19	784.8965	1567.7784	1567.7226	0.0558	0	125	5.8e-011	1	K.VDNDENEHQLSLR.T

N° in Table S1 N° spot protein identification

52 1277 nucleophosmin

1. Q6V962_HUMAN Mass: 32754 Score: 53 Coverage: 3% number of peptides : 1
Nucleophosmin.- Homo sapiens (Human).
Check to include this hit in error tolerant search or archive report

Query	Observed	Mr(expt)	Mr(calc)	Delta	Miss	Score	Expect	Rank	Peptide
5	466.2403	930.4660	930.4658	0.0003	0	53	0,0015	1	K.GPSSVEDIK.A

N° in Table S1 N° spot protein identification

53 502 glycerol-3-phosphate dehydrogenase mitochondrial precursor

1. G02093 Mass: 81296 Score: 308 Coverage: 17%
glycerol-3-phosphate dehydrogenase (EC 1.1.99.5), mitochondrial precursor - human number of peptides : 13
Check to include this hit in error tolerant search or archive report

Query	Observed	Mr(expt)	Mr(calc)	Delta	Miss	Score	Expect	Rank	Peptide
5	417.2313	832.4480	832.4477	0.0004	0	35	0.081	1	R.IVELMGR.E + Oxidation (M)
9	468.7446	935.4746	935.4712	0.0034	0	30	0.23	1	K.LDIEQYR.M
10	474.2262	946.4378	946.4356	0.0023	0	48	0.0044	1	K.TAEENLDR.R
11	476.2918	950.5690	950.5661	0.0029	1	39	0.031	1	R.RVPIPVDR.S
12	487.7498	973.4850	973.4828	0.0022	0	31	0.22	1	K.QEQLETAR.K
13	491.8055	981.5964	981.5872	0.0092	1	24	0.8	1	K.RWPIVGVR.L
15	510.3004	1018.5862	1018.5811	0.0051	0	33	0.15	1	R.TVGLFLQGGK.D
20	580.8322	1159.6498	1159.6383	0.0116	0	53	0.0013	1	R.MNLAIALTAAR.Y + Oxidation (M)
22	605.7971	1209.5796	1209.5666	0.0131	1	26	0.59	1	R.ELNWDDYKK.Q
23	615.3274	1228.6402	1228.6274	0.0128	0	24	0.97	1	R.ALEHFPMLQK.D + Oxidation (M)
25	627.7998	1253.5850	1253.5710	0.0140	0	50	0.0022	1	R.NYLSCDVEVR.R
26	633.3107	1264.6068	1264.5969	0.0100	0	75	6.9e-006	1	R.SMAEDTINAAVK.T + Oxidation (M)
29	576.6112	1726.8118	1726.8122	-0.0004	1	110	1.5e-009	1	K.TALVERDDFSSGTSSR.S

N° in Table S1 N° spot protein identification

54 876 3-hydroxy-3-methylglutaryl-Coenzyme A synthase 1
Q5XJ04_HUMAN Mass: 57854 Score: 264 Coverage: 15% number of peptides : 8
3-hydroxy-3-methylglutaryl-Coenzyme A synthase 1 (Soluble).- Homo sapiens (Human).
Check to include this hit in error tolerant search or archive report

Query	Observed	Mr(expt)	Mr(calc)	Delta	Miss	Score	Expect	Rank	Peptide
19	475.7649	949.5152	949.5233	-0.0080	0	36	0.061	1	K.YTIGLGQAK.M
25	498.7495	995.4844	995.4924	-0.0079	0	49	0.0029	1	K.ASSELFSQK.T
31	510.7700	1019.5254	1019.5321	-0.0067	0	53	0.0013	1	K.ITASLCDLK.S
35	529.7398	1057.4650	1057.4716	-0.0066	0	37	0.073	1	K.LEDTYFDR.D
57	606.2737	1210.5328	1210.5401	-0.0072	0	33	0.098	1	R.NNLSYDCIGR.L
60	609.3322	1216.6498	1216.6551	-0.0052	0	89	3.2e-007	1	R.LEVGTETIIDK.S
64	651.8274	1301.6402	1301.6463	-0.0061	0	65	6.7e-005	1	K.VTQDATPGSALDK.I
70	697.8307	1393.6468	1393.6548	-0.0079	0	62	0.00012	1	R.TGVAPDVFAENMK.L + Oxidation (M)

N° in Table S1 N° spot protein identification

55 and 56 **1327** **Triosephosphate isomerase and ETHE1 protein**

1. TPIS_HUMAN Mass: 26807 Score: 495 Coverage: 36% number of peptides : 8

55 Triosephosphate isomerase (EC 5.3.1.1) (TIM) (Triose-phosphate isomerase).- Homo sapiens (Human).

Check to include this hit in error tolerant search or archive report

Query	Observed	Mr(expt)	Mr(calc)	Delta	Miss	Score	Expect	Rank	Peptide
6	425.7690	849.5234	849.4596	0.0638	0	25	0.95	1	K.VVFEQTK.V
14	569.3334	1136.6522	1136.5648	0.0874	0	64	0.0001	1	K.IAVAAQNCYK.V
17	617.8522	1233.6898	1233.5949	0.0949	0	81	1.7e-006	1	K.SNVSDAVAQSTR.I
19	630.8740	1259.7334	1259.6357	0.0977	1	45	0.0077	1	K.LDEREAGITEK.V
20	425.5896	1273.7470	1273.6666	0.0803	1	80	2.5e-006	1	K.VIADNVKDWSK.V
24	663.8861	1325.7576	1325.6649	0.0927	0	107	3.8e-009	1	R.IIYGGSVTGATCK.E
30	729.9150	1457.8154	1457.7151	0.1004	0	59	0.00026	1	R.HVFGESDELIGQK.V
31	489.6155	1465.8247	1465.7161	0.1086	0	72	9.7e-006	1	K.TATPQQAQEVHEK.L

2. O95571_HUMAN Mass: 28368 Score: 53 Coverage: 12% **number of peptides :** 2

56 HSCO protein (ETHE1 protein).- Homo sapiens (Human).

Check to include this hit in error tolerant search or archive report

Query	Observed	Mr(expt)	Mr(calc)	Delta	Miss	Score	Expect	Rank	Peptide
12	527.7763	1053.5380	1053.4549	0.0831	0	36	0,061	1	R.TDFQQGCAK.T
23	658.9011	1315.7876	1315.6918	0.0959	0	27	0,5	1	R.SLLPGCQSVISR.L

N° in Table S1 **N° spot** **protein identification**

57 **1056** **ubiquinol-cytochrome-c reductase**

AAH09586 Mass: 53297 Score:296 Coverage: 15% number of peptides : 8

BC009586 NID: - Homo sapiens ubiquinol-cytochrome-c reductase (EC 1.10.2.2) core protein I - human

Check to include this hit in error tolerant search or archive report

Query	Observed	Mr(expt)	Mr(calc)	Delta	Miss	Score	Expect	Rank	Peptide
2	405.2122	808.4098	808.4079	0.0019	0	30	0.24	1	R.FTGSEIR.H
11	456.7258	911.4370	911.4324	0.0047	0	55	0.00059	1	R.SGMFWLR.F + Oxidation (M)
16	512.7612	1023.5078	1023.5025	0.0053	0	34	0.079	1	R.EHTAYYIK.A
23	529.7980	1057.5814	1057.5768	0.0047	0	54	0.0014	1	R.IAEVDASVVR.E
26	550.7949	1099.5752	1099.5662	0.0091	0	48	0.0042	1	R.IPLAEWESR.I
42	605.7517	1209.4888	1209.4754	0.0135	0	42	0.016	1	R.EMQENDASMR.D
47	628.8456	1255.6766	1255.6673	0.0094	1	55	0.00075	1	R.RIPLAEWESR.I
52	662.3275	1322.6404	1322.6136	0.0268	0	92	1.3e-007	1	R.LCTSATESEVAR.G

N° in Table S1 **N° spot** **protein identification**

58 **266** **EF-2**

1. EF2_HUMAN Mass: 96115 Score:581 Coverage: 22% number of peptides : 16

Elongation factor 2 (EF-2).- Homo sapiens (Human).

Check to include this hit in error tolerant search or archive report

Query	Observed	Mr(expt)	Mr(calc)	Delta	Miss	Score	Expect	Rank	Peptide
5	427.2063	852.3980	852.4197	-0.0217	0	24	0.78	1	R.TILMMGR.Y + 2 Oxidation (M)
8	456.2040	910.3934	910.4185	-0.0250	0	30	0.19	1	R.YFDPANGK.F
14	507.2425	1012.4704	1012.4938	-0.0233	0	38	0.036	1	K.GEGQLGPAER.A
20	536.2560	1070.4974	1070.5140	-0.0166	0	40	0.023	1	K.GPLMMYISK.M + 2 Oxidation (M)
23	546.2878	1090.5610	1090.5771	-0.0161	0	43	0.013	1	-.VNFTVDQIR.A
24	547.2584	1092.5022	1092.5274	-0.0251	0	33	0.11	1	R.IMGPNYTPGK.K + Oxidation (M)
26	551.7529	1101.4912	1101.5165	-0.0252	0	41	0.018	1	K.QFAEMYVAK.F + Oxidation (M)
27	554.3174	1106.6202	1106.6336	-0.0133	0	78	4.2e-006	1	R.VFSGLVSTGLK.V
31	562.2753	1122.5360	1122.5591	-0.0230	0	65	6.6e-005	1	K.STLTDSLVCK.A
52	604.7434	1207.4722	1207.4961	-0.0239	0	49	0.0025	1	K.EGALCEENMR.G
65	451.2427	1350.7063	1350.7507	-0.0444	1	66	5.2e-005	1	R.VAVEAKNPADLPK.L
68	689.8553	1377.6960	1377.7075	-0.0114	0	71	1.9e-005	1	R.CLYASVLTAQPR.L
73	468.2597	1401.7573	1401.7980	-0.0407	1	63	0.00011	1	K.KEDLYLKPIQR.T
78	477.5694	1429.6864	1429.7314	-0.0450	1	58	0.00032	1	K.FAAKGEGQLGPAER.A
86	797.8822	1593.7498	1593.7556	-0.0057	0	64	7.1e-005	1	R.ETVSEESNVLCLSK.S
87	544.5774	1630.7104	1630.7522	-0.0418	0	63	0.0001	1	K.TGTITTFEHAHNMR.V + Oxidation (M)

N° in Table S1 **N° spot** **protein identification**

59 **116** **Leucine-rich PPR motif-containing protein**

AAA67549 Mass: 146306 Score: 136 Coverage: 4% number of peptides : 5

HUM130LEU NID: - Homo sapiens

Check to include this hit in error tolerant search or archive report

Query	Observed	Mr(expt)	Mr(calc)	Delta	Miss	Score	Expect	Rank	Peptide
15	549.3336	1096.6526	1096.5876	0.0650	0	33	0.096	1	K.IQEENVIPR.E
23	636.8671	1271.7196	1271.6470	0.0727	0	60	0.0002	1	K.TVLDQQQTPSR.L

Query	Observed	Mr(expt)	Mr(calc)	Delta	Miss	Score	Expect	Rank	Peptide
24	657.3735	1312.7324	1312.6510	0.0814	1	60	0.00023	1	K.SYVSEKDVTSAK.A
28	458.2872	1371.8398	1371.7609	0.0788	1	35	0.071	1	K.LVEKGETDLIQK.A
31	702.8856	1403.7566	1403.6715	0.0852	0	69	2.8e-005	1	R.CVANNQVETLEK.L

N° in Table S1 N° spot protein identification

60 859 C-terminal binding protein 2

Q5SQP7_HUMAN Mass: 106748 Score: 193 Coverage: 4% number of peptides : 5
C-terminal binding protein 2.- Homo sapiens (Human).
Check to include this hit in error tolerant search or archive report

Query	Observed	Mr(expt)	Mr(calc)	Delta	Miss	Score	Expect	Rank	Peptide
6	479.7773	957.5400	957.5243	0.0157	0	50	0.0025	1	R.VQSVEQIR.E
8	508.7689	1015.5232	1015.5410	-0.0178	1	42	0.016	1	R.EAAATEIRR.A
10	523.7810	1045.5474	1045.5669	-0.0194	0	62	0.00017	1	R.QGAFLVNAAR.G
13	590.7838	1179.5530	1179.5772	-0.0241	0	93	1.1e-007	1	R.IGSGYDNVDIK.A
16	611.2844	1220.5542	1220.5781	-0.0238	0	28	0.4	1	R.DCTVEMPILK.D + Oxidation (M)

N° in Table S1 N° spot protein identification

61 303 N-ras upstream protein NRU

1. Q9GZV0_HUMAN Mass: 86418 Score:270 Coverage: 19% number of peptides : 14
Hypothetical protein FLJ12466 (Hypothetical protein FLJ12454).- Homo sapiens (Human). N-ras upstream protein NRU - human
Check to include this hit in error tolerant search or archive report

Query	Observed	Mr(expt)	Mr(calc)	Delta	Miss	Score	Expect	Rank	Peptide
10	476.7649	951.5152	951.5250	-0.0097	0	30	0.24	1	K.AVAAPRPDR.L
16	526.7169	1051.4192	1051.4328	-0.0135	0	49	0.0029	1	K.CSACNVWR.V
18	526.7327	1051.4508	1051.4613	-0.0105	0	25	0.67	1	R.CQGVVCAMK.E
22	569.8106	1137.6066	1137.6142	-0.0076	0	42	0.014	1	K.VTLLEGDHVR.F
23	575.2873	1148.5600	1148.5713	-0.0113	1	27	0.43	1	K.ELPFGDKDTK.S
24	586.7928	1171.5710	1171.5833	-0.0123	0	41	0.016	1	K.NITLDDASAPR.L
28	627.2589	1252.5032	1252.5143	-0.0110	0	54	0.00073	1	R.GPDNSMGFGAER.K + Oxidation (M)
30	627.8499	1253.6852	1253.6979	-0.0127	1	41	0.017	1	K.IKQEILPEER.M
31	451.9132	1352.7178	1352.7412	-0.0234	1	61	0.00014	1	K.SKVTLLEGDHVR.F
33	456.2045	1365.5917	1365.6174	-0.0258	0	53	0.0011	1	K.GTVSFHSHSDHR.F
34	461.2013	1380.5821	1380.6092	-0.0271	1	27	0.41	1	R.GPDNSMGFGAERK.I + Oxidation (M)
37	467.2358	1398.6856	1398.7150	-0.0295	0	46	0.0051	1	K.QRPGQQVATCVR.L
39	474.5777	1420.7113	1420.7423	-0.0310	1	34	0.082	1	K.VPSKNQNDPLPGR.I
41	537.2435	1608.7087	1608.7380	-0.0293	1	49	0.0021	1	K.VGDDVEFEVSSDRR.T

N° in Table S1 N° spot protein identification

62 300 N-ras upstream protein NRU

1. Q9GZV0_HUMAN Mass: 86418 Score:140 Coverage: 10% number of peptides : 7
Hypothetical protein FLJ12466 (Hypothetical protein FLJ12454).- Homo sapiens (Human). N-ras upstream protein NRU - human
Check to include this hit in error tolerant search or archive report

Query	Observed	Mr(expt)	Mr(calc)	Delta	Miss	Score	Expect	Rank	Peptide
10	505.8006	1009.5866	1009.4941	0.0926	0	35	0.058	1	K.NQNDPLPGR.I
13	526.7648	1051.5150	1051.4328	0.0823	0	48	0.0034	1	K.CSACNVWR.V
14	526.7849	1051.5552	1051.4613	0.0939	0	29	0.23	1	R.CQGVVCAMK.E
17	569.8644	1137.7142	1137.6142	0.1000	0	42	0.012	1	K.VTLLEGDHVR.F
18	575.3450	1148.6754	1148.5713	0.1041	1	26	0.64	1	K.ELPFGDKDTK.S
19	586.8484	1171.6822	1171.5833	0.0989	0	34	0.083	1	K.NITLDDASAPR.L
24	627.3198	1252.6250	1252.5143	0.1108	0	66	5.5e-005	1	R.GPDNSMGFGAER.K + Oxidation (M)

N° in Table S1 N° spot protein identification

63 304 N-ras upstream protein NRU

1. Q9GZV0_HUMAN Mass: 86418 Score: 375 Coverage: 13% number of peptides : 12
Hypothetical protein FLJ12466 (Hypothetical protein FLJ12454).- Homo sapiens (Human). N-ras upstream protein NRU - human
Check to include this hit in error tolerant search or archive report

Query	Observed	Mr(expt)	Mr(calc)	Delta	Miss	Score	Expect	Rank	Peptide
9	476.8052	951.5958	951.5250	0.0709	0	27	0.39	1	K.AVAAPRPDR.L
10	489.2808	976.5470	976.4834	0.0637	0	37	0.062	1	R.EMGVIAAMR.D
13	505.7878	1009.5610	1009.4941	0.0670	0	34	0.078	1	K.NQNDPLPGR.I
16	526.7562	1051.4978	1051.4328	0.0651	0	48	0.0034	1	K.CSACNVWR.V
17	526.7716	1051.5286	1051.4613	0.0673	0	28	0.34	1	R.CQGVVCAMK.E
19	569.8522	1137.6898	1137.6142	0.0756	0	58	0.00035	1	K.VTLLEGDHVR.F

Query	Observed	Mr(expt)	Mr(calc)	Delta	Miss	Score	Expect	Rank	Peptide
20	575.3294	1148.6442	1148.5713	0.0729	1	47	0.0044	1	K.ELPFGDKDTK.S
22	586.8357	1171.6568	1171.5833	0.0736	0	32	0.15	1	K.NITLDDASAPR.L
26	627.3074	1252.6002	1252.5143	0.0860	0	86	5.6e-007	1	R.GPDNSMGFGAER.K + Oxidation (M)
29	456.2413	1365.7021	1365.6174	0.0846	0	72	1.2e-005	1	K.GTVSFHSHSDHR.F
30	461.2357	1380.6853	1380.6092	0.0761	1	45	0.006	1	R.GPDNSMGFGAERK.I + Oxidation (M)
34	474.6168	1420.8286	1420.7423	0.0863	1	44	0.0076	1	K.VPSKNQNDPLPGR.I

N° in Table S1	N° spot	protein identification
**64**	**746**	**Heterogeneous nuclear ribonucleoprotein K.**

1. Q5T6W5_HUMAN Mass: 47756 Score: 459 Coverage: 22 % number of peptides : 10
Heterogeneous nuclear ribonucleoprotein K.- Homo sapiens (Human).
Check to include this hit in error tolerant search or archive report

Query	Observed	Mr(expt)	Mr(calc)	Delta	Miss	Score	Expect	Rank	Peptide
9	507.2446	1012.4746	1012.4284	0.0463	0	55	0.00068	1	R.GGDLMAYDR.R + Oxidation (M)
12	507.7201	1013.4256	1013.3760	0.0496	0	34	0.095	1	R.DYDDMSPR.R + Oxidation (M)
14	527.3482	1052.6818	1052.6342	0.0476	0	38	0.039	1	R.VVLIGGKPDR.V
20	549.7581	1097.5016	1097.4448	0.0569	0	48	0.0032	1	K.GSDFDCELR.L
21	553.7883	1105.5620	1105.5074	0.0547	0	53	0.0011	1	R.NTDEMVELR.I
32	597.8840	1193.7534	1193.6921	0.0614	0	51	0.0017	1	R.NLPLPPPPPR.G
35	630.3201	1258.6256	1258.5677	0.0579	0	84	7.4e-007	1	K.IDEPLEGSEDR.I
44	455.9030	1364.6872	1364.6354	0.0518	1	36	0.05	1	R.SRNTDEMVELR.I + Oxidation (M)
46	517.2415	1548.7027	1548.6450	0.0577	0	61	0.00017	1	K.LFQECCPHSTDR.V
55	584.6285	1750.8637	1750.7944	0.0693	1	71	1.4e-005	1	K.RPAEDMEEEQAFKR.S + Oxidation (M)

N° in Table S1	N° spot	protein identification
**65**	**283**	**striatin 3**

3. STRN3_HUMAN Mass: 87478 Score: 89 Coverage: 3% number of peptides : 2
Striatin 3 (Cell-cycle autoantigen SG2NA) (S/G2 antigen).- Homo sapiens (Human).
Check to include this hit in error tolerant search or archive report

Query	Observed	Mr(expt)	Mr(calc)	Delta	Miss	Score	Expect	Rank	Peptide
15	611.3366	1220.6586	1220.6401	0.0186	0	49	0.0031	1	K.AYIASAGADALAK.V
23	710.8593	1419.7040	1419.6776	0.0264	0	63	8.9e-005	1	K.NQLLSCSADGTVR.L

N° in Table S1	N° spot	protein identification
**66**	**1240**	**hepatoma-derived growth factor**

1. A55055 Mass: 26886 Score: 217 Coverage: 16% number of peptides : 4
hepatoma-derived growth factor - human
Check to include this hit in error tolerant search or archive report

Query	Observed	Mr(expt)	Mr(calc)	Delta	Miss	Score	Expect	Rank	Peptide
5	455.2465	908.4784	908.4426	0.0359	0	58	0.00032	1	K.CGDLVFAK.M
7	522.7817	1043.5488	1043.5135	0.0354	0	87	5.4e-007	1	R.AGDLLEDSPK.R
9	544.7685	1087.5224	1087.4894	0.0330	0	30	0.27	1	K.NSTPSEPGSGR.G
11	559.7924	1117.5702	1117.5325	0.0377	0	42	0.015	1	R.IDEMPEAAVK.S + Oxidation (M)

N° in Table S1	N° spot	protein identification
**67**	**805**	**EGF-containing fibulin-like extracellular matrix protein 1**

1. AAH14410 Mass: 56885 Score: 254 Coverage: 15% number of peptides : 6
BC014410 NID: - Homo sapiens
Check to include this hit in error tolerant search or archive report

Query	Observed	Mr(expt)	Mr(calc)	Delta	Miss	Score	Expect	Rank	Peptide
9	538.8165	1075.6184	1075.5913	0.0271	0	34	0.088	1	R.ELPQSIVYK.Y
10	544.7917	1087.5688	1087.5444	0.0244	0	48	0.0042	1	R.ADQVCINLR.G
14	643.3100	1284.6054	1284.5735	0.0320	0	81	1.7e-006	1	K.SGNENGEFYLR.Q
15	662.2857	1322.5568	1322.5231	0.0338	0	78	3.5e-006	1	R.LNCEDIDECR.T
16	677.2996	1352.5846	1352.5458	0.0389	0	52	0.0013	1	R.CVCPVSNAMCR.E
22	562.6321	1684.8745	1684.8393	0.0351	1	83	8.5e-007	1	R.NPADPQRIPSNPSHR.I

N° in Table S1	N° spot	protein identification
**68**		see on N°8
**69**		see on N°39

Table S2B. LC-MS MS data on proteins for which only one peptide was identified.

N° in Table S1	N° spot	protein identification
7	740	**dihydropyrimidinase-related protein 2**

JC5317 Mass: 62711 Score: 41 Coverage: 1% number of peptides : 1
dihydropyrimidinase-related protein 2 - human
Check to include this hit in error tolerant search or archive report

Query	Observed	Mr(expt)	Mr(calc)	Delta	Miss	Score	Expect	Rank	Peptide
3	516.3078	1030.6010	1030.5407	0.0604	0	41	0,019	1	K.SSAEVIAQAR.K

N° in Table S1	N° spot	protein identification
29	418	**Coronin-7**

BAB15211 Mass: 101596 Score: 59 Queries matched: 1 number of peptides : 1
AK025674 NID: - Homo sapiens (Coronin-7)
Check to include this hit in error tolerant search or archive report

Query	Observed	Mr(expt)	Mr(calc)	Delta	Miss	Score	Expect	Rank	Peptide
5	646.8463	1291.6780	1291.6408	0.0373	0	59	0.00049	1	R.SGPEPLQEGPGPK.G

N° in Table S1	N° spot	protein identification
34	1480	**glutathione S transferase P**
	1.	

GTP_HUMAN Mass: 23438 Score: 55 Coverage: 4% number of peptides : 1
Glutathione S-transferase P (EC 2.5.1.18) (GST class-pi) (GSTP1-1).- Homo sapiens (Human).
Check to include this hit in error tolerant search or archive report

Query	Observed	Mr(expt)	Mr(calc)	Delta	Miss	Score	Expect	Rank	Peptide
4	568.8204	1135.6262	1135.5696	0.0567	0	55	0.00076	1	K.ASCLYGQLPK.F

N° in Table S1	N° spot	protein identification
52	1277	**nucleophosmin**
	1.	

Q6V962_HUMAN Mass: 32754 Score: 53 Coverage: 3% number of peptides : 1
Nucleophosmin.- Homo sapiens (Human).
Check to include this hit in error tolerant search or archive report

Query	Observed	Mr(expt)	Mr(calc)	Delta	Miss	Score	Expect	Rank	Peptide
5	466.2403	930.4660	930.4658	0.0003	0	53	0,0015	1	K.GPSSVEDIK.A

N° in Table S1	N° spot	protein identification
18	986	

K1CP_HUMAN Mass: 51447 Score: 223 Coverage: 17% number of peptides : 10
Keratin, type I cytoskeletal 16 (Cytokeratin 16) (K16) (CK 16).- Homo sapiens (Human).
Check to include this hit in error tolerant search or archive report

Query	Observed	Mr(expt)	Mr(calc)	Delta	Miss	Score	Expect	Rank	Peptide
1	404.2005	806.3864	806.3923	-0.0058	0	40	0.029	1	R.LAADDFR.T
2	405.2203	808.4260	808.4330	-0.0070	0	44	0.01	1	R.LASYLDK.V
14	515.2975	1028.5804	1028.5866	-0.0061	0	46	0.0067	1	R.VLDELTLAR.T
16	518.7676	1035.5206	1035.5250	-0.0043	1	32	0.14	1	K.IRDWYQR.Q
22	532.8054	1063.5962	1063.6026	-0.0063	1	28	0.37	1	R.LASYLDKVR.A
25	553.7610	1105.5074	1105.5186	-0.0112	0	86	6.5e-007	1	K.VTMQNLNDR.L + Oxidation (M)
26	553.7827	1105.5508	1105.5550	-0.0041	0	45	0.008	1	R.ISSVLAGGSCR.A
30	561.7925	1121.5704	1121.5717	-0.0012	0	61	0.00022	1	R.LEQEIATYR.R
33	610.8013	1219.5880	1219.5932	-0.0051	0	69	3.2e-005	1	K.ASLENSLEETK.G
39	630.7877	1259.5608	1259.5630	-0.0021	0	46	0.0057	1	R.EVFTSSSSSSSR.Q

N° in Table S1	N° spot	protein identification
30	1302	**Hsp-27 Phospho S82**
	1.	

HHHU27 Mass: 22826 Score:375 Coverage: 24% number of peptides : 6
heat shock protein 27 - human
Check to include this hit in error tolerant search or archive report

Query	Observed	Mr(expt)	Mr(calc)	Delta	Miss	Score	Expect	Rank	Peptide
9	471.3003	940.5860	940.4978	0.0883	0	33	0.23	1	R.AQLGGPEAAK.S
20	552.8088	1103.6030	1103.4996	0.1035	0	61	0.00034	1	R.QDEHGYISR.C
22	573.8721	1145.7296	1145.6292	0.1004	1	65	0.00013	1	K.TKDGVVEITGK.H
23	578.3216	1154.6286	1154.5332	0.0954	0	71	4e-005	1	R.QLSSGVSEIR.H + Phospho (ST)
33	490.2900	1467.8482	1467.7318	0.1164	1	70	4.4e-005	1	K.DGVVEITGKHEER.Q
34	548.6583	1642.9531	1642.8162	0.1369	1	116	9.7e-010	1	R.AQLGGPEAAKSDETAAK.-

S3 : Phospho (ST), with neutral losses 97.9769(shown in table), 0.0000
Ions Score: 71 **Expect:** 4e-005

Table S2C spot 740

{MATRIX SCIENCE} Mascot Search Results

Peptide View

MS/MS Fragmentation of **SSAEVIAQAR**
Found in **JC5317**, dihydropyrimidinase-related protein 2 - human

Match to Query 3: 1030.601048 from(516.307800,2+) intensity(759.7000)
Data file C:\Documents and Settings\micromass\Desktop\pkl\vv_740_050915_39.pkl

Click mouse within plot area to zoom in by factor of two about that point
Or, Plot from 50 to 1050 Da Full range

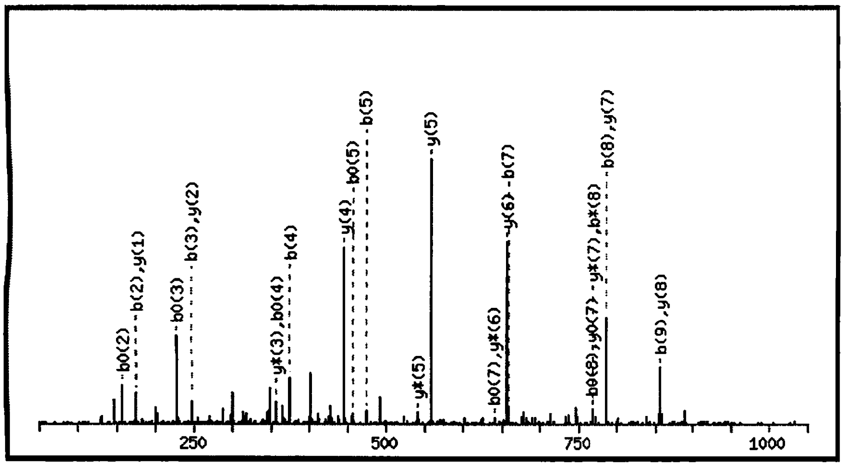

Monoisotopic mass of neutral peptide Mr(calc): 1030.5407
Fixed modifications: Carbamidomethyl (C)
Ions Score: 41 **Expect:** 0.035
Matches (Bold Red): 26/82 fragment ions using 47 most intense peaks

#	b	b^{++}	b*	b^{*++}	b^0	b^{0++}	Seq.	y	y^{++}	y*	y^{*++}	y^0	y^{0++}	#
1	88.0393	44.5233			70.0287	35.5180	S							1
2	**175.0713**	88.0393			**157.0608**	79.0340	S	944.5160	472.7616	927.4894	464.2483	926.5054	463.7563	
3	**246.1084**	123.5579			**228.0979**	114.5526	A	**857.4839**	429.2456	840.4574	420.7323	839.4734	420.2403	
4	**375.1510**	188.0792			**357.1405**	179.0739	E	**786.4468**	393.7270	**769.4203**	385.2138	**768.4362**	384.7218	
5	**474.2194**	237.6134			**456.2089**	228.6081	V	**657.4042**	329.2057	**640.3777**	320.6925			
6	587.3035	294.1554			569.2929	285.1501	I	**558.3358**	279.6715	**541.3093**	271.1583			
7	**658.3406**	329.6739			**640.3301**	320.6687	A	**445.2518**	223.1295	428.2252	214.6162			
8	**786.3992**	393.7032	**769.3726**	385.1900	**768.3886**	384.6980	Q	374.2146	187.6110	**357.1881**	179.0977			
9	**857.4363**	429.2218	840.4098	420.7085	839.4257	420.2165	A	**246.1561**	123.5817	229.1295	115.0684			
10							R	**175.1190**	88.0631	158.0924	79.5498			

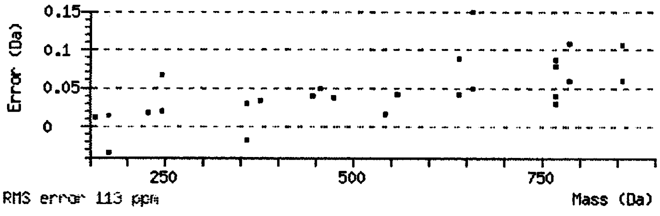

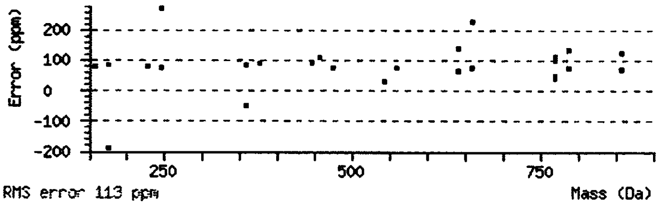

NCBI **BLAST** search of SSAEVIAQAR
(Parameters: blastp, nr protein database, expect=20000, no filter, PAM30)

Table S2C spot 740

Other BLAST web gateways

All matches to this query

Score	Mr(calc):	Delta	Sequence
41.0	1030.5407	0.0604	SSAEVIAQAR
19.0	1030.5957	0.0054	MARVIIXNK
18.7	1030.4865	0.1145	AGVCGDPSLR
17.2	1030.5195	0.0815	GIXWKDEAR
16.2	1030.4341	0.1670	MQMAVSLR
13.7	1030.5519	0.0491	TSEGVLRNR
13.5	1030.5229	0.0781	CDVVGLAAAR
11.7	1030.5883	0.0127	RNSSLLSVR
11.3	1030.4518	0.1492	MSAATVLSR
11.2	1030.4679	0.1331	SEASPENAAR

Mascot: http://www.matrixscience.com/

{MATRIX SCIENCE} Mascot Search Results

Peptide View

MS/MS Fragmentation of **SGPEPLQEGPGPK**
Found in **Q17RK4_HUMAN**, Coronin 7.- Homo sapiens (Human).

Match to Query 5: 1291.678048 from(646.846300,2+) intensity(797.4000)
Data file C:\Documents and Settings\micromass\Desktop\pkl\otherPKl\vv_418_050915_33.pkl

Click mouse within plot area to zoom in by factor of two about that point
Or, Plot from 0 to 1300 Da Full range

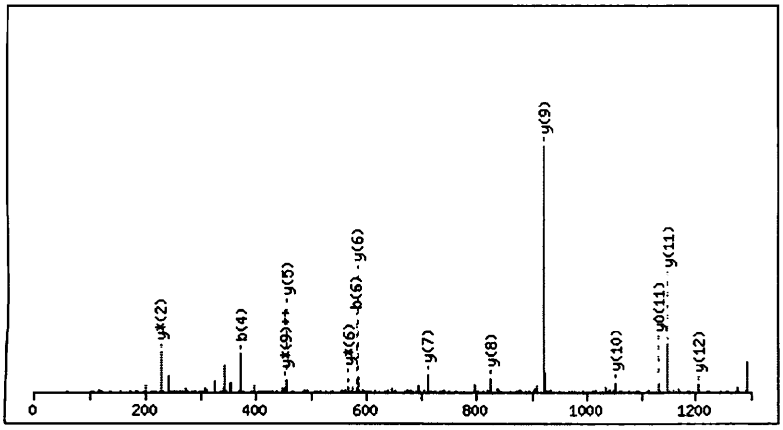

Monoisotopic mass of neutral peptide Mr(calc): 1291.6408
Fixed modifications: Carbamidomethyl (C)
Ions Score: 56 **Expect:** 0.00057
Matches (Bold Red): 14/122 fragment ions using 24 most intense peaks

#	b	b^{++}	b*	b^{*++}	b^0	b^{0++}	Seq.	y	y^{++}	y*	y^{*++}	y^0	y^{0++}	#
1	88.0393	44.5233			70.0287	35.5180	S							13
2	145.0608	73.0340			127.0502	64.0287	G	**1205.6161**	603.3117	1188.5895	594.7984	1187.6055	594.3064	12
3	242.1135	121.5604			224.1030	112.5551	P	**1148.5946**	574.8009	1131.5681	566.2877	**1130.5841**	565.7957	11
4	**371.1561**	186.0817			353.1456	177.0764	E	**1051.5419**	526.2746	1034.5153	517.7613	1033.5313	517.2693	10
5	468.2089	234.6081			450.1983	225.6028	P	**922.4993**	461.7533	905.4727	**453.2400**	904.4887	452.7480	9
6	**581.2930**	291.1501			563.2824	282.1448	L	**825.4465**	413.2269	808.4199	404.7136	807.4359	404.2216	8
7	709.3515	355.1794	692.3250	346.6661	691.3410	346.1741	Q	**712.3624**	356.6849	695.3359	348.1716	694.3519	347.6796	7
8	838.3941	419.7007	821.3676	411.1874	820.3836	410.6954	E	**584.3039**	292.6556	**567.2773**	284.1423	566.2933	283.6503	6
9	895.4156	448.2114	878.3890	439.6982	877.4050	439.2061	G	**455.2613**	228.1343	438.2347	219.6210			5
10	992.4684	496.7378	975.4418	488.2245	974.4578	487.7325	P	398.2398	199.6235	381.2132	191.1103			4
11	1049.4898	525.2485	1032.4633	516.7353	1031.4793	516.2433	G	301.1870	151.0972	284.1605	142.5839			3
12	1146.5426	573.7749	1129.5160	565.2617	1128.5320	564.7696	P	244.1656	122.5864	**227.1390**	114.0731			2
13							K	147.1128	74.0600	130.0863	65.5468			1

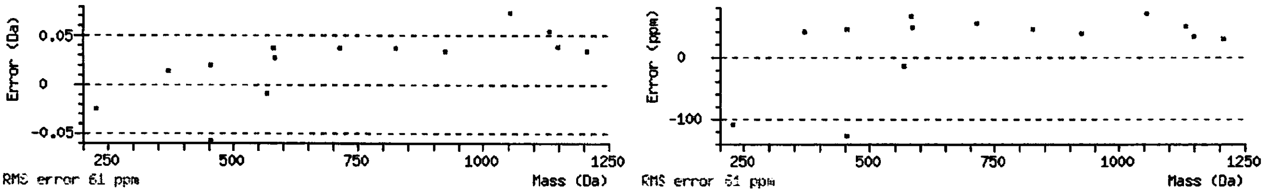

NCBI **BLAST** search of SGPEPLQEGPGPK
(Parameters: blastp, nr protein database, expect=20000, no filter, PAM30)
Other BLAST web gateways

All matches to this query

Score	Mr(calc):	Delta	Sequence
55.8	1291.6408	0.0372	SGPEPLQEGPGPK
16.4	1291.6997	-0.0216	SGLIPPREGPGGR

8.1	1291.7473	-0.0692	SRPSRIPQPVR
2.0	1291.6521	0.0260	GSLLSNLGFNDR
1.9	1291.6884	-0.0104	QLEQTNYRLK
1.9	1291.6521	0.0260	LISGIDNYGQGR
1.8	1291.7904	-0.1123	ILVAALTFFLGK
1.5	1291.7976	-0.1195	LTRPPKVLGLQA
1.4	1291.6772	0.0009	LQEIYQELTR
1.1	1291.5788	0.0993	AVAEEMMPAAEK

Mascot: http://www.matrixscience.com/

{MATRIX SCIENCE} Mascot Search Results

Peptide View

MS/MS Fragmentation of **ASCLYGQLPK**
Found in **GSTP1_HUMAN**, Glutathione S-transferase P (EC 2.5.1.18) (GST class-pi) (GSTP1-1).- Homo sapiens (Human).

Match to Query 4: 1135.626248 from(568.820400,2+) intensity(880.2000)
Data file C:\Documents and Settings\micromass\Desktop\pkl\otherPKl\vv_1480_050915_32.pkl

Click mouse within plot area to zoom in by factor of two about that point
Or, [Plot from] 0 to 1200 Da [Full range]

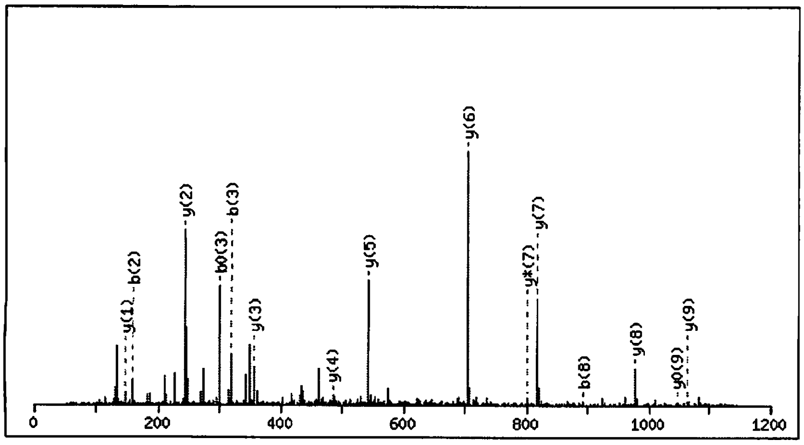

Monoisotopic mass of neutral peptide Mr(calc): 1135.5696
Fixed modifications: Carbamidomethyl (C)
Ions Score: 55 **Expect:** 0.00076
Matches (Bold Red): 15/78 fragment ions using 40 most intense peaks

#	b	b^{++}	b*	b^{*++}	b^0	b^{0++}	Seq.	y	y^{++}	y*	y^{*++}	y^0	y^{0++}	#
1	72.0444	36.5258					A							10
2	**159.0764**	80.0418			141.0659	71.0366	S	**1065.5397**	533.2735	1048.5132	524.7602	**1047.5292**	524.2682	9
3	**319.1071**	160.0572			**301.0965**	151.0519	C	**978.5077**	489.7575	961.4812	481.2442			8
4	432.1911	216.5992			414.1806	207.5939	L	**818.4771**	409.7422	**801.4505**	401.2289			7
5	595.2545	298.1309			577.2439	289.1256	Y	**705.3930**	353.2001	688.3665	344.6869			6
6	652.2759	326.6416			634.2654	317.6363	G	**542.3297**	271.6685	525.3031	263.1552			5
7	780.3345	390.6709	763.3080	382.1576	762.3239	381.6656	Q	**485.3082**	243.1577	468.2817	234.6445			4
8	**893.4186**	447.2129	876.3920	438.6996	875.4080	438.2076	L	**357.2496**	179.1285	340.2231	170.6152			3
9	990.4713	495.7393	973.4448	487.2260	972.4608	486.7340	P	**244.1656**	122.5864	227.1390	114.0731			2
10							K	**147.1128**	74.0600	130.0863	65.5468			1

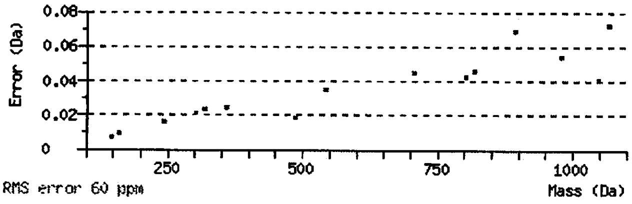

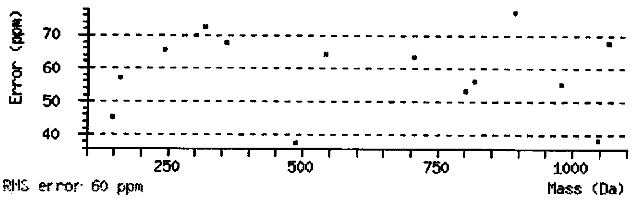

NCBI **BLAST** search of ASCLYGQLPK
(Parameters: blastp, nr protein database, expect=20000, no filter, PAM30)
Other BLAST web gateways

All matches to this query

Score	Mr(calc):	Delta	Sequence
54.8	1135.5696	0.0567	ASCLYGQLPK
17.4	1135.5179	0.1083	XSCEATGVDVK
3.3	1135.5146	0.1117	NEFGEIQGDK
2.3	1135.5179	0.1083	AAEGVSAADMAK
0.2	1135.5986	0.0277	AGNPGTPGAPGLK
0.2	1135.5550	0.0713	FLEDSLLNW

Mascot: http://www.matrixscience.com/

Table S2C spot 1277

Mascot Search Results

Peptide View

MS/MS Fragmentation of **GPSSVEDIK**
Found in **Q6V962_HUMAN**, Nucleophosmin.- Homo sapiens (Human).

Match to Query 5: 930.466048 from(466.240300,2+) intensity(715.9000)
Data file C:\Documents and Settings\micromass\Desktop\pkl\vv_1277_050915_25.pkl

Click mouse within plot area to zoom in by factor of two about that point
Or, Plot from 0 to 950 Da Full range

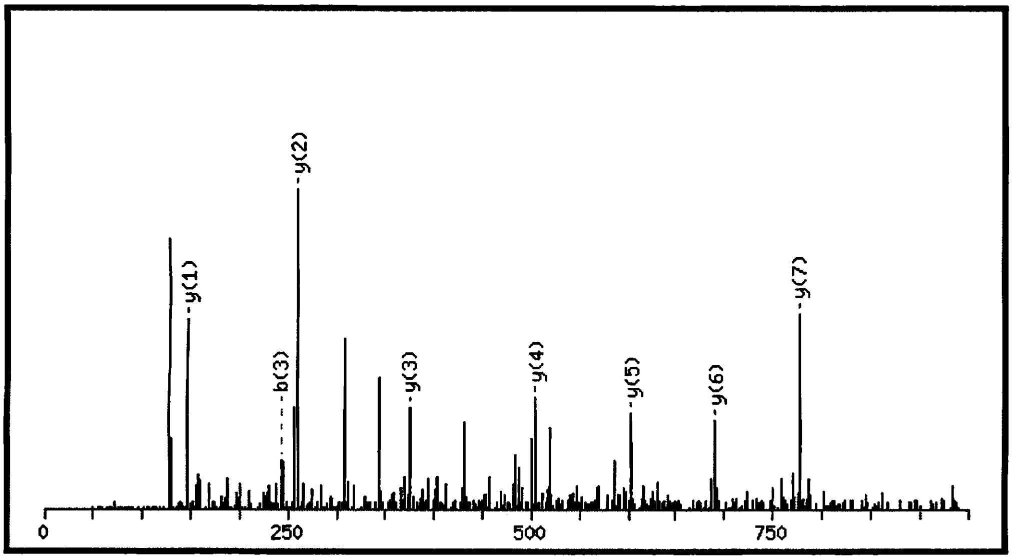

Monoisotopic mass of neutral peptide Mr(calc): 930.4658
Fixed modifications: Carbamidomethyl (C)
Ions Score: 53 **Expect:** 0.0024
Matches (Bold Red): 8/72 fragment ions using 18 most intense peaks

#	b	b++	b0	b0++	Seq.	y	y++	y*	y*++	y0	y0++	#
1	58.0287	29.5180			G							9
2	155.0815	78.0444			P	874.4516	437.7294	857.4251	429.2162	856.4411	428.7242	8
3	**242.1135**	121.5604	224.1030	112.5551	S	**777.3989**	389.2031	760.3723	380.6898	759.3883	380.1978	7
4	329.1456	165.0764	311.1350	156.0711	S	**690.3668**	345.6871	673.3403	337.1738	672.3563	336.6818	6
5	428.2140	214.6106	410.2034	205.6053	V	**603.3348**	302.1710	586.3083	293.6578	585.3242	293.1658	5
6	557.2566	279.1319	539.2460	270.1266	E	**504.2664**	252.6368	487.2398	244.1236	486.2558	243.6316	4
7	672.2835	336.6454	654.2729	327.6401	D	**375.2238**	188.1155	358.1973	179.6023	357.2132	179.1103	3
8	785.3676	393.1874	767.3570	384.1821	I	**260.1969**	130.6021	243.1703	122.0888			2
9					K	**147.1128**	74.0600	130.0863	65.5468			1

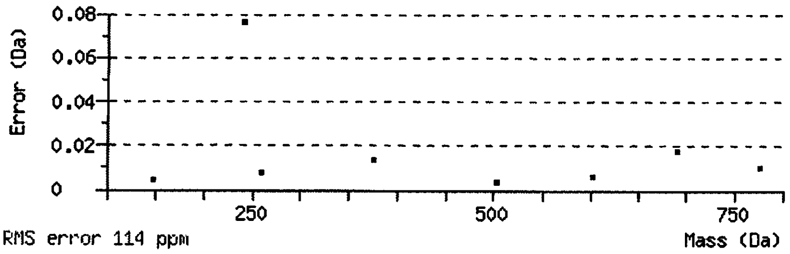

Table S2C spot 1277

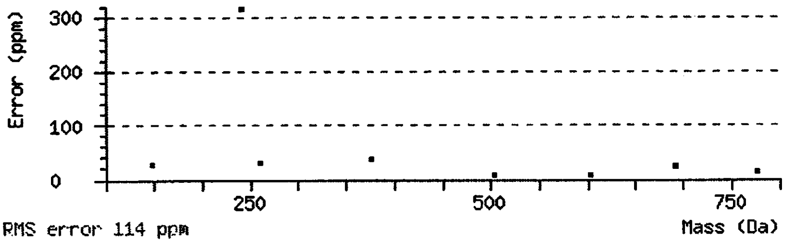

NCBI **BLAST** search of GPSSVEDIK
(Parameters: blastp, nr protein database, expect=20000, no filter, PAM30)
Other BLAST web gateways

All matches to this query

Score	Mr(calc):	Delta	Sequence
52.7	930.4658	0.0003	GPSSVEDIK
13.1	930.4997	-0.0336	GSPWMLKL
9.4	930.4099	0.0561	AYIEDLK
9.4	930.4099	0.0561	FSIEDLK
9.4	930.4099	0.0561	YAIEDLK
9.2	930.5022	-0.0361	XTVLEDLK
8.8	930.3517	0.1143	GSDGGSMLK
8.5	930.4286	0.0375	FTVPMLK
8.3	930.5022	-0.0361	LTVXEDLK
6.7	930.4000	0.0660	YGLXHFK

Mascot: http://www.matrixscience.com/

Table S2C spot 986_ K16

{MATRIX SCIENCE} Mascot Search Results

Peptide View

MS/MS Fragmentation of **EVFTSSSSSSSR**
Found in **K1C16_HUMAN**, Keratin, type I cytoskeletal 16 (Cytokeratin-16) (CK-16) (Keratin-16) (K16).- Homo sapiens (Human).

Match to Query 39: 1259.560848 from(630.787700,2+) intensity(1460.7000)
Data file C:\Documents and Settings\micromass\Desktop\pkl\otherPKI\vv_986_050915_44.pkl

Click mouse within plot area to zoom in by factor of two about that point
Or, Plot from 0 to 1300 Da Full range

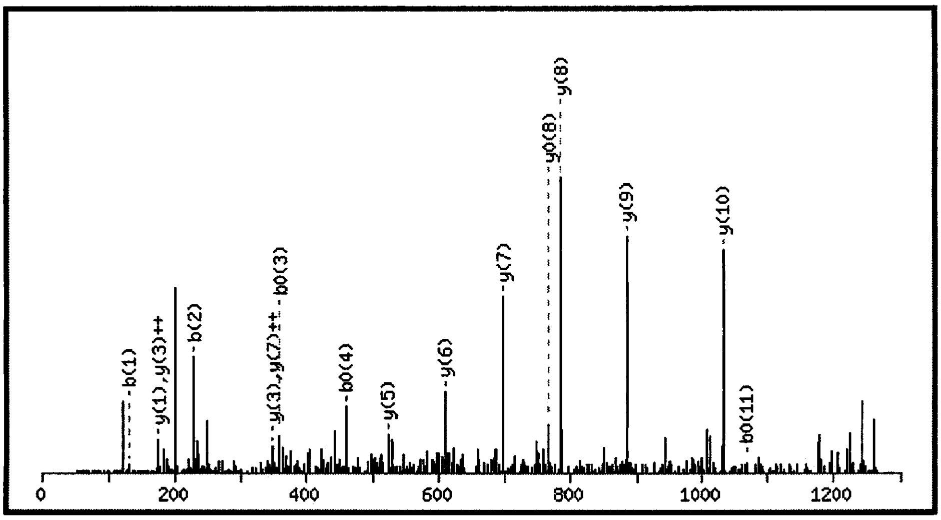

Monoisotopic mass of neutral peptide Mr(calc): 1259.5630
Fixed modifications: Carbamidomethyl (C)
Ions Score: 46 **Expect:** 0.0057
Matches (Bold Red): 16/108 fragment ions using 45 most intense peaks

#	b	b^{++}	b^0	b^{0++}	Seq.	y	y^{++}	y*	y^{*++}	y^0	y^{0++}	#
1	**130.0499**	65.5286	112.0393	56.5233	E							12
2	**229.1183**	115.0628	211.1077	106.0575	V	1131.5277	566.2675	1114.5011	557.7542	1113.5171	557.2622	11
3	376.1867	188.5970	**358.1761**	179.5917	F	**1032.4592**	516.7333	1015.4327	508.2200	1014.4487	507.7280	10
4	477.2344	239.1208	**459.2238**	230.1155	T	**885.3908**	443.1991	868.3643	434.6858	867.3803	434.1938	9
5	564.2664	282.6368	546.2558	273.6316	S	**784.3431**	392.6752	767.3166	384.1619	**766.3326**	383.6699	8
6	651.2984	326.1529	633.2879	317.1476	S	**697.3111**	**349.1592**	680.2846	340.6459	679.3006	340.1539	7
7	738.3305	369.6689	720.3199	360.6636	S	**610.2791**	305.6432	593.2525	297.1299	592.2685	296.6379	6
8	825.3625	413.1849	807.3519	404.1796	S	**523.2471**	262.1272	506.2205	253.6139	505.2365	253.1219	5
9	912.3945	456.7009	894.3840	447.6956	S	436.2150	218.6112	419.1885	210.0979	418.2045	209.6059	4
10	999.4265	500.2169	981.4160	491.2116	S	**349.1830**	**175.0951**	332.1565	166.5819	331.1724	166.0899	3
11	1086.4586	543.7329	**1068.4480**	534.7276	S	262.1510	131.5791	245.1244	123.0659	244.1404	122.5738	2
12					R	**175.1190**	88.0631	158.0924	79.5498			1

Table S2C spot 986_ K16

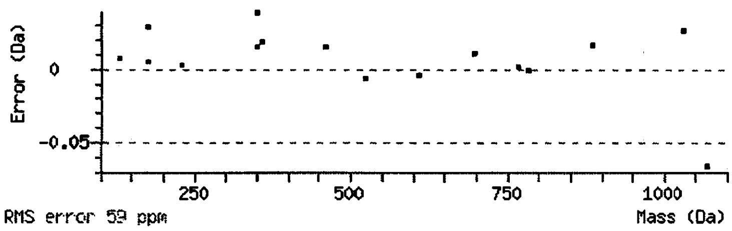

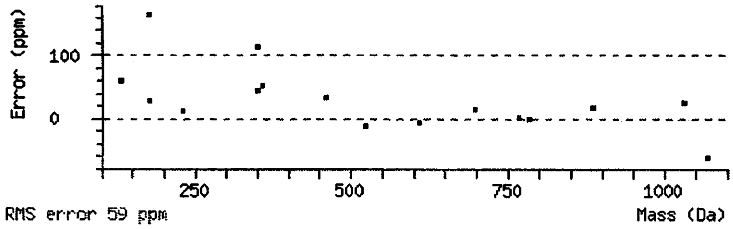

NCBI **BLAST** search of EVFTSSSSSSSR
(Parameters: blastp, nr protein database, expect=20000, no filter, PAM30)
Other BLAST web gateways

All matches to this query

Score	Mr(calc):	Delta	Sequence
45.9	1259.5630	-0.0021	EVFTSSSSSSSR
7.8	1259.6544	-0.0935	LMTISHTTTQK
6.2	1259.6366	-0.0757	MPMNAALGKPSK
3.0	1259.6398	-0.0789	YPDPVIKVNDT
2.4	1259.5816	-0.0208	EIDVHSGTXQK

Mascot: http://www.matrixscience.com/

{MATRIX SCIENCE} Mascot Search Results

Peptide View

MS/MS Fragmentation of **QLSSGVSEIR**
Found in **HHHU27**, heat shock protein 27 - human

Match to Query 23: 1154.628648 from(578.321600,2+) intensity(17044.2000)
Data file C:\Documents and Settings\micromass\Desktop\pkl\otherPKl\VV_URBC_050620_1302_010.pkl

Click mouse within plot area to zoom in by factor of two about that point
Or, Plot from 0 to 1200 Da Full range

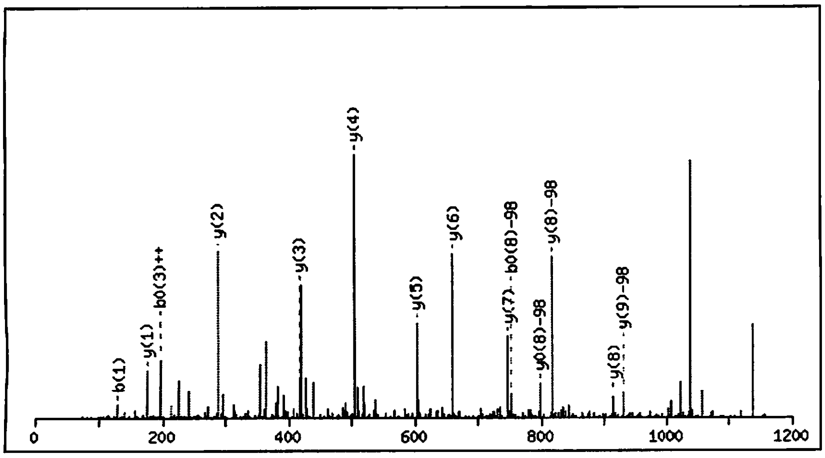

Monoisotopic mass of neutral peptide Mr(calc): 1154.5332
Fixed modifications: Carbamidomethyl (C)
Variable modifications:
S3 : Phospho (ST), with neutral losses 97.9769(shown in table), 0.0000
Ions Score: 71 **Expect:** 4e-005
Matches (Bold Red): 14/154 fragment ions using 25 most intense peaks

#	b	b^{++}	b*	b^{*++}	b^0	b^{0++}	Seq.	y	y^{++}	y*	y^{*++}	y^0	y^{0++}	#
1	**129.0659**	65.0366	112.0393	56.5233			Q							10
2	242.1499	121.5786	225.1234	113.0653			L	**929.5051**	465.2562	912.4785	456.7429	911.4945	456.2509	9
3	311.1714	156.0893	294.1448	147.5761	293.1608	147.0840	S	**816.4210**	408.7141	799.3945	400.2009	**798.4104**	399.7089	8
4	398.2034	199.6053	381.1769	191.0921	380.1928	190.6001	S	**747.3995**	374.2034	730.3730	365.6901	729.3890	365.1981	7
5	455.2249	228.1161	438.1983	219.6028	437.2143	219.1108	G	**660.3675**	330.6874	643.3410	322.1741	642.3570	321.6821	6
6	554.2933	277.6503	537.2667	269.1370	536.2827	268.6450	V	**603.3461**	302.1767	586.3195	293.6634	585.3355	293.1714	5
7	641.3253	321.1663	624.2988	312.6530	623.3147	312.1610	S	**504.2776**	252.6425	487.2511	244.1292	486.2671	243.6372	4
8	770.3679	385.6876	753.3414	377.1743	**752.3573**	376.6823	E	**417.2456**	209.1264	400.2191	200.6132	399.2350	200.1212	3
9	883.4520	442.2296	866.4254	433.7163	865.4414	433.2243	I	**288.2030**	144.6051	271.1765	136.0919			2
10							R	**175.1190**	88.0631	158.0924	79.5498			1

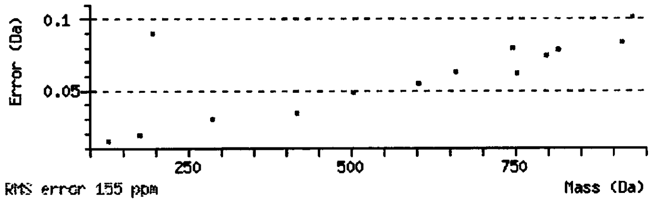

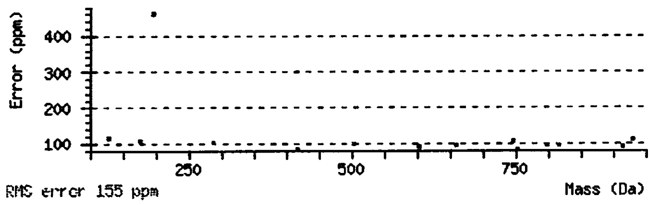

Table S2C spot 1302_P-HSP27

NCBI **BLAST** search of QLSSGVSEIR
(Parameters: blastp, nr protein database, expect=20000, no filter, PAM30)
Other BLAST web gateways

All matches to this query

Score	Mr(calc):	Delta	Sequence
70.5	1154.5332	0.0954	QLSSGVSEIR
55.5	1154.5332	0.0954	QLSSGVSEIR
39.6	1154.5332	0.0954	QLSSGVSEIR
18.5	1154.5696	0.0590	KISTTQIER
11.0	1154.5882	0.0404	LQSMLSKIR
10.9	1154.4969	0.1318	QVTEPTSSAR
10.8	1154.5883	0.0404	KICVTVSLR
10.8	1154.5883	0.0404	KICVTVSLR
10.3	1154.4605	0.1682	QLSEEQDAR
10.0	1154.5696	0.0590	KISTTQIER

Mascot: http://www.matrixscience.com/
